# Pegtarazimod limits acute graft-versus-host disease mortality and severity in mice and is tolerated in patients

**DOI:** 10.1172/JCI205864

**Published:** 2026-08-04

**Authors:** Verena Holzmüller, Jana Gawron, Ann-Cathrin Burk, Anna-Verena Stell, Anna-Sophia Baur, Alexander Zähringer, Viktor Fetsch, Annika Mäder, Alina Hartmann, Nana Talvard-Balland, Neel Krishna, Kenji Cunnion, Ulrich Thienel, Paolo Martini, Lindsey Glenn, James L.M. Ferrara, Monzr M. Al Malki, Hannah Choe, José Antonio Pérez-Simón, Annette Schmitt-Graeff, Joerg Buescher, Natalie Köhler, Zohreh Mansoori Moghadam, Philipp Henneke, Geoffroy Andrieux, Melanie Boerries, Robert Zeiser

**Affiliations:** 1 Department of Internal Medicine I, Medical Center, Faculty of Medicine,; 2 Spemann Graduate School of Biology and Medicine,; 3 Faculty of Biology,; 4 German Cancer Consortium (DKTK), Partner Site Freiburg, a partnership between DKFZ and the Medical Center, and; 5 CIBSS – Centre for Integrative Biological Signalling Studies, University of Freiburg, Freiburg, Germany.; 6 ReAlta Life Sciences, Norfolk, Virginia, USA.; 7 UT Partners, New York, New York, USA.; 8 Icahn School of Medicine at Mount Sinai, New York, New York, USA.; 9 City of Hope National Medical Center, Duarte, California, USA.; 10 Division of Hematology, The James Cancer Hospital and Solove Research Institute, The Ohio State University, Columbus, Ohio, USA.; 11 Hospital Universitario Virgen del Rocío, Seville, Spain.; 12 University of Freiburg, Freiburg, Germany.; 13 Max Planck Institute of Immunobiology and Epigenetics, Freiburg, Germany.; 14 Institute for Infection Control and Prevention and; 15 Institute of Medical Bioinformatics and Systems Medicine, Medical Center, Faculty of Medicine, University of Freiburg, Freiburg, Germany.

**Keywords:** Hematology, Immunology, Bone marrow transplantation

## Abstract

The success of allogeneic hematopoietic cell transplantation (allo-HCT) is limited by acute graft-versus-host disease (aGVHD). We have previously reported that neutrophils can exacerbate tissue damage caused by conditioning regimens. Pegtarazimod is a synthetic peptide, derived from the capsid protein of human astrovirus serotype 1, that was shown to reduce neutrophil effector functions. Therefore, we evaluated the therapeutic activity of pegtarazimod against aGVHD. Pegtarazimod significantly reduced aGVHD-related mortality, histological aGVHD severity, and proinflammatory cytokines in multiple in vivo mouse models while maintaining the antileukemia effect. Mechanistically, pegtarazimod reduced inflammation by decreasing ROS production, as investigated using allo-HCT recipient mice with genetic inactivation of NADPH oxidase in the BM. In addition to the antiinflammatory effect, pegtarazimod protected intestinal organoids against TNF-induced toxicity and oxidative DNA damage. In the phase II clinical trial AURORA, pegtarazimod treatment was well tolerated in patients with corticosteroid-refractory aGVHD (ClinicalTrials.gov NCT06343792), with an overall response rate of 4/7 patients at day 28. In summary, pegtarazimod reduced aGVHD in mice by suppressing proinflammatory neutrophil effector functions and preserving enterocyte integrity. The clinical trial data support tolerability of pegtarazimod in aGVHD patients, and further studies are needed to determine efficacy.

## Abstract 2

This study discovered a new treatment based on an astrovirus-derived peptide for a complication called GVHD that occurs in cancer patients after stem cell transplantation.

## Graphical abstract

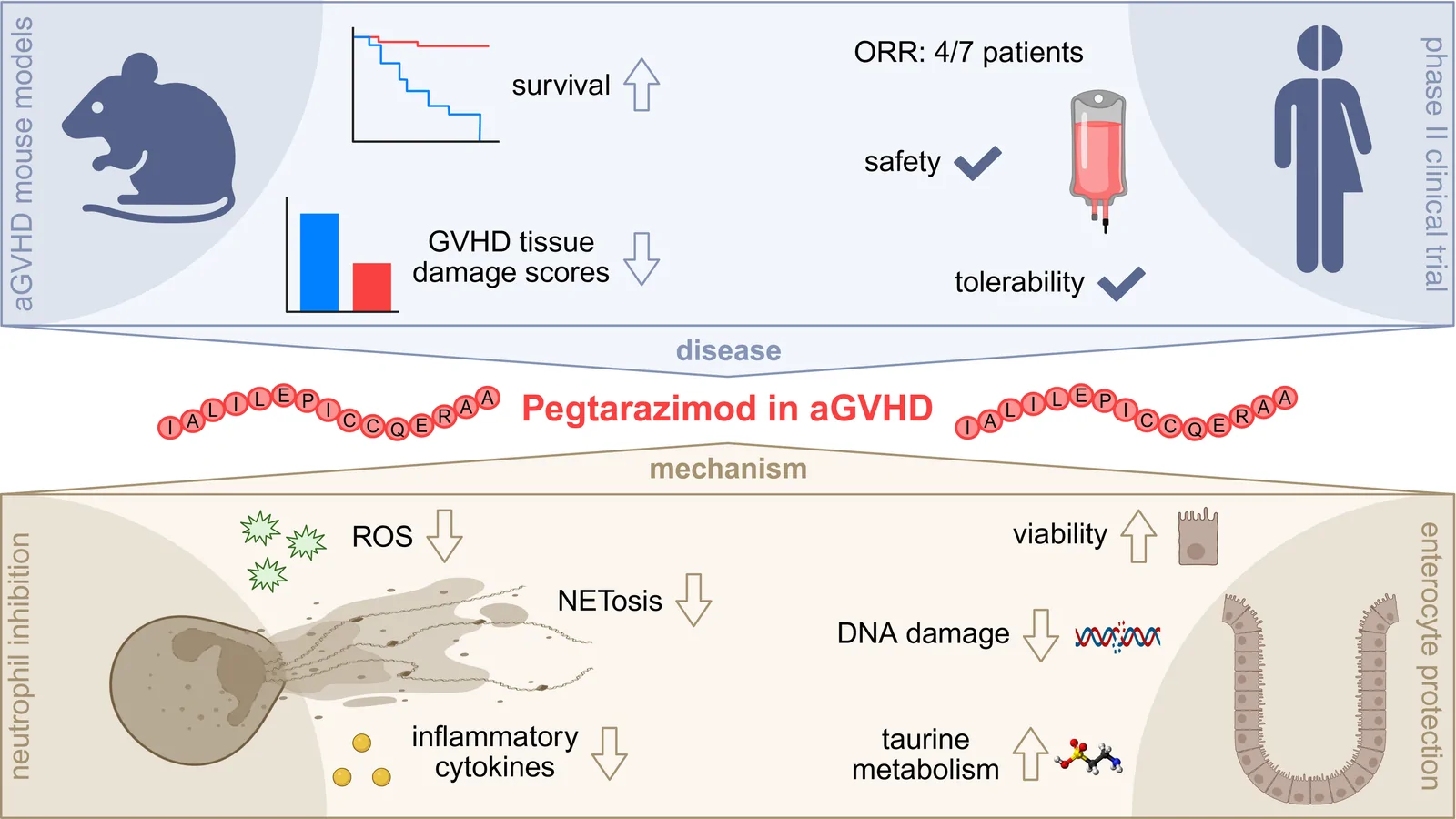

## Introduction

Allogeneic hematopoietic cell transplantation (allo-HCT) is the only curative treatment option for the majority of patients with acute myeloid leukemia (AML) who are eligible for this therapy (1). However, its therapeutic benefit is often limited by severe complications, including acute graft-versus-host disease (aGVHD), infections, and disease relapse (2). Despite immunosuppressive prophylaxis, aGVHD remains the major cause of nonrelapse mortality after allo-HCT. Patients with corticosteroid-refractory aGVHD (SR-aGVHD) especially have a poor prognosis with a mortality rate of 50% in the first 3 years after diagnosis (3). Although ruxolitinib has improved response rates and progression-free survival of patients with SR-aGVHD (4), many patients do not benefit. Therefore, new therapeutic strategies against aGVHD that maintain the graft-versus-leukemia (GVL) effect are needed. While most clinical strategies against aGVHD target T cells, several studies support a role for neutrophils in aGVHD (5–7). The tissue-damaging effect of neutrophils in the early phase of aGVHD was shown to be mediated by the release of ROS (5) and by priming of donor T cells in mesenteric lymph nodes (6). To date, the only available means of targeting neutrophils has been their depletion, which is problematic given their critical role in pathogen control. Therefore, a drug that reduces neutrophil effector functions without depleting them would be desirable for aGVHD treatment.

Pegtarazimod (RLS-0071) is a synthetic peptide that was shown to reduce the activity of the hypochlorous acid–producing enzyme myeloperoxidase (MPO) (8, 9) as well as the formation of neutrophil extracellular traps (NETs) (10). Pegtarazimod is a 15–amino acid peptide with a 24-mer PEG linker on the C-terminus (11). The peptide sequence was derived from the capsid protein of human astrovirus serotype 1 (HAstV-1), which was shown to cause a noninflammatory, self-limiting gastroenteritis (12) by modulating the complement system (11, 13).

In this study, we evaluated the capacity of pegtarazimod as a novel treatment for aGVHD and analyzed the underlying mechanism of action. We observed that pegtarazimod reduced aGVHD-related mortality and decreased histological aGVHD severity in mouse models of aGVHD while preserving the GVL effect. Mechanistically, pegtarazimod reduced inflammation by decreasing oxidative stress and lowering levels of the NET marker neutrophil elastase (NE). In addition, inflammatory cytokines were reduced in splenic neutrophils and in the small intestine (SI) of mice with aGVHD. Apart from the tolerogenic effect on neutrophils, we found that pegtarazimod protected enterocytes against TNF-induced toxicity and oxidative stress, which was connected to reduced DNA damage and increased taurine metabolism of the enterocytes. Based on these promising preclinical experiments, a phase II, open-label clinical trial with pegtarazimod in combination with ruxolitinib for patients with SR-aGVHD was performed (ClinicalTrials.gov NCT06343792), and pegtarazimod treatment was well tolerated.

## Results

### Pegtarazimod improves survival and histopathological severity of aGVHD without impeding the antileukemia effect.

The effect of pegtarazimod on aGVHD was assessed using a previously described allo-HCT mouse model with BALB/c recipient and C57BL/6 donor mice (14). Since pegtarazimod has been reported to inhibit neutrophil effector functions (8, 10) and neutrophils have been shown to contribute to tissue damage in the early phase of aGVHD (5), we hypothesized that aGVHD could be prevented by treating mice daily for 7 days, starting on the day of allo-HCT. We observed reduced mortality in pegtarazimod-treated mice compared with vehicle-treated mice (Figure 1A and Supplemental Figure 1A; supplemental material available online with this article; https://doi.org/10.1172/JCI205864DS1) and decreased aGVHD histopathology scores in the classical aGVHD target organs, SI, colon, and liver (Figure 1, B–D). Typical aGVHD histopathology characteristics, including crypt abscesses and apoptotic enterocytes, were reduced in pegtarazimod-treated mice compared with vehicle-treated mice (Figure 1E). Similar reduction of mortality was shown in another model with BALB/c donors and C57BL/6 recipients to exclude strain-specific effects of pegtarazimod (Figure 1F and Supplemental Figure 1B). To extend this prophylactic approach to a therapeutic one, we next applied a later treatment schedule from days 7 to 14 after allo-HCT, a time period during which mice undergoing allo-HCT manifest clinical signs of aGVHD. Consistent with the previous model, pegtarazimod treatment reduced mortality (Figure 1G and Supplemental Figure 1C) and tissue damage in the SI, colon, and liver (Figure 1, H–J). To investigate the impact of pegtarazimod on the GVL effect, we used an established mouse model that relies on MLL-PTD– and FLT3-ITD–driven AML (15). Leukemia-bearing mice that received additional allogeneic T cells experienced improved survival compared with mice receiving only BM, independent of vehicle or pegtarazimod treatment (Figure 2A). To validate these findings in a second AML model, we used the myelomonocytic AML cell line WEHI-3B. Leukemia-bearing mice that received allogeneic T cells had reduced blast cells in the BM, independent of treatment with vehicle or pegtarazimod (Figure 2B), and pegtarazimod could reduce histological aGVHD severity in the SI, colon, and liver (Figure 2, C–E). Overall, pegtarazimod decreased aGVHD severity in mice after allo-HCT, while preserving the antileukemia effect.

### Pegtarazimod binds to neutrophils and decreases ROS production and NETosis.

Previous studies have reported an effect of pegtarazimod on neutrophils (8, 10). To investigate if pegtarazimod can bind to neutrophils and to decipher the subcellular localization, immunofluorescence (IF) staining was performed on mouse neutrophils. Consistent with a major accumulation on the cell exterior, the pegtarazimod signal was found around the cell surface (Figure 3A). The effect of pegtarazimod on ROS generation, a key neutrophil effector mechanism driven by MPO, was evaluated. Pegtarazimod decreased the enzymatic activity of MPO in an enzyme-based cell-free assay in a dose-dependent manner (Figure 3B). Additionally, pegtarazimod reduced ROS production in PMA-activated murine neutrophils (Figure 3C). The abundance and the activity of MPO were reduced in the serum of mice with aGVHD when treated with pegtarazimod (Figure 3, D and E), while no effect on MPO in T cells was observed (Supplemental Figure 2, A–C). Neutrophils can produce NE and undergo NETosis to react to pathogens or inflammatory conditions (16). Pegtarazimod decreased NE levels in the serum of mice with aGVHD (Figure 3F), and IF staining of NETs in the SI of mice with aGVHD revealed less NETosis when the mice were treated with pegtarazimod (Figure 3, G and H). These findings show that pegtarazimod acts on neutrophils and inhibits proinflammatory effector mechanisms.

### Pegtarazimod decreases the inflammatory cytokine and chemokine profile of neutrophils in vitro and during aGVHD in vivo.

Inflammatory cytokines and chemokines have the capacity to exacerbate aGVHD and are therefore a promising target for therapeutic interventions (17). Neutrophils can produce a broad spectrum of cytokines and chemokines, such as TNF and IL-1β (18, 19). We isolated neutrophils from the BM of naive C57BL/6 mice and observed reduced production of IL-1β pro (Figure 4A), C-C motif chemokine 2 (MCP-1/CCL2) (Figure 4B), and TNF (Figure 4C) upon stimulation with LPS and treatment with pegtarazimod or vehicle in vitro. Consistent with these findings, we observed a reduction of IL-1β pro, MCP-1/CCL2, and TNF in neutrophils derived from the spleen of pegtarazimod-treated compared with vehicle-treated allo-HCT mice (Figure 4, D–I). To confirm the effect of pegtarazimod on IL-1β, we analyzed protein expression of IL-1β in neutrophils isolated from human peripheral blood exposed to LPS and treated with pegtarazimod in vitro. Similar to the effect on mouse neutrophils, pegtarazimod reduced IL-1β production in human neutrophils (Figure 4J). Furthermore, transcript levels of *Il1b* were reduced in the SI of mice with aGVHD, which suggests a direct link between reduced cytokine production upon pegtarazimod treatment and reduced tissue damage in a major aGVHD target organ (Figure 4K). To understand if pegtarazimod has effects on myeloid cell types other than neutrophils, we analyzed the expression of costimulatory molecules and MHC-II on macrophages. We observed a reduction of CD80 expression on pegtarazimod-exposed BMDMs (Supplemental Figure 3, A and B) and macrophages in the spleen of pegtarazimod-treated mice that had undergone allo-HCT (Supplemental Figure 3, E and F). However, we observed no pegtarazimod-dependent differences in expression of CD86 and MHC-II (Supplemental Figure 3, C, D, and G–J) and no influence of pegtarazimod on BMDM-induced activation of allogeneic T cells (Supplemental Figure 3, K and L). Overall, pegtarazimod decreased cytokine expression by neutrophils while having a limited effect on other myeloid cells.

### Pegtarazimod reduces aGVHD beyond its effect on neutrophils.

To assess to which degree the protective effect of pegtarazimod against aGVHD is mediated by its impact on neutrophils, we depleted neutrophils in mice before allo-HCT using an anti-Ly6G antibody (5), which resulted in efficient reduction of neutrophils (Supplemental Figure 4A). Consistent with prior work (5), we observed slightly decreased tissue damage in aGVHD target organs in vehicle-treated mice that were injected with anti-Ly6G compared with isotype (Figure 5, A–C). Additionally, a significant reduction in aGVHD histopathology scores of pegtarazimod-treated mice compared with vehicle-treated mice was seen when neutrophils were depleted with anti-Ly6G (Figure 5, A–C), indicating that the protective effect of pegtarazimod against aGVHD may not be limited to neutrophil inhibition. To elucidate the role of pegtarazimod on neutrophil effector functions, we used an aGVHD mouse model with *p47^phox–/–^* (20) chimera recipient mice that are unable to produce the p47phox subunit of NADPH oxidase (NOX2) in the hematopoietic system (21) and thus only synthesize low amounts of ROS (Supplemental Figure 4, B and C). Similar to neutrophil depletion using anti-Ly6G, the genetic inability to produce large amounts of ROS reduced aGVHD in the SI, colon, and liver of vehicle-treated *p47^phox–/–^* chimera mice compared with vehicle-treated WT recipient mice (Figure 5, D–F). When comparing *p47^phox–/–^* chimera recipients treated with vehicle to WT recipient mice treated with pegtarazimod, similar aGVHD histology scores were observed (Figure 5, D–F), indicating that the inhibition of ROS production may be the predominant effect of pegtarazimod on neutrophil effector functions in aGVHD. Similar to neutrophil-depleted recipient mice, *p47^phox–/–^* chimera recipient mice also benefited from pegtarazimod treatment (Figure 5, D–F), supporting the hypothesis that pegtarazimod may have further beneficial effects on aGVHD beyond inhibition of neutrophil-mediated ROS production.

### Pegtarazimod binds to enterocytes and has a protective effect on enterocytes.

To clarify how pegtarazimod exerts its protective effects against aGVHD in the absence of neutrophils, we tested the concept that pegtarazimod has a tissue-protective effect on epithelial cells. We challenged MODE-K cells, a murine small intestinal epithelial cell line, with menadione to induce oxidative damage. While menadione reduced viability in a dose-dependent manner in vehicle-treated MODE-K cells, pegtarazimod protected from menadione-induced cell death (Figure 6A). To study this effect in an in vitro model that better represents the inflammatory milieu during aGVHD, we generated organoids from the SI of BALB/c mice. When these organoids were challenged with TNF, a cytokine that plays a major role in aGVHD, pegtarazimod improved viability compared with vehicle (Figure 6B and Supplemental Figure 5A) and decreased cytotoxicity measured by lactate dehydrogenase (LDH) release (Figure 6C). Similar to the effect on MODE-K cells, menadione reduced viability of SI organoids, but this effect could be reversed by pegtarazimod treatment (Supplemental Figure 5B). To distinguish the tissue-protective effect of pegtarazimod from the antiinflammatory effect, we depleted TNF using an allo-HCT model and found no influence of anti-TNF on the protective effect of pegtarazimod on histological damage in the SI, colon, and liver (Supplemental Figure 5, C–E). To decipher whether pegtarazimod can bind to enterocytes, we performed IF staining of pegtarazimod-treated SI organoids and observed that pegtarazimod was bound to the organoids (Figure 6, D and E). At higher magnification, we saw that pegtarazimod only binds to the cell surface without penetrating the organoids or entering the cells (Figure 6F). These findings support the hypothesis that, in addition to an antiinflammatory effect, pegtarazimod also has tissue-protective properties.

### Pegtarazimod modulates DNA damage and metabolism of enterocytes.

To characterize the effect of pegtarazimod on intestinal organoids exposed to cell damage, we performed transcriptome profiling of small intestinal organoids challenged with TNF. The principal component analysis (PCA) revealed major differences when comparing organoids exposed to vehicle versus pegtarazimod (Figure 7A). Pegtarazimod caused downregulation of gene expression programs related to chromosome organization and DNA repair (Figure 7B). In agreement, genes associated with DNA damage response, like *Tnf*, were less expressed (Figure 7C). To mechanistically confirm that pegtarazimod protects enterocytes from genomic stress, we challenged MODE-K cells with menadione to induce DNA damage and treated them with pegtarazimod or vehicle. We observed that wells with MODE-K cells treated with pegtarazimod showed higher cell density, confirming a protective effect in the presence of menadione (Figure 7D). We stained MODE-K cells for H2A.X Phospho (Ser139) (γH2A.X), a histone that is phosphorylated when DNA double-strand breaks occur. We observed that pegtarazimod caused a reduction in the proportion of yH2A.X^+^ cells compared with the vehicle group (Figure 7, E and F). This finding supports the concept that pegtarazimod protects enterocytes and reduces DNA damage.

Furthermore, we observed that several pathways associated with cellular metabolism and catabolism were upregulated in the pegtarazimod group compared with the vehicle group (Figure 8A). Untargeted liquid chromatography–mass spectrometry (LC-MS) of lipids and polar metabolites confirmed clear metabolic differences between organoids treated with TNF and vehicle versus TNF and pegtarazimod (Figure 8B). In the treatment group, many polar metabolites and lipids were upregulated compared with the vehicle group (Figure 8, C and D, and Supplemental Figure 6, A and B). We performed pathway analysis with the Mummichog algorithm, which maps LC-MS features to metabolites using the KEGG database and assesses overrepresentation of these metabolites in metabolic pathways. We observed that taurine and hypotaurine metabolism was significantly different between treatment and vehicle groups (Figure 8E), driven by significant increases of cysteine sulfinic acid, hypotaurine, and taurine in the treatment group. Therefore, we next evaluated if taurine is beneficial for aGVHD and observed that taurine treatment reduced histological aGVHD severity in allo-HCT mice (Figure 8, F–H). This supports the concept that pegtarazimod acts as a metabolic modifier by inducing taurine, which has protective effects against aGVHD.

We performed a kinase activity profiling of SI organoids to elucidate the underlying signaling pathways and found an upregulation of several phosphotyrosine kinases (Supplemental Figure 7A) and a downregulation of multiple serine-threonine kinases (Supplemental Figure 7B) upon pegtarazimod treatment. Pathway analysis using metascape (22) revealed an upregulation of RAS signaling, supporting the concept that pegtarazimod can support physiological tissue regeneration (Supplemental Figure 7C).

### Pegtarazimod has minor effects on the intestinal microbiome of mice with aGVHD.

The microbiome can be altered by inflammation and by changes in the fitness and metabolism of enterocytes, playing a major role in the development of aGVHD (23). Therefore, we investigated whether pegtarazimod treatment has an effect on the stool microbiome of mice with aGVHD. We found minor differences in the nonmetric multidimensional scaling (NMDS) analysis (Figure 9A) and similar alpha diversity in the pegtarazimod- and vehicle-treated groups, as measured by Shannon index (Figure 9B) and Simpson index (Figure 9C). The relative abundances of bacterial phyla, classes, and orders revealed no major differences (Figure 9, D and E), but a significant reduction of Paraprevotellaceae could be observed (Figure 9, F and G). Prior work has linked Prevotella to worse outcomes in mice and humans with aGVHD (24, 25).

### Pegtarazimod shows promising preliminary results in aGVHD patients (AURORA trial).

AURORA is an international, multicenter, phase II, open-label trial (ClinicalTrials.gov NCT06343792) investigating pegtarazimod as an adjunctive therapy for the treatment of SR GI-aGVHD (aGVHD affecting the colon and ileum). Pegtarazimod was given concomitantly with ruxolitinib in all patients. As per study protocol, pegtarazimod was added to ruxolitinib within 0–5 days of ruxolitinib start. Across Europe and the United States, 8 patients have been enrolled so far (Supplemental Table 1). Pegtarazimod was well tolerated with no severe adverse events related to the study drug (Supplemental Table 2). Overall response rate on day 28 was 57%, with 29% of evaluable patients reaching complete response (Figure 10A). The aGVHD overall clinical grades declined in some patients during and after the 7-day pegtarazimod treatment period (Figure 10B). Several patients experienced a decrease in aGVHD stages of the skin (Figure 10C), upper GI (Figure 10D), lower GI (Figure 10E), or liver (Figure 10F) during and after the 7-day pegtarazimod treatment period. The responses were seen even though treatment was only for 7 days in this patient cohort. Furthermore, we observed a trend toward reduced MPO plasma levels during the 7-day pegtarazimod treatment period, while they increased after the treatment was ended in some patients (Figure 10G). We measured cytokine levels in patient plasma and found a reduction of IFN-γ during the pegtarazimod treatment period (Supplemental Figure 8A). Conversely, IL-12p70, TNF, MCP-1/CCL2, IL-1β, IL-6, NE, and the complement marker C5b-9 were unchanged (Supplemental Figure 8, B–H). Overall, these results of the AURORA trial reveal no new safety signals and indicate clinical responses in some patients to the combination of ruxolitinib and pegtarazimod.

## Discussion

The severity of aGVHD determines the outcome of patients after allo-HCT, in particular when the intestinal tract is involved (3). While classical approaches to prevent aGVHD rely on targeting allogeneic T cell activation, e.g., by calcineurin inhibition (2), regenerative approaches such as glucagon like peptide-2 (26–28), gastrin (29), R-spondin (30), and IL-22 (31) have emerged recently. Targeting myeloid cell–derived tissue damage without impairing leukemia-reactive T cells is a major unmet goal in aGVHD therapy, given the dual roles of myeloid populations in both driving inflammation (5, 6) and at later stages promoting tissue regeneration (32) in mice. In patients, the numbers of neutrophils infiltrating the intestines are associated with transplant-related mortality (33). However, neutrophils are crucial to control infections that pose a high risk for patients after allo-HCT (34). Therefore, modulation of neutrophil effector functions rather than depleting them entirely could represent a promising strategy to treat aGVHD.

Here, we report that pegtarazimod is effective in both preventing and treating aGVHD in mice while preserving the GVL effect of allo-HCT. Pegtarazimod is a peptide derived from the capsid protein of HAstV-1, which was shown to cause noninflammatory, self-limiting gastroenteritis in patients (12) by inhibiting complement activation and decreasing ROS production (8, 9) and NET formation (10). ROS have been shown to contribute to aGVHD (5), and ROS scavenging has proven potential in preclinical studies (35–37), while NETosis has been associated with ocular GVHD (38, 39). In mouse models of aGVHD, pegtarazimod inhibited ROS production and NETosis both in the serum of mice and in the SI, linking decreased neutrophil effector functions to less histological damage in a major aGVHD target organ. The preponderance of experimental data supports a mechanistic role for MPO/NET targeting by pegtarazimod. Neutrophils can release a variety of proinflammatory cytokines like TNF and IL-1β (40), which are involved in T cell activation in aGVHD (41–43). Pegtarazimod could inhibit the production of TNF, MCP-1/CCL2, and IL-1β in neutrophils both in vitro and in vivo in aGVHD and decreased transcriptional levels of *Il1b* in the SI of mice with aGVHD, providing another link between neutrophils and reduced histological damage. Previous studies have reported that inhibition of the costimulatory molecule CD80 can improve aGVHD (44, 45). Thus, the capacity of pegtarazimod to decrease CD80 expression on macrophages might also contribute to the protection against aGVHD. Consistent with previous reports in the literature (5), both neutrophil depletion by anti-Ly6G and genetic depletion of ROS in allo-HCT recipients decreased aGVHD. Consistent with that, we showed that pegtarazimod treatment could lower aGVHD in a similar manner. Pegtarazimod could also protect from aGVHD in the absence of neutrophils and p47phox, leading to low ROS levels, suggesting an effect of pegtarazimod that goes beyond inhibition of myeloid cells and indicating a protective effect on enterocytes. When we induced oxidative damage in the enterocyte cell line MODE-K and TNF-induced toxicity in a previously reported SI organoid model (46), we observed increased viability and reduced DNA damage upon pegtarazimod treatment, pointing toward a tissue-protective effect of pegtarazimod. Previous studies have highlighted the implications of microbiome-derived metabolites during aGVHD (47, 48). Although the effect of pegtarazimod on the intestinal microbiome was limited in our study, we observed increased taurine and hypotaurine metabolism upon pegtarazimod treatment. Taurine is a potent antioxidant synthesized from cysteine (49) and was shown to be protective for intestinal epithelial cells (50). Taurine and hypotaurine abundance is reduced in serum of patients before allo-HCT who later develop aGVHD (51), and prophylactic administration of the taurine-conjugated bile acid tauroursodeoxycholic acid reduces aGVHD severity in mice by reducing antigen presentation and apoptosis of the intestinal epithelium (52). We observed reduced aGVHD severity in mice treated with taurine. This indicates that modulation of taurine and hypotaurine metabolism might contribute to the protective effect that pegtarazimod exerts in aGVHD. Kinase profiling linked the tissue-protective effects of pegtarazimod to increased RAS signaling, a crucial pathway in intestinal epithelial homeostasis (53).

In summary, we present results of AURORA, a phase II clinical trial investigating the safety and efficacy of pegtarazimod treatment for SR-aGVHD patients. Pegtarazimod reduced aGVHD in mice through the reduction of proinflammatory neutrophil effector mechanisms and tissue-protective effects on enterocytes. The clinical trial data indicate that pegtarazimod can be applied in patients with aGVHD in addition to ruxolitinib. Further studies are needed to determine efficacy compared with ruxolitinib alone in patients with lower intestinal tract aGVHD.

## Methods

### Sex as a biological variable.

The AURORA trial examined male and female patients. Several mouse studies were performed with male and female animals.

### AURORA trial.

Eligible patients were > 12 years old, weighed a minimum of 40 kg, and had clinically confirmed SR GI-aGVHD (MAGIC grade II–IV) after allo-HCT. Patients had received treatment with systemic glucocorticoids, and pegtarazimod was administered within the first 48 h after ruxolitinib was initiated. Key exclusion criteria included treatment with any other systemic GVHD therapy and failed allo-HCT due to relapse of the underlying malignant disease. All patients received 10 mg/kg pegtarazimod once daily for 7 days. Primary outcome measures included the number of participants with treatment-related adverse events and treatment-emergent serious adverse events until day 180, as well as the overall response rate of evaluable patients to pegtarazimod until day 28. Reasons for missing values can be found in the Supporting Data Values file, and further details can be found in the study protocol in the supplemental material.

### Mice.

C57BL/6 (H-2Kb) and BALB/c (H-2Kd) mice were purchased either from Janvier Labs (France) or obtained from the animal facility at the University of Freiburg Medical Center. B6.SJL-*Ptprc^a^Pepc^b^*/BoyCrl (Ly5.1, CD45.1) mice were originally purchased from Charles River Laboratories. BM from B6N.129S2-Ncf1tm1Shl/J (*p47^phox–/–^*) mice was provided in-house. All mouse strains were bred at the University of Freiburg Medical Center. All mice were housed under specific pathogen–free conditions. Mice were between 6 and 10 weeks of age and weighed 15–25 g, and only female or male donor/recipient pairs were used.

### aGVHD mouse models.

Major MHC-mismatch models were used for all experiments. In the first model, BALB/c recipients were lethally irradiated using a cesium source with 2 doses of 5 Gy each, given at least 4 h apart. BM and T cells from C57BL/6 donors were isolated from leg bones and the spleen, respectively. Donor T cells were purified using a Pan T cell isolation kit (Miltenyi Biotec). All kits are listed in Supplemental Table 3. After the second irradiation, recipients were injected with 5 × 10^6^ donor BM and 4 × 10^5^ donor T cells. In the second model, C57BL/6 recipients were irradiated with 2 doses of 6 Gy each and injected intravenously with 5 × 10^6^ BM and 1 × 10^6^ T cells from BALB/c donors. Recipients were treated intraperitoneally with 200 μL of 80 mg/mL pegtarazimod daily from days 0 to 7 after transplantation in the prevention model or from days 7 to 14 in the early intervention model. Control mice received 200 μL of 42 mM PEG as vehicle. All reagents are listed in Supplemental Table 4. The neutrophil depletion model was performed similarly to the prevention model with BALB/c recipients described above, and neutrophils in recipients were depleted with 500 μg of InVivoMAb anti-mouse Ly6G (Bio X Cell) on day –1 and 250 μg on day 3. InVivoMAb rat IgG2a isotype control, anti-trinitrophenol (Bio X Cell) was used as isotype. The TNF depletion model was performed similarly to the prevention model with BALB/c recipients described above, and TNF in recipients was depleted with 125 μg of anti-mouse TNF-α (Leinco Technologies) on days –1, 1, 3, and 5. Armenian Hamster IgG Isotype Control (Leinco Technologies) was used as isotype. Antibodies used for aGVHD mouse models are listed in Supplemental Table 5. For the *p47^phox–/–^* chimera model (Supplemental Figure 2B), CD45.1 recipients were irradiated with 2 doses of 6 Gy each and injected intravenously with 5 × 10^6^ BM cells derived from *p47^phox–/–^* or WT C57BL/6 donors, both expressing CD45.2. Engraftment was analyzed every week by flow cytometry and considered sufficient when >80% of CD45^+^ cells expressed CD45.2. After 4–5 weeks, chimera recipients were irradiated with 2 doses of 6 Gy each and injected with 5 × 10^6^ BM and 1 × 10^6^ T cells from BALB/c donors. Recipients were treated as described above for the prevention model. For the taurine treatment model, BALB/c recipients were irradiated and transplanted with allogeneic C57BL/6 BM and T cells as described above for the prevention model. From day –7 to 7, recipients received 200 mM taurine supplementation in drinking water, while control mice received normal drinking water. Mice were monitored daily and scored for clinical symptoms (weight loss, ruffled fur, skin GVHD, deviations in posture, diarrhea) twice a week. At the end of experiments, blood was drawn by retro-orbital bleeding, and organs of interest were collected and immediately frozen or fixed with formaldehyde.

### GVL mouse models.

For the first AML model, we used splenocytes from mice carrying the mutations MLL-PTD and FLT3-ITD. C57BL/6 mice were lethally irradiated with 2 × 6 Gy and injected intravenously with 5 × 10^6^ allogeneic BALB/c BM cells and 5,000 primary AML^MLL-PTD^ ^FLT3-ITD^ cells. Two days later, T cells from BALB/c mice were isolated from the spleen using a Pan T cell isolation kit (Miltenyi Biotec), and 3 × 10^4^ T cells were injected intravenously in C57BL/6 recipients. As AML-only control, PBS was injected. Recipients were treated intraperitoneally with 200 μL of 80 mg/mL pegtarazimod daily from days 0 to 7 after injection of BM and AML^MLL-PTD^ ^FLT3-ITD^ cells. For the second AML model, WEHI-3B cells were used. BALB/c mice were irradiated with 2 × 5 Gy and injected intravenously with 5 × 10^6^ allogeneic C57BL/6 BM and 5,000 WEHI-3B cells. Two days later, T cells from C57BL/6 mice were isolated as described above, and 2 × 10^5^ T cells were injected intravenously in BALB/c recipients. As AML-only control, PBS was injected. Recipients were treated intraperitoneally with 200 μL of 80 mg/mL pegtarazimod daily from days 0 to 7 after injection of BM and WEHI-3B cells. To measure tumor load, mice were euthanized on day 14, and BM smear was dried overnight and stained with May-Grünwald and Giemsa to evaluate blast count.

### aGVHD histopathology scoring.

Samples from SI, colon, and liver were harvested, fixed in 4% formaldehyde overnight, and transferred to 50% EtOH. 3-μm-thick tissue sections were prepared after dehydration using a Leica TP1020 and Leica EG1150 tissue embedding center and stained with H&E with a Leica Infinity H&E Staining system. Samples were scored for histopathological aGVHD symptoms by an experienced pathologist blinded to the treatment groups. aGVHD severity was assessed using a previously described scoring system (32). Intestinal aGVHD was scored based on crypt apoptosis (0, rare to none; 1, occasional apoptotic bodies per 10 crypts; 2, few apoptotic bodies per 10 crypts; 3, the majority of crypts contain an apoptotic body; 4, the majority of crypts contain > 1 apoptotic body) and inflammation (0, none; 1, mild; 2, moderate; 3, severe, without ulceration; 4, severe, with ulceration). Liver aGVHD was scored based on the tissue involved (0, none; 1, few involved tracts; 2, numerous involved tracts; 3, injury of the majority of tracts; 4, severe involvement of most tracts).

### IF staining.

For IF staining, neutrophils were isolated from mouse BM as described below, fixed with 4% formaldehyde, and blocked and permeabilized with 5% BSA (Carl Roth), 5% goat serum (Biozol), 5% horse serum (Merck), and 0.1% Triton X-100 (Merck). Tissue sections were prepared as described above; antigen retrieval was performed using Target Retrieval Solution, Citrate pH 6 (Dako); and blocking and permeabilization were accomplished using 5% BSA, 5% goat serum, and 0.1% Triton X-100. Intestinal organoids were fixed with 4% formaldehyde and blocked and permeabilized with 0.2% BSA and 0.1% Triton X-100. MODE-K cells were fixed with 4% formaldehyde and blocked and permeabilized with 5% BSA and 0.1% Triton X-100. Neutrophils were stained with mouse-anti-TUBA4A (Merck) and rabbit anti-pegtarazimod primary antibodies and donkey anti-mouse IgG H&L Alexa Fluor 647 (Thermo Fisher Scientific) and goat anti-rabbit IgG H&L Alexa Fluor 568 (Abcam) secondary antibodies. Tissue sections were stained with rabbit anti-histone H3 (citrulline R2 + R8 + R17) (Abcam) and rat anti-MPO (Abcam) primary antibodies and goat anti-rat IgG H&L Alexa Fluor 488 (Thermo Fisher Scientific) and goat anti-rabbit IgG H&L Alexa Fluor Plus 647 (Thermo Fisher Scientific) secondary antibodies. Intestinal organoids were stained with rabbit anti-pegtarazimod primary and goat anti-rabbit IgG H&L Alexa Fluor 488 (Abcam) secondary antibody and with Alexa Fluor 647 Phalloidin labeling probe (Thermo Fisher Scientific). MODE-K cells were stained with mouse anti-H2A.X Phospho (Ser139) Alexa Fluor 488 (BioLegend). Nuclei were stained with DAPI (Merck). Neutrophils were imaged with a Zeiss LSM880 (plan apochromat ×63/1.4 oil DIC M27), tissue sections with a Zeiss LSM710 (plan apochromat ×63/1.4 oil DIC M27), organoids with a Zeiss LSM710 (plan apochromat ×20/0.8 M27 or plan apochromat ×63/1.4 oil DIC M27), and MODE-K cells with a Zeiss LSM880 (plan apochromat ×63/1.4 oil DIC M27) confocal microscope. Tissue sections and MODE-K cells were imaged with an Olympus ScanR (UPlanSApo ×10/0.4 air) wide-field microscope for quantification. Antibodies used for IF staining are listed in Supplemental Table 6.

### MPO activity and respiratory burst assays.

For a plate-based MPO activity assay, 0.4 μg of human MPO (Merck) was incubated with pegtarazimod for 5 min at room temperature (RT), followed by incubation with TMB for 2 min at RT. Reaction was stopped using 2 N H_2_SO_4_, and absorbance was measured at 450 nm. For a cell-based respiratory burst assay, BM was isolated from C57BL/6 mice and incubated with pegtarazimod in RPMI 1640 (Thermo Fisher Scientific) supplemented with 1% BSA and 1 mM CaCl_2_ for 2 h at 37 °C, followed by incubation with 0.5 μg/mL dihydrorhodamine 123 (DHR123) for 15 min at 37°C. After activation with 1 μM PMA for 45 min at 37°C, cells were stained for flow cytometry as described below. ROS convert nonfluorescent DHR123 to fluorescent rhodamine 123. Therefore, rhodamine 123 in neutrophils was analyzed using flow cytometry as a readout for respiratory burst. MPO activity in the serum of mice with aGVHD was measured using the MPO Peroxidation Activity Assay Kit (Fluorometric) (Abcam). If the calculated activity was negative, the value was set to 0.

### ELISA.

MPO and NE levels in the serum of mice with aGVHD were measured using the Mouse MPO ELISA Kit (Abcam) and the Mouse NE ELISA Kit (Abcam), respectively.

### Quantification of NETs.

SI tissue sections were stained for citrullinated histone H3, MPO, and DAPI as described above. Samples were imaged and analyzed using the Olympus ScanR system and ScanR 3.5 software. Cells were defined by a circular shape, and doublets were excluded. NETs were defined by a high citrullinated histone H3 signal and high standard variation of the MPO signal to account for granularity of neutrophils. For quantification, the number of NETs was divided by the number of single cells.

### Flow cytometry.

For all stainings, cells were isolated and washed with PBS. In spleen or BM cells, erythrocytes were lysed using erythrocyte lysis buffer (0.5 M NH_4_Cl, 10 mM KHCO_3_, 1 mM EDTA, all Carl Roth). Viability was assessed with Zombie NIR fixable dye (BioLegend) or LIVE/DEAD Fixable Aqua (Thermo Fisher Scientific). Fc receptors were blocked with anti-mouse CD16/32 (BioLegend) or anti-human FcR Blocking Reagent (Miltenyi) for 10 min at 4°C, followed by surface staining with fluorochrome-conjugated antibodies for 20–30 min at 4°C in PBS supplemented with 2.5 mM EDTA, 2% FCS (Anprotec), and 0.02% NaN_3_ (Carl Roth). If multiple antibodies conjugated to BV or BUV fluorophores were used, staining was performed in BD Horizon Brilliant Stain Buffer (BD Biosciences). Cells were fixed and permeabilized for 20–45 min at 4°C at RT using the Transcription Factor Staining Buffer Set (Thermo Fisher Scientific) or Cytofix/Cytoperm Fixation/Permeabilization Kit (BD Biosciences) for staining of intranuclear proteins or cytoplasmic proteins and cytokines, respectively. Intracellular staining was performed in the respective permeabilization buffer for 30 min at 4°C. Cells were washed twice with permeabilization buffer, measured using a LSR Fortessa flow cytometer (BD Biosciences) or ID7000 (Sony) spectral flow cytometer, and analyzed using FlowJo v10.10 (BD Biosciences). Antibodies used for flow cytometry are listed in Supplemental Table 7.

### Neutrophil in vitro culture.

Mouse neutrophils were isolated based on the protocol by McGill et al. (54). In short, neutrophils from C57BL/6 BM were isolated using a neutrophil isolation kit (Miltenyi Biotec). Neutrophils (purity over 80%) were cultured in RPMI 1640 supplemented with penicillin/streptomycin (Thermo Fisher Scientific) and 0.1% BSA (Carl Roth) and treated with 5 mg/mL pegtarazimod or 2.625 mM vehicle and 100 ng/mL LPS for 1 h at 37°C and 5% CO_2_, followed by analysis using flow cytometry. For human neutrophils, blood was drawn from healthy individuals and incubated with 1:1,000 GolgiPlug Protein Transport Inhibitor (containing brefeldin A, BD Biosciences), 5 mg/mL pegtarazimod or 2.625 mM vehicle, and 100 ng/mL LPS for 1 h at 37°C and 5% CO_2_. Human neutrophils were defined as CD11b^+^, CD66b^+^, CD14^lo^, CD11b^+^, and CD16^+^ cells using flow cytometry.

### Organoid culture and challenge.

Mouse SI organoids were isolated and cultured as previously described (26). In short, the SI was isolated from healthy BALB/c mice, and villi were scraped off. SI was washed with PBS, followed by incubation in 2 mM EDTA in PBS for 30 min at 4°C, and dissociated crypts were filtered through a 70 μm Easystrainer (Greiner Bio One). 50 μL domes of a 1:1 mixture of Matrigel GFR Basement Membrane (Merck) and IntestiCult organoid growth medium (StemCell Technologies) were cultured with 450 μL of IntestiCult organoid growth medium and incubated at 37°C and 5% CO_2_. Medium was changed every 23 days, and organoids were passaged every 5–7 days. For cytokine challenge, the medium was changed 3 days after passaging and supplemented with 5 mg/mL pegtarazimod or 2.625 mM vehicle and 20 ng/mL recombinant murine TNF (Thermo Fisher Scientific). After incubation for 2 days, LDH release was analyzed using the LDH-Glo Cytotoxicity Assay (Promega), viability was assessed using flow cytometry, gene expression was investigated using microarray, metabolites were analyzed using LC-MS, and kinases were profiled by PamDx. To induce oxidative damage, organoids 3 days after passaging were challenged with 10–100 μM menadione and treated with 5 mg/mL pegtarazimod or 2.625 mM vehicle for 3 h. Viability was assessed using flow cytometry. For IF microscopy, organoids were treated with 5 mg/mL pegtarazimod or 2.625 mM vehicle for 24 h, 1 day after passaging.

### Microarray analysis.

Organoids were cultured as described above. RNA was isolated using the RNeasy Micro Kit (Qiagen). RNA integrity was measured using RNA ScreenTape Analysis (Agilent), and samples were processed using the GeneChip WT PLUS Kit (Thermo Fisher Scientific). Microarray analysis was performed using a Clariom S Assay, mouse (Thermo Fisher Scientific). Data analysis was performed in R (v4.4.0). Raw CEL files were processed using the oligo package (v1.68.2) (55) and normalized with the robust multiarray average algorithm, followed by log_2_ transformation. Differential gene expression analysis was conducted with limma (v3.65.1) (56) using a paired design to account for interexperimental variability. Genes were ranked according to their differential expression score, defined as (log_2_FC) × (–log_10_ *P* value), and subsequently used for GSEA with ClusterProfiler (v4.12.6) (57), referencing the MSigDB (58) mouse hallmark gene sets. Genes with an adjusted *P* value (Benjamini-Hochberg correction) below 0.05 were considered statistically significant.

### qPCR.

RNA was isolated using the RNeasy Mini Kit (Qiagen) or the RNeasy Micro Kit (Qiagen), and 500 ng was used for reverse transcription of cDNA using the High-Capacity cDNA Reverse Transcription Kit (Thermo Fisher Scientific). RT-qPCR was performed on a LightCycler 480 (Roche) using the LightCycler 480 SYBR Green I Master (Roche) with 45 amplification cycles. Samples were analyzed using the Advanced Relative Quantification module of the LightCycler 480 software 1.5.1.62 (Roche) and the ΔΔCt method, with normalization to *Hprt* and *Gapdh*. Primers used for qPCR are listed in Supplemental Table 8.

### MODE-K viability and DNA damage.

MODE-K cells, an immortalized epithelial cell line from mouse SI, were provided by D. Kaiserlian (INSERM, Paris, France) and cultured in RPMI 1640 supplemented with 10% FCS (Merck), 100 U/mL penicillin (Thermo Fisher Scientific), 100 μg/mL streptomycin (Thermo Fisher Scientific), 1% GlutaMAX supplement (Thermo Fisher Scientific), 1% MEM nonessential amino acids solution (100×) (Thermo Fisher Scientific), 1 mM Sodium Pyruvate (Thermo Fisher Scientific), 10 mM HEPES (Thermo Fisher Scientific), and 50 μM 2-mercaptoethanol (Thermo Fisher Scientific). To investigate the influence of pegtarazimod on viability, MODE-K cells were challenged with menadione and treated with 5 mg/mL pegtarazimod or 2.625 mM vehicle 1 day after passaging. After incubation for 2 h and 2 h of recovery in medium at 37°C and 5% CO_2_, viability was assessed by flow cytometry. To investigate DNA damage, MODE-K cells were challenged with menadione and treated with pegtarazimod or vehicle as described above, followed by IF staining and microscopy. Expression of γH2A.X was assessed using the Olympus ScanR system. Cells were detected by DAPI staining, doublets were excluded, and yH2A.X^+^ cells divided by the number of single cells were quantified.

### Metabolomics.

Organoids were cultured as described above. Polar metabolites and lipids were extracted using a 2-phase methanol/chloroform/water extraction method adapted from ref. 59. In brief, matrigel domes containing organoids were detached, pelleted, and washed with 0.9% NaCl. Empty matrigel domes served as blanks. Pellets were frozen in liquid N_2_ and resuspended in 300 μL of a 50:50:1 mix of methanol/H_2_O/universal internal standard (200 ppb aminoterephtalic acid, 200 ppb chlorophenylalanine, 1 ppb dioctylamine, 2 ppb didodecylamine, 20 ppm ibuprofen, 40 ppb 16:0 dodecanoyl PE), and 150 μL chloroform was added. Samples were vortexed for 3 min and centrifuged at 20,000*g* for 2 min to separate the aqueous phase containing polar metabolites from the organic phase containing lipids. Nontargeted measurement of lipids by LC-MS was performed on an Agilent 1290 Infinity II UHPLC inline with a Bruker timsTOFflex MS operating in negative or positive ion mode adapting a previously described method (60). Briefly, scan range in PASEF mode was from 60 to 1,350 Da (in positive mode) or 100–1,700 Da (in negative mode). Mass and collision cross-section calibrations were performed at the end of each run. LC separation was on a Zorbax Eclipse plus C18 column (100 × 2 mm, 1.8 μm particles) using a solvent gradient of 70% buffer A (10 mM ammonium formate in 60:40 acetonitrile/water) to 97% buffer B (10 mM ammonium formate in 90:10 2-propanol/acetonitrile). Flow rate was 400 μL/min, autosampler temperature was 5°C, and injection volume was 2 μL. Polar metabolites were analyzed by nontargeted LC-MS metabolomics using an Agilent 1290 Infinity II UHPLC inline with a Bruker Impact II QTOF-MS operating in positive or negative ion mode. Scan range was from 20 to 1,050 Da. Mass calibration was performed at the beginning of each run. LC separation was on a Waters Atlantis Premier BEH ZHILIC column (100 x 2.1 mm, 1.7 μm particles), buffer A was 20 mM ammonium carbonate and 5 μM medronic acid in milliQ H_2_O, buffer B was 90:10 acetonitrile/buffer A, and thesolvent gradient was from 95% to 55% buffer B over 14 min. Flow rate was 180 μL/min, column temperature was 35°C, autosampler temperature was 5°C, and injection volume was 3 μL. Data processing, including detection, deconvolution, and identification of features, was carried out using Metaboscape (version 2025). Missing values for features detected in at least 50% of samples were imputed as half-minimum value of the respective metabolite. For PCA, quantile normalized and missing value–imputed data were used and filtered for features that have been detected above blank, followed by square root scaling. For pairwise differences between pegtarazimod- and vehicle-treated samples, *P* values were computed from quantile normalized data by unpaired 2-sided *t* test assuming unequal variance. Pathway analysis of polar metabolites was carried out in MetaboAnalyst (6.0) using the Mummichog algorithm and *Mus musculus* KEGG pathway library. Mummichog maps features to potential metabolites via KEGG pathways and uses permutation-derived *P* values for enrichment without requiring identification. The enrichment factor shows hit frequency relative to the expected number based on pathway size but does not indicate if pathways are up- or downregulated.

### Microbiome sequencing.

For 16S ribosomal DNA (rDNA) sequencing, 50–100 mg of stool from BALB/c mice in the aGVHD prevention model was collected at day 7. Azenta Life Sciences performed genomic DNA extraction, quality control, rDNA variable region amplification, library construction, high-throughput sequencing, and part of the data analysis. Alpha diversity was assessed using Shannon and Simpson indices. Beta diversity was analyzed using Qiime (1.9.1), and NMDS was performed based on the beta diversity distance matrix. Sankey diagrams were generated with SankeyMATIC and included bacteria on the phylum, class, and order level with a relative abundance of > 1%.

### Statistics.

For the sample size in the murine survival experiments, a power analysis was performed. A sample size of at least *n* = 10 per group was determined by 80% power to reach a statistical significance of 0.05 to detect an effect size of at least 1.06. Differences in animal survival (Kaplan-Meier survival curves) were analyzed by the log-rank test. Survival experiments were performed in a nonblinded fashion. Histological analysis was done in a blinded fashion. All data were tested for normality by applying the Shapiro-Wilk test. Outliers were identified using Grubbs’ outlier testing with α = 0.01. For analysis of 2 groups, an unpaired 2-tailed *t* test was applied, with Welch’s correction in case of unequal standard deviation. For paired data, a paired 2-tailed *t* test or Wilcoxon’s test was used for normally and nonnormally distributed data, respectively. If the data were not normally distributed, a Mann-Whitney test was applied. For comparison of more than 2 groups, we used an ordinary 1-way ANOVA with Holm-Šidák multiple-comparison test for normally distributed data and the Kruskal-Wallis test with Dunn’s multiple testing for nonnormally distributed data. Statistical analysis was performed using GraphPad Prism 10. The number of samples per group is indicated in the figures and represents independent biological replicates unless stated otherwise. Data represent mean ± SEM. A *P* value < 0.05 was considered significant.

### Study approval.

The AURORA study was conducted in accordance with the Declaration of Helsinki (61) and applicable Good Clinical Practice guidelines. Written informed consent using an IRB-approved protocol was obtained from all participants who contributed data to the cohort in accordance with the Declaration of Helsinki (61). The study is registered with ClinicalTrials.gov (NCT06343792). Animal studies were carried out in compliance with relevant animal-use guidelines and ethical regulations. All protocols were approved by the Regierungspräsidium Freiburg, Germany (protocol approval numbers X-20/06K and G-23/061).

### Data availability.

Values for all data points in graphs can be found in the Supporting Data Values file. Microarray data are available in the Gene Expression Omnibus (GSE315947), microbiome data are available in the Sequence Read Archive (PRJNA1399978), and metabolomics data are available in the MassIVE repository (MSV000100513).

## Author contributions

VH designed studies, conducted experiments, acquired data, analyzed data, and wrote the manuscript. JG, ACB, AVS, ASB, AZ, VF, AM, AH, and NTB helped conduct experiments. N Krishna, KC, UT, PM, LG, JLMF, and N Köhler discussed the data and helped with interpretation. MMAM, HC, and JAPS acquired data for the clinical study. ASG, GA, and MB analyzed data. JB acquired and analyzed data. PH and ZMM provided *p47^phox–/–^* mice and helped with experimental planning. RZ designed studies, helped with data analysis, and wrote the manuscript.

## Conflict of interest

RZ received honoraria from Novartis, Therakos, Incyte, medac, Neovii, Sanofi and Therakos. ReAlta Life Sciences provided pegtarazimod and financed the clinical trial but not the preclinical experiments.

## Funding support

- DFG (German Research Foundation) SFB-1479, project ID 441891347 (P01 and S02 to RZ; S01 to MB).
- DFG CRC1160, project ID 256073931 (B0 to RZ and N Köhler, B11N to RZ, and Z02 to MB).
- DFG TRR 167, project ID 259373024 (to RZ and N Köhler; Z01 to MB).
- DFG TRR 353/1, project ID 471011418 (SP02 to MB).
- DFG TRR 359, project ID 491676693 (Z01 to MB).
- DFG RU5659 TARGET-MPN: ZE 872/6-1 (TP 7) (project ID 517204983; to RZ).
- TRR 417 (project ID 540805631; S03 to MB).
- FOR 5476 UcarE (project ID 493802833-P7; to MB).
- Gottfried Wilhelm Leibniz program (DFG) number ZE 872/7-1 (to RZ).
- European Union, proposal number ERC-2022-ADG, project ID 101094168, AlloCure (European Research Council advanced grant to RZ).
- ERA-NET Transcan, PIXEL (to RZ).
- ERA-NET Transcan, SmartCART (to RZ).
- Germany’s Excellence Strategy (CIBSS, EXC-2189, project ID 390939984 to RZ).
- MOTIVATE program of the Medical Faculty, University of Freiburg (to AZ).
- Deutsche Krebshilfe (grants 70114655 to RZ and 70116490 to N Köhler).
- Deutsche José Carreras Leukämie-Stiftung (DJCLS 09R/2022; to RZ).
- Blood Cancer United, formerly The Leukemia & Lymphoma Society (7030-23 to RZ).
- EU project 101119855, exTra.
- Lighthouse Core Facility (Medical Faculty, University of Freiburg; 2021/A2-Fol and 2021/B3-Fol; DFG project 450392965).
- Ministry for Science, Research and Arts of the State of Baden-Wuerttemberg (to VH).
- Landesforschungspreis 2026 by the Ministry for Science, Research and Arts of the State of Baden-Wuerttemberg (to RZ).
- EU project 101119855 — extra.
- German Federal Ministry of Education and Research (BMBF) within the medical informatics funding scheme PM4Onco–FKZ 01ZZ2322A (to MB).
- EkoEstMed–FKZ 01ZZ2015 (to GA).

## Supplementary Material

Supplemental data

Supporting data values

## Figures and Tables

**Figure 1:**
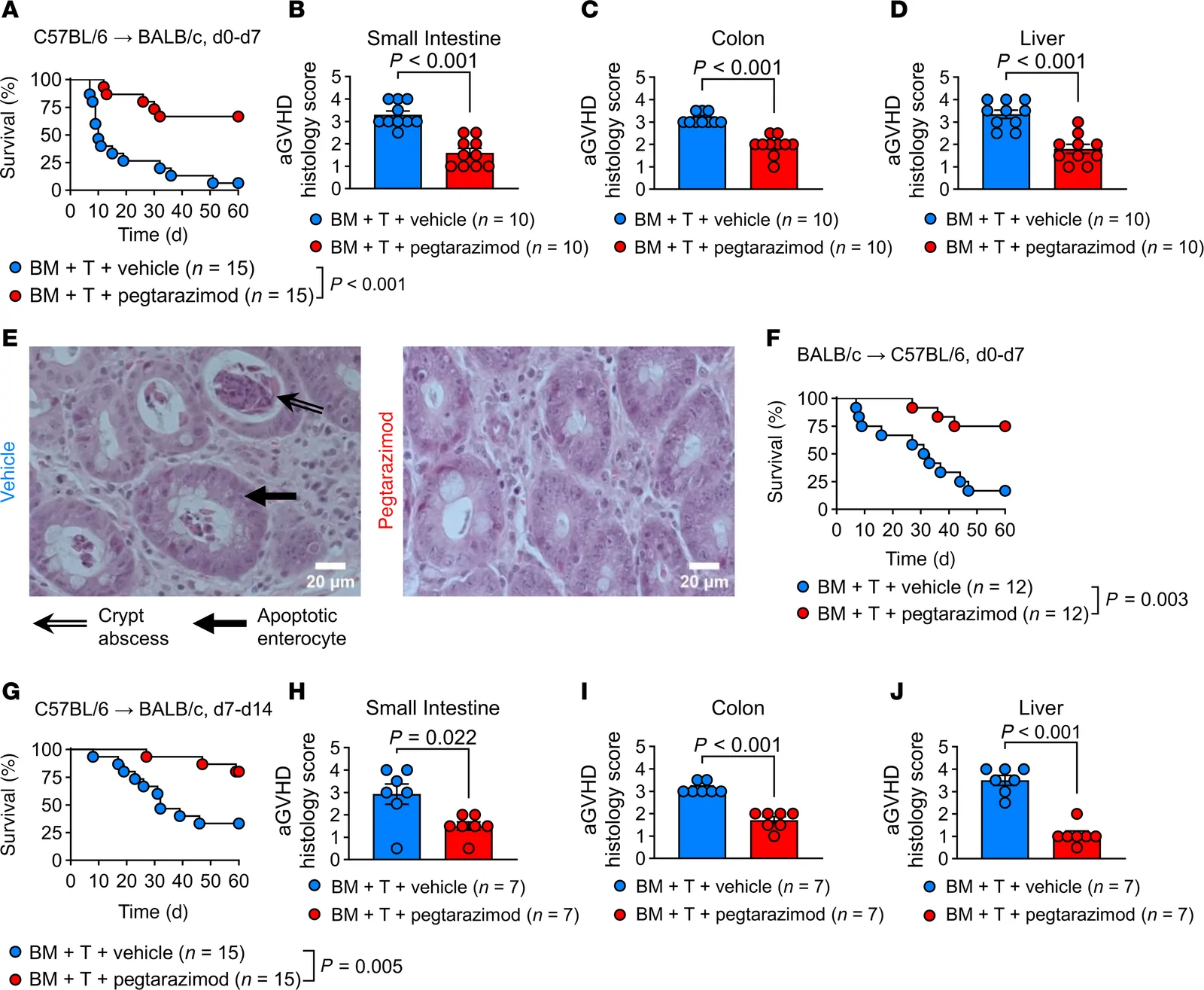
Pegtarazimod improves survival and histopathology in aGVHD. (**A**) Percentage of survival of BALB/c allo-HCT mice treated with pegtarazimod or vehicle from days 0 to 7 (prevention model) (*n =* 15). Results are derived from 3 independent experiments. *P* value was calculated using the log-rank test. (**B**–**D**) aGVHD histopathological scores of SI (**B**), colon (**C**), and liver (**D**) of BALB/c allo-HCT mice on day 7, treated with pegtarazimod or vehicle from days 0 to 7 (*n =* 10). *P* values were calculated using the Mann-Whitney test. (**E**) Representative images of the SI of BALB/c allo-HCT on day 7, treated with pegtarazimod or vehicle from days 0 to 7, stained with H&E. The double-lined arrow indicates a crypt abscess and the single-lined arrow an apoptotic enterocyte. Scale bars: 20 μm. (**F**) Percentage of survival of C57BL/6 allo-HCT mice treated with pegtarazimod or vehicle from days 0 to 7 (*n =* 12). Results are derived from 3 independent experiments. *P* value was calculated using the log-rank test. (**G**) Percentage of survival of BALB/c allo-HCT mice treated with pegtarazimod or vehicle from days 7 to 14 (early intervention model) (*n =* 15). Results are derived from 2 independent experiments. *P* value was calculated using the log-rank test. (**H**–**J**) aGVHD histopathological scores of SI (**H**), colon (**I**), and liver (**J**) of BALB/c allo-HCT mice on day 14, treated with pegtarazimod or vehicle from days 7 to 14 (*n* *=* 7). *P* values were calculated using the Mann-Whitney test.

**Figure 2:**
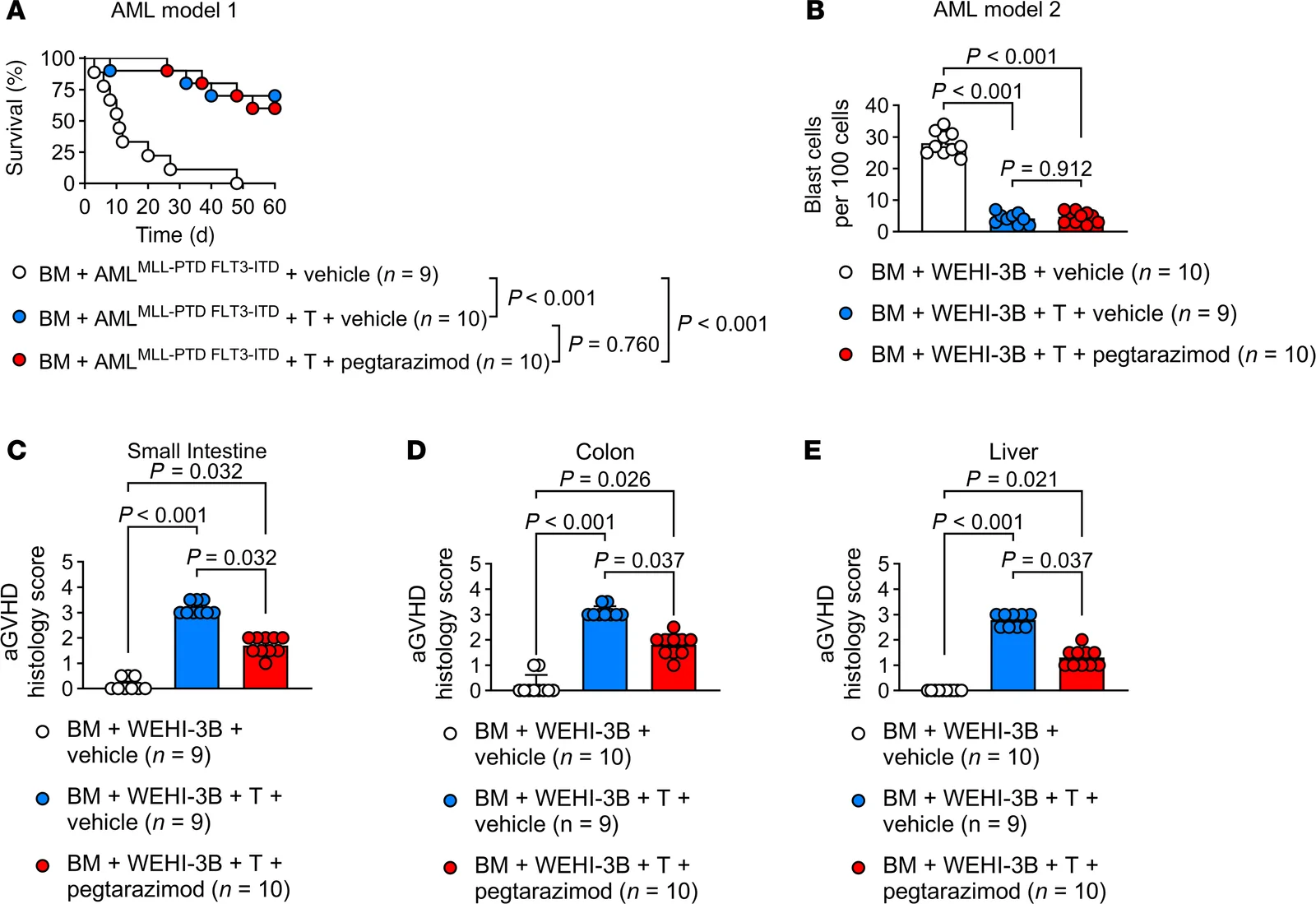
Pegtarazimod does not influence the antileukemia effect of allo-HCT. (**A**) Percentage of survival of C57BL/6 mice that received syngeneic AML^MLL-PTD^ ^FLT3-ITD^ cells and allo-HCT from BALB/c donor mice, treated with pegtarazimod or vehicle from days 0 to 7 (*n =* 9–10). Results are derived from 2 independent experiments. *P* values were calculated using the log-rank test. (**B**) Blast cells in the BM of BALB/c mice that received syngeneic WEHI-3B cells and allo-HCT from C57BL/6 donor mice, treated with pegtarazimod or vehicle from days 0 to 7 (*n* = 9–10). Results are derived from 2 independent experiments. *P* values were calculated using an ordinary 1-way ANOVA with Holm-Šidák multiple-comparison test. (**C**–**E**) aGVHD histopathological scores of SI (**C**), colon (**D**), and liver (**E**) of BALB/c mice that received syngeneic WEHI-3B cells and allo-HCT from C57BL/6 donor mice, treated with pegtarazimod or vehicle from days 0 to 7 (*n* = 9–10). Results are derived from 2 independent experiments. *P* values were calculated using Kruskal-Wallis test with Dunn’s multiple-comparison test.

**Figure 3:**
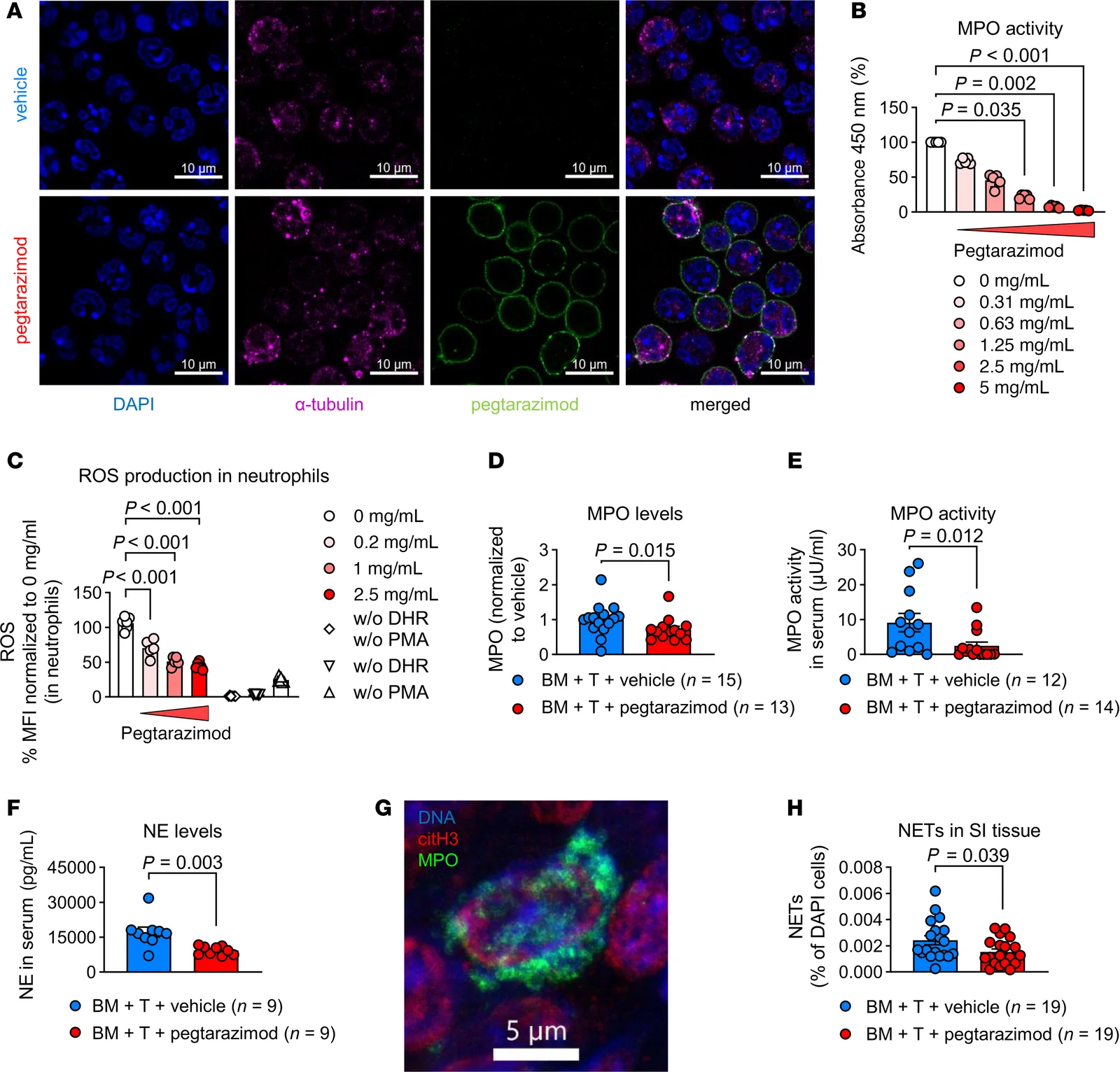
Pegtarazimod binds to neutrophils and decreases ROS production and NETosis in aGVHD. (**A**) Representative images showing IF staining for DAPI (blue), α-tubulin (pink), and pegtarazimod (green) of primary mouse neutrophils, treated with vehicle (upper panel) or pegtarazimod (lower panel) for 1 h. Scale bars: 10 μm. (**B**) Inhibition of MPO activity by pegtarazimod, assessed by absorbance measurement of TMB oxidation. Results are derived from 2 independent experiments. *P* values were calculated using the Kruskal-Wallis test with Dunn’s multiple-comparison test. (**C**) Inhibition of ROS production by pegtarazimod in neutrophils activated with PMA, measured by ROS-dependent conversion of DHR123 to fluorescent R123 using flow cytometry. Results are derived from 3 independent experiments. *P* values were calculated using an ordinary 1-way ANOVA with Holm-Šidák multiple-comparison test. (**D**–**F**) Analysis of MPO levels (**D**), MPO activity (**E**), and NE levels (**F**) in the serum of BALB/c allo-HCT mice on day 7, treated with pegtarazimod or vehicle from days 0 to 7. (**D**) MPO levels measured by ELISA (*n =* 13–15). *P* values were calculated using the Mann-Whitney test. (**E**) MPO activity measured using an MPO peroxidation activity assay kit (*n =* 12–14). *P* values were calculated using the Mann-Whitney test. (**F**) NE levels measured by ELISA (*n =* 9). *P* values were calculated using the Mann-Whitney test. (**G** and **H**) IF staining of NETosis. SI of BALB/c allo-HCT mice on day 7, treated with pegtarazimod or vehicle from days 0 to 7. (**G**) Representative image. IF staining for DAPI (blue), citrullinated histone H3 (red), and MPO (green). Scale bar: 5 μm. (**H**) Quantification of NETosis was performed using automated detection of cells with high signal for citrullinated histone H3 and granular MPO signal, divided by the number of single cells detected in the sample (*n =* 19). *P* values were calculated using an unpaired 2-tailed Student’s *t* test.

**Figure 4:**
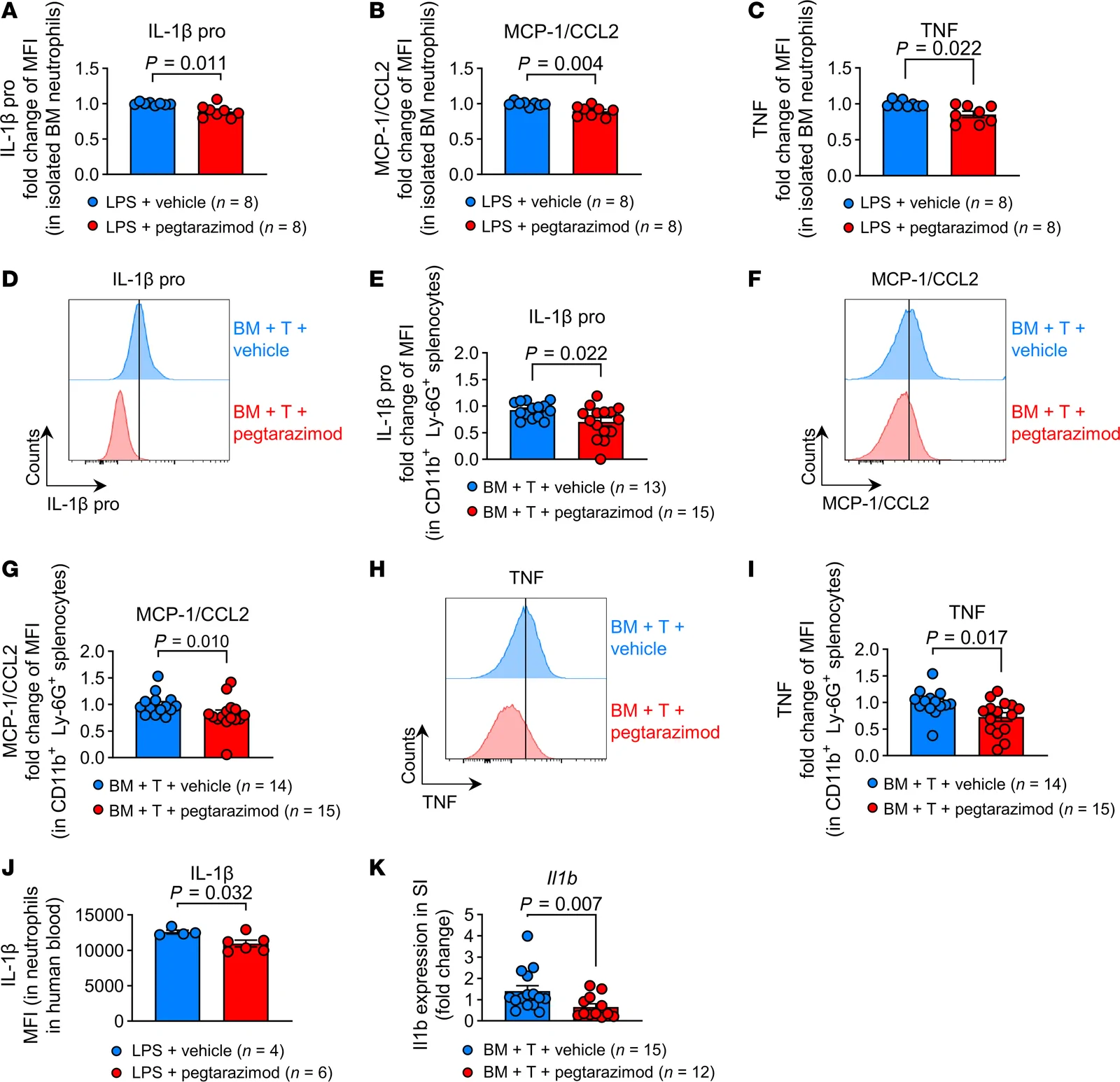
Pegtarazimod suppresses neutrophil inflammatory cytokine and chemokine production in vitro and in aGVHD in vivo. (**A**–**C**) Expression of IL-1β pro (**A**), MCP-1/CCL2 (**B**), and TNF (**C**) by neutrophils isolated from C57BL/6 BM challenged with LPS and treated with pegtarazimod or vehicle for 1 h, measured by flow cytometry (*n =* 8). *P* values were calculated using an unpaired 2-tailed Student’s *t* test with Welch’s correction for IL-1β pro and MCP-1/CCL2, and the Mann-Whitney test for TNF. (**D**–**I**) Representative histograms and quantification of IL-1β pro (**D** and **E**), MCP-1/CCL2 (**F** and** G**), and TNF (**H** and **I**) expression by spleen neutrophils of BALB/c allo-HCT mice on day 7, treated with pegtarazimod or vehicle from days 0 to 7, measured by flow cytometry (*n* *=* 13–15). *P* values were calculated using an unpaired 2-tailed Student’s *t* test with Welch’s correction for IL-1β pro and MCP-1/CCL2, and the Mann-Whitney test for TNF. (**J**) Expression of IL-1β by neutrophils in human blood challenged with LPS and treated with pegtarazimod or vehicle for 1 h, measured by flow cytometry (*n =* 4–6). *P* values were calculated using an unpaired 2-tailed Student’s *t* test. (**K**) Gene expression of *Il1b* in the SI of BALB/c allo-HCT mice on day 7, treated with pegtarazimod or vehicle from days 0 to 7 and measured by qPCR (*n =* 12–15). *P* values were calculated using a Mann-Whitney test.

**Figure 5:**
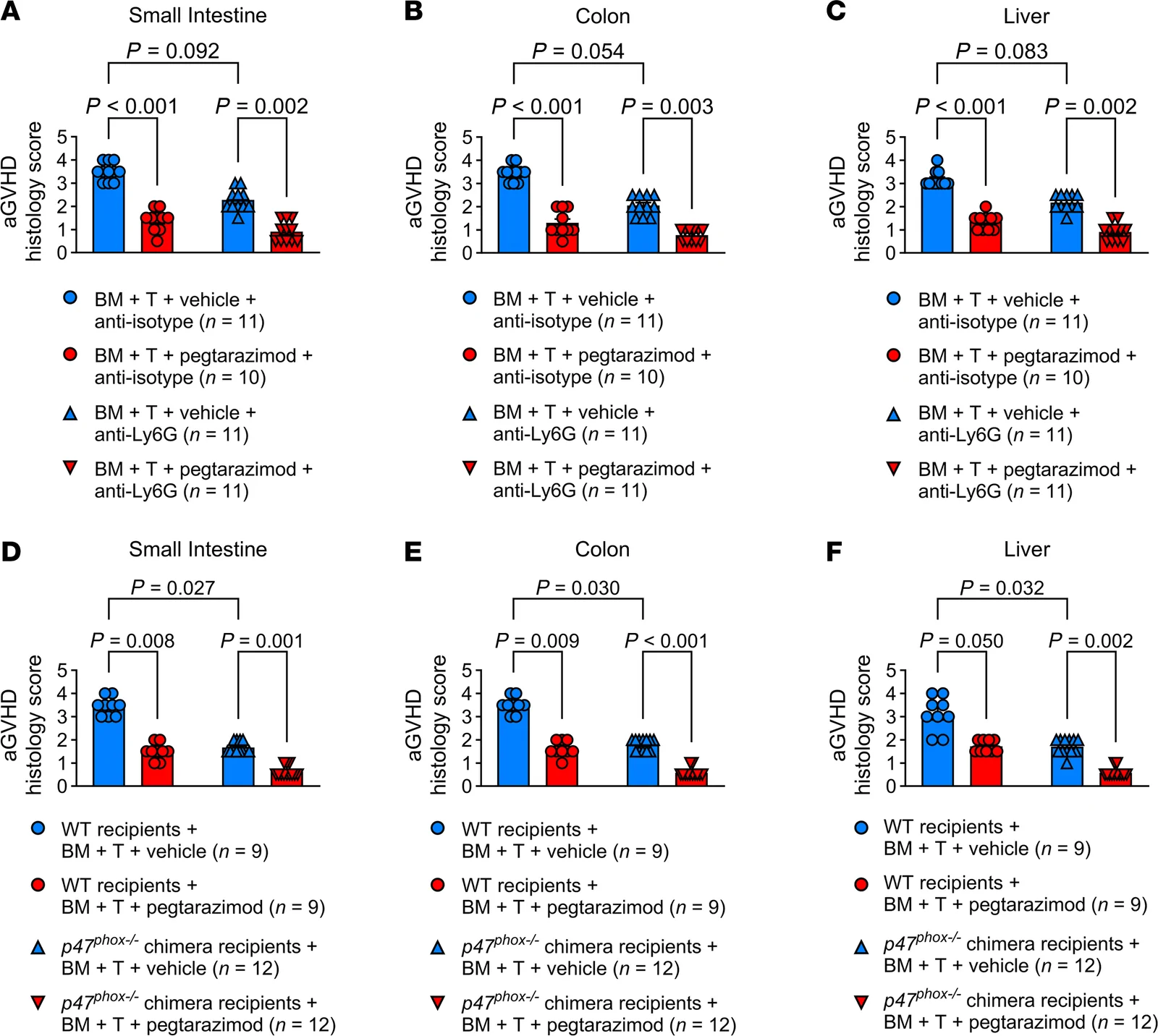
Pegtarazimod alleviates aGVHD histopathological damage independent of neutrophils and ROS. (**A**–**C**) aGVHD histopathological scores of neutrophil-depleted (anti-Ly6G) or control (anti-isotype) BALB/c allo-HCT mice on day 7, treated with pegtarazimod or vehicle from days 0 to 7 (*n* = 10–11). SI (**A**), colon (**B**), and liver (**C**). *P* values were calculated using Kruskal-Wallis test with Dunn’s multiple-comparison test. (**D**–**F**) aGVHD histopathological scores of control CD45.1 WT or ROS-deficient CD45.1-*p47^phox–/–^* chimera recipient mice on day 7 after allo-HCT with BALB/c BM and T cells, treated with pegtarazimod or vehicle from days 0 to 7 (*n* = 9–12). SI (**D**), colon (**E**), and liver (**F**). *P* values were calculated using Kruskal-Wallis test with Dunn’s multiple-comparison test.

**Figure 6:**
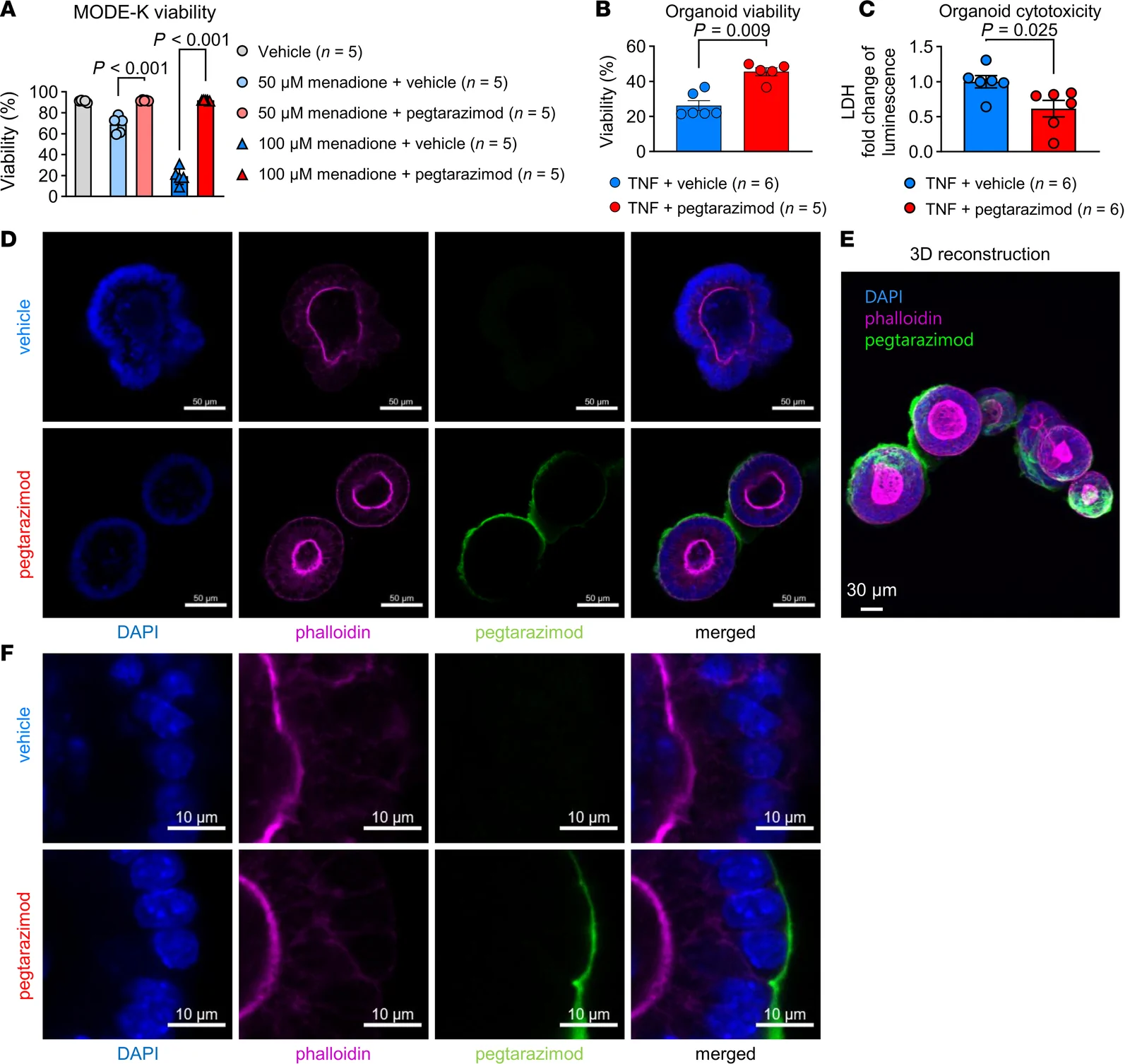
Pegtarazimod binds to enterocytes and has a protective effect. (**A**) Viability of MODE-K cells, challenged with menadione and treated with pegtarazimod or vehicle for 2 h, measured by flow cytometry (*n =* 6). Results are derived from 2 independent experiments. *P* values were calculated using an ordinary 1-way ANOVA with Holm-Šidák multiple-comparison test. (**B**) Viability of small intestinal organoids derived from BALB/c mice, challenged with TNF, treated with pegtarazimod or vehicle for 2 days, and measured by flow cytometry (*n =* 5–6). *P* values were calculated using the Mann-Whitney test. (**C**) LDH release of small intestinal organoids derived from BALB/c mice, challenged with TNF, treated with pegtarazimod or vehicle for 2 days, and measured using the LDH-Glo Cytotoxicity Assay (*n =* 6). *P* values were calculated using an unpaired 2-tailed Student’s *t* test. (**D**–**F**) IF staining for DAPI (blue), phalloidin (pink), and pegtarazimod (green) of small intestinal organoids, treated with pegtarazimod or vehicle for 24 h. (**D**) Representative overview images. Scale bars: 50 μm. (**E**) 3D reconstruction of overview images using IMARIS (10.2). Scale bar: 30 μm. (**F**) Representative detailed images. Scale bars: 10 μm.

**Figure 7:**
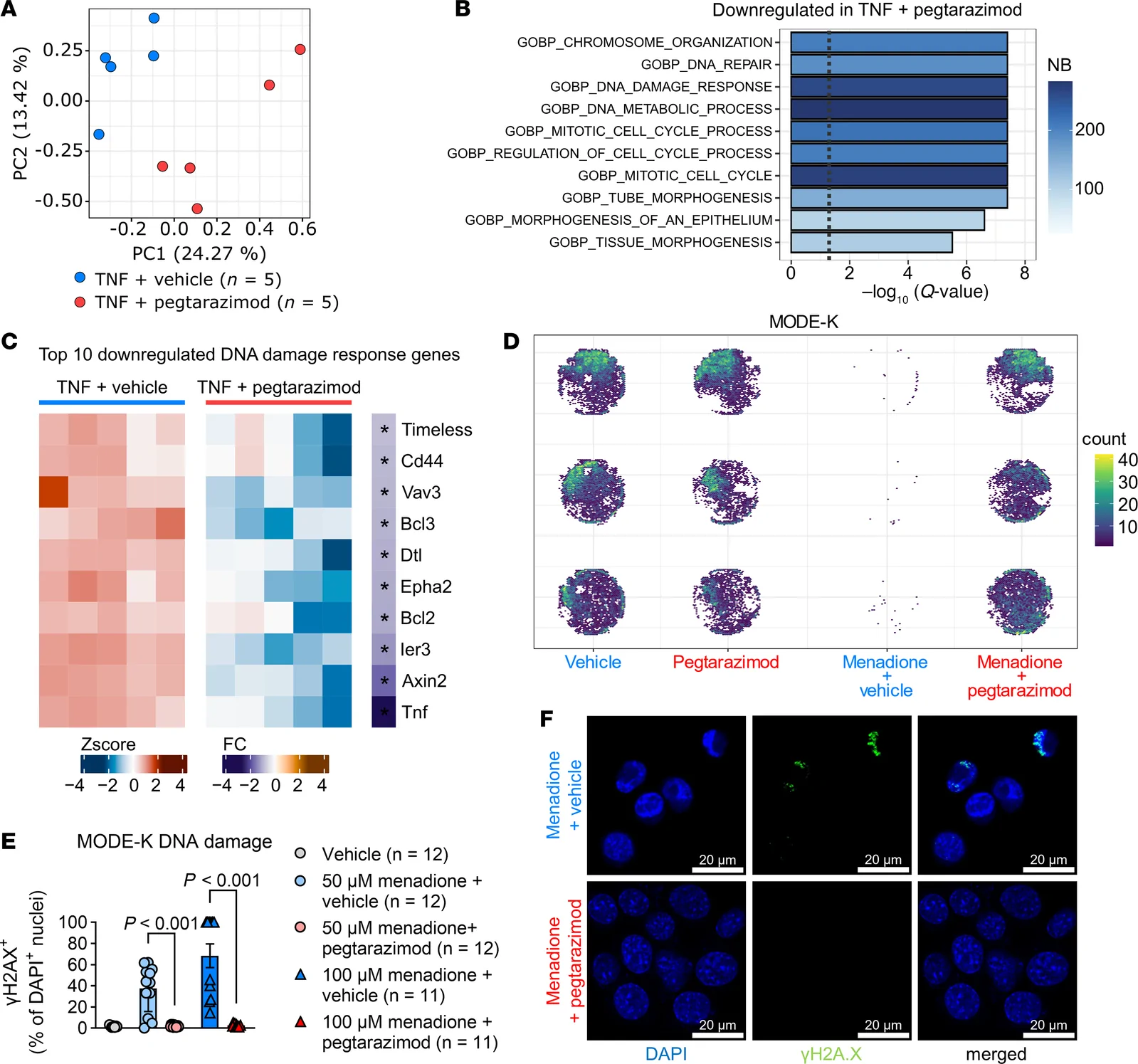
Pegtarazimod protects enterocytes by decreasing DNA damage. (**A**–**C**) Gene expression profiling of small intestinal organoids derived from BALB/c mice, challenged with TNF and treated with pegtarazimod or vehicle for 2 days (*n* = 5). (**A**) PCA based on log_2_ robust multiarray average normalized intensity of all genes. (**B**) Top 10 most downregulated [ranked by –log_10_(*q* value)] Gene Ontology Biological Process (GOBP) gene sets in the TNF + pegtarazimod group compared with the TNF + vehicle group. Bar color intensity represents the number of genes (NB) in the leading edge for each term (*n* = 5). (**C**) Heatmap showing the top 10 most downregulated genes of the GOBP term DNA damage response in the TNF + pegtarazimod group compared with the TNF + vehicle group. Colors represent row-wise *z* scores (left) and log_2_ fold changes (FC; right). Genes marked with an asterisk are significantly differentially expressed (adjusted *P* < 0.05). (**D**–**F**) MODE-K cells were challenged with menadione and treated with pegtarazimod or vehicle for 2 h, and expression of γH2A.X was assessed using IF staining of cells in a 96-well plate. (**D**) Schematic representation of cell density in the wells. Color gradient indicates relative cell counts (see scale). (**E**) Quantification of MODE-K cells expressing γH2A.X relative to the number of cells in the well (*n* = 11–12). *P* values were calculated using Kruskal-Wallis test with Dunn’s multiple-comparison test. (**F**) Representative images of IF staining for DAPI (blue) and γH2A.X (green). Scale bars: 20 μm.

**Figure 8:**
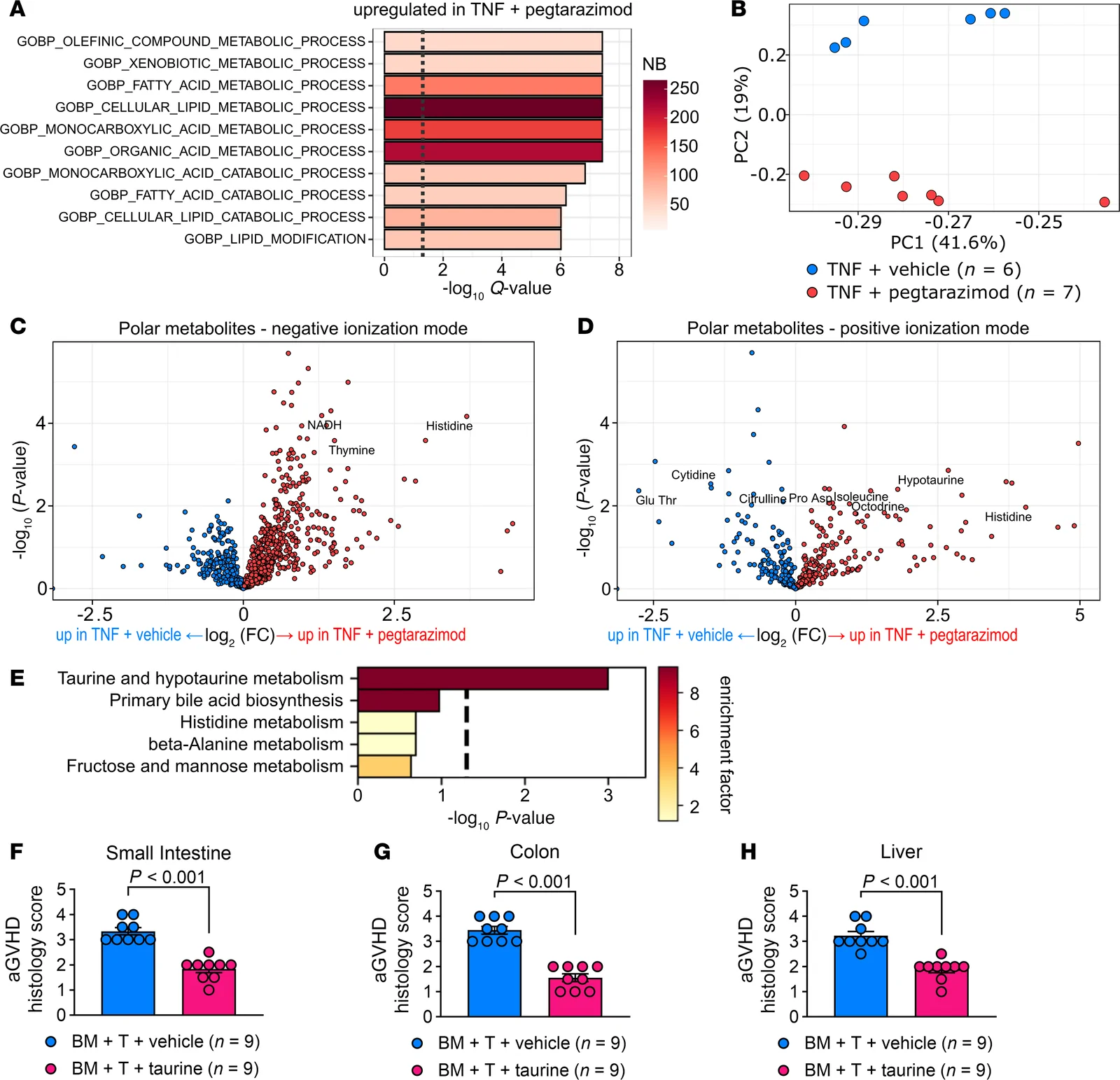
Pegtarazimod modulates the metabolism of small intestinal organoids. (**A**) Top 10 most upregulated GOBP gene sets [ranked by –log_10_(*q* value)] in small intestinal organoids derived from BALB/c mice, challenged with TNF and treated with pegtarazimod for 2 days, compared with organoids challenged with TNF and treated with vehicle for 2 days. Bar color intensity represents the number of genes (NB) in the leading edge for each term (*n* = 5). (**B**–**E**) LC-MS metabolite analysis of small intestinal organoids derived from BALB/c mice, challenged with TNF and treated with pegtarazimod or vehicle for 2 days (*n* = 6–7). (**B**) PCA of polar metabolites and lipids. (**C**) Differential abundance of polar metabolites measured with negative ionization. (**D**) Differential abundance of polar metabolites measured with positive ionization. (**E**) Pathway analysis using Mummichog. Shown are top 5 hits ranked by log_10_(*P* value). Enrichment factor indicates overrepresentation of metabolic pathways between the groups. (**F**–**H**) aGVHD histopathological scores of SI (**F**), colon (**G**), and liver (**H**) of BALB/c allo-HCT mice on day 7, treated with 200 mM taurine or vehicle from days –7 to 7 (*n =* 9). Results are derived from 2 independent experiments. *P* values were calculated using the Mann-Whitney test.

**Figure 9:**
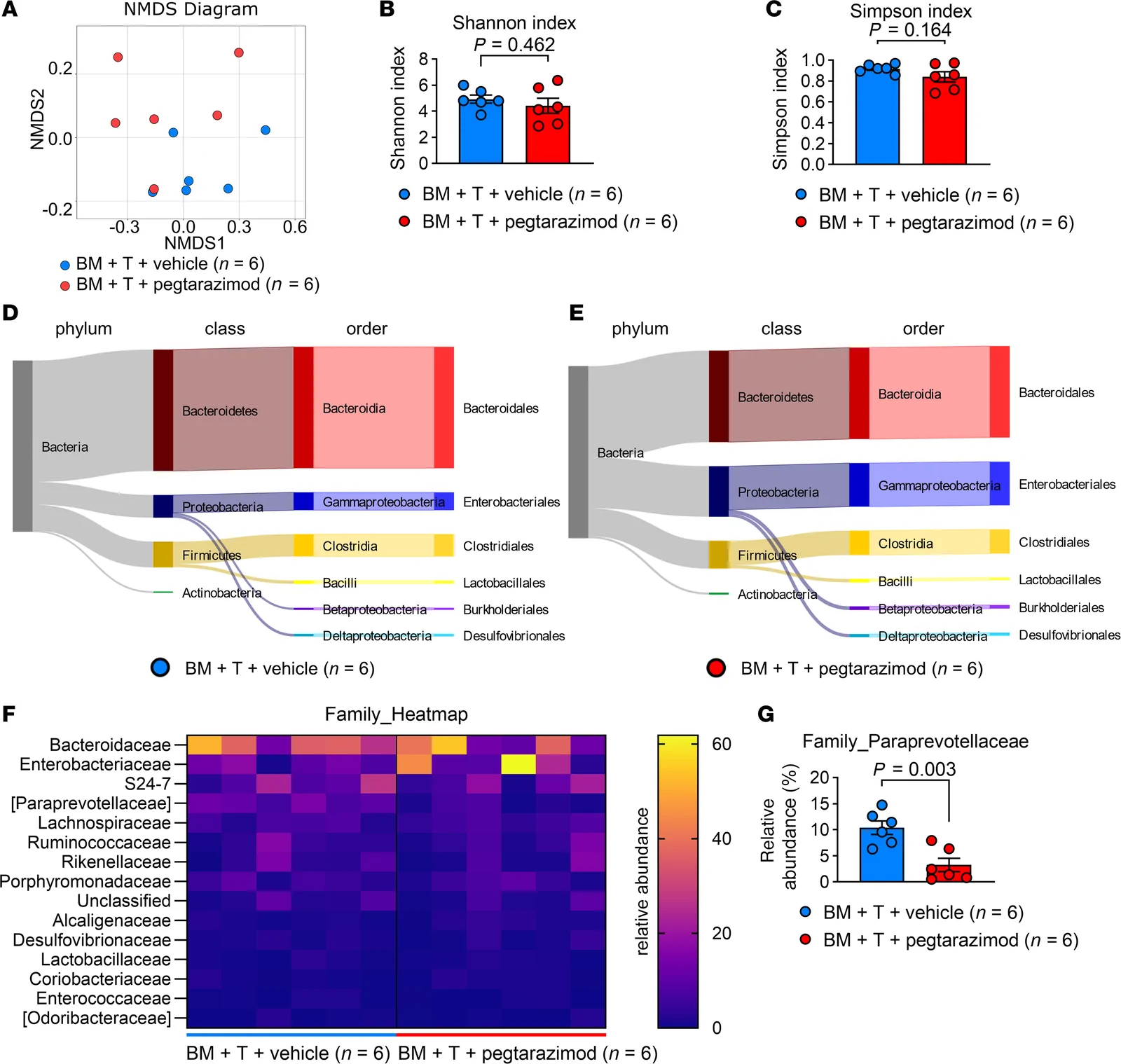
Pegtarazimod has minor effects on the intestinal microbiome of mice with aGVHD. (**A**–**G**) 16S rDNA sequencing of stool of BALB/c allo-HCT mice on day 7, treated with pegtarazimod or vehicle from days 0 to 7 (*n =* 6). (**A**) NMDS diagram showing differences in beta diversity. (**B**) Alpha diversity, measured by Shannon index. *P* values were calculated using an unpaired 2-tailed Student’s *t* test. (**C**) Alpha diversity, measured by Simpson index. *P* values were calculated using an unpaired 2-tailed Student’s *t* test. (**D**) Sankey diagram of phyla, classes, and orders of bacteria in the vehicle-treated group. (**E**) Sankey diagram of phyla, classes, and orders of bacteria in the pegtarazimod-treated group. (**F**) Heatmap of relative abundances of bacterial families. (**G**) Relative abundance of the family Paraprevotellaceae. *P* values were calculated using an unpaired 2-tailed Student’s *t* test.

**Figure 10:**
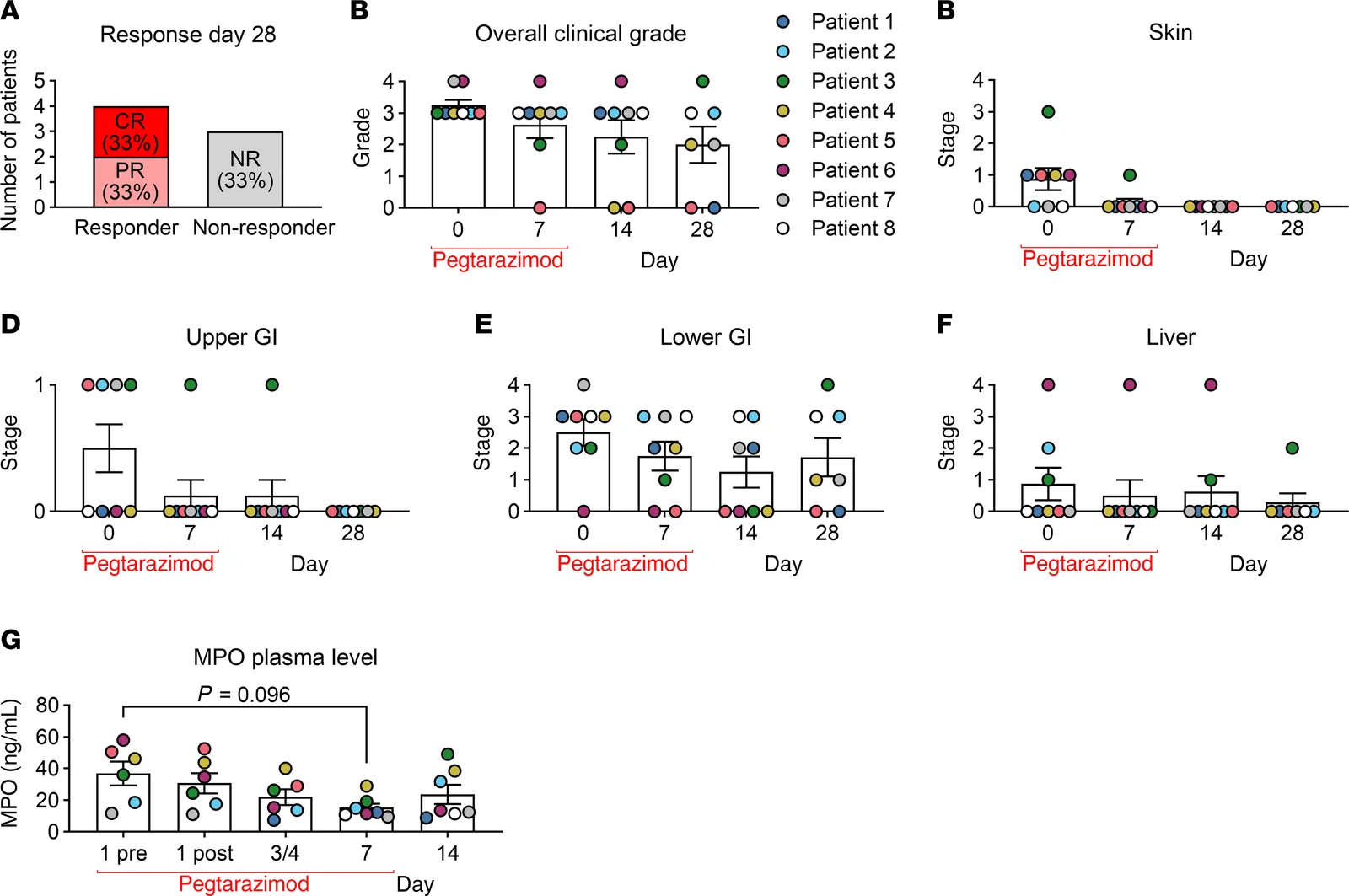
Clinical trial (AURORA) of pegtarazimod for patients with SR-aGVHD. (**A**–**G**) Results of SR-aGVHD patients in the AURORA trial, treated with 10 mg/kg pegtarazimod for 7 days. (**A**) Response to treatment on day 28. Shown are complete response (CR), partial response (PR), and no response (NR) of evaluable patients. (**B**) Overall clinical grades. (**C**–**F**) aGVHD staging of the skin (**C**), upper GI (**D**), lower GI (**E**), and liver (**F**). (**G**) Plasma levels of MPO. *P* values were calculated using a paired 2-tailed *t* test.

## Version 1 08/04/2026

In-Press Preview

## Version 2 10/01/2026

Electronic publication
