# Supplementary material for: Pegtarazimod limits acute graft-versus-host disease mortality and severity in mice and is tolerated in patients
Source: J Clin Invest. 2026 Aug 4;136(19):e205864. doi: 10.1172/JCI205864 (PMC13626831; doi:10.1172/JCI205864)
Supplement: Supplementary file 1 [file jci-136-205864-s099.pdf]

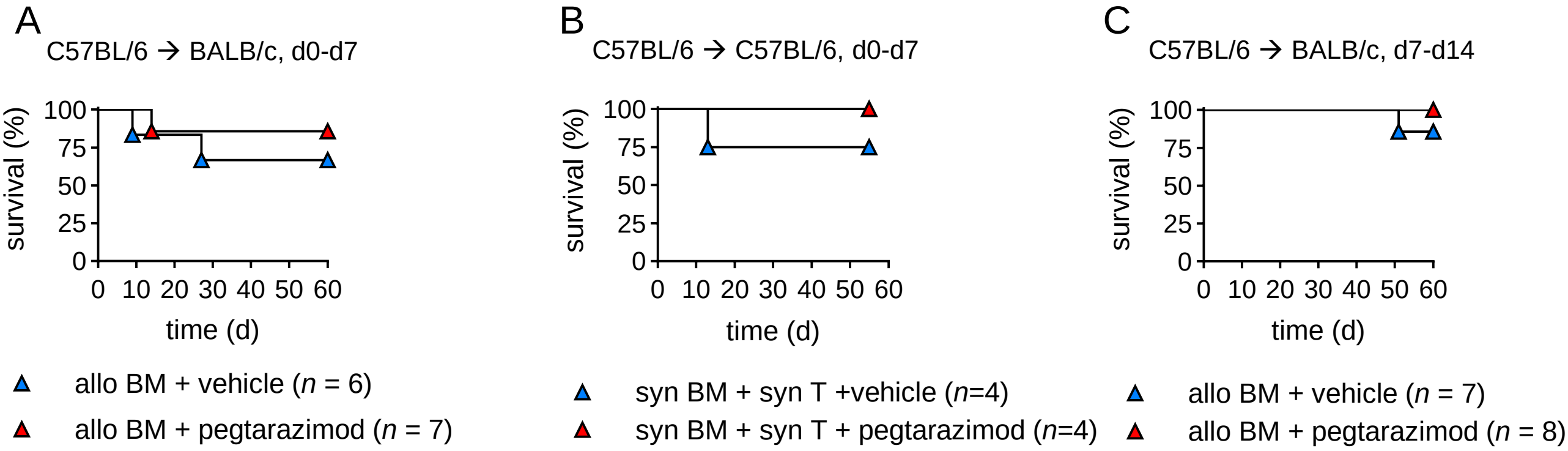

A
C57BL/6 → BALB/c, d0-d7
survival (%)
100
75
50
25
0
0 10 20 30 40 50 60
time (d)
allo BM + vehicle (n = 6)
allo BM + pegtarazimod (n = 7)
B
C57BL/6 → C57BL/6, d0-d7
survival (%)
100
75
50
25
0
0 10 20 30 40 50 60
time (d)
syn BM + syn T +vehicle (n=4)
syn BM + syn T + pegtarazimod (n=4)
C
C57BL/6 → BALB/c, d7-d14
survival (%)
100
75
50
25
0
0 10 20 30 40 50 60
time (d)
allo BM + vehicle (n = 7)
allo BM + pegtarazimod (n = 8)

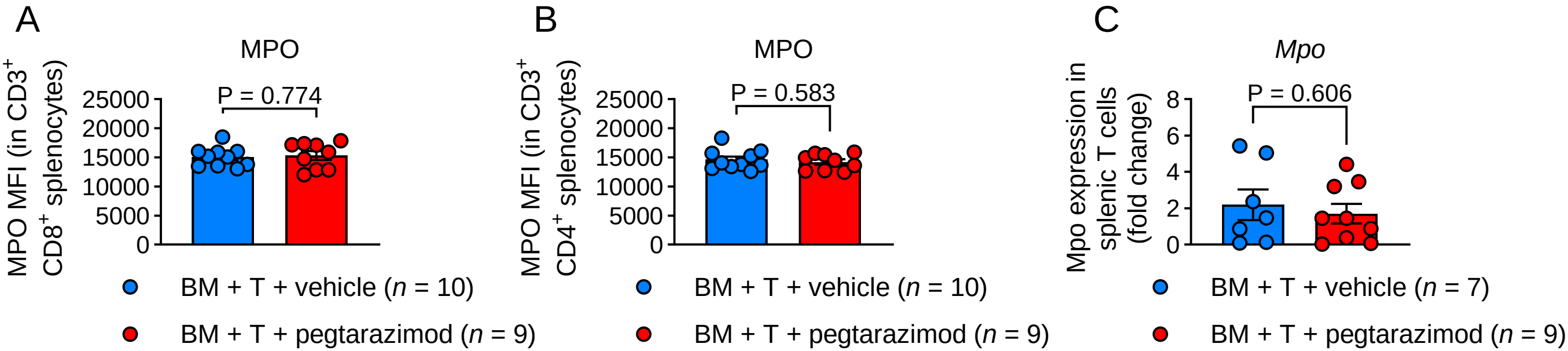
A
MPO
MPO MFI (in CD3+ CD8+ splenocytes)
25000
20000
15000
10000
5000
0
P = 0.774
B
MPO
MPO MFI (in CD3+ CD4+ splenocytes)
25000
20000
15000
10000
5000
0
P = 0.583
C
Mpo
Mpo expression in splenic T cells (fold change)
8
6
4
2
0
P = 0.606
BM + T + vehicle (n = 10)
BM + T + pegtarazimod (n = 9)
BM + T + vehicle (n = 10)
BM + T + pegtarazimod (n = 9)
BM + T + vehicle (n = 7)
BM + T + pegtarazimod (n = 9)

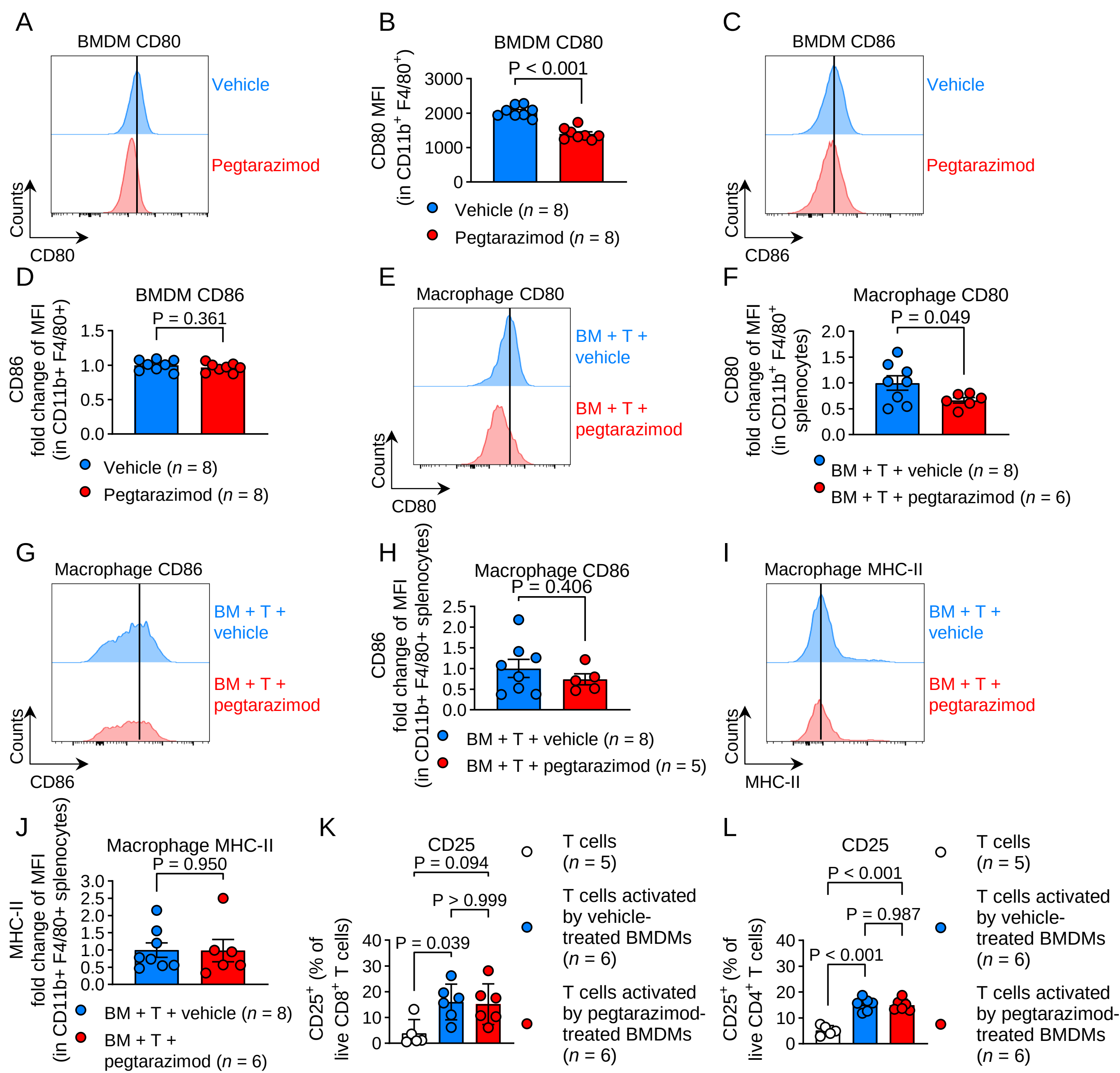
A
BMDM CD80
Vehicle
Pegtarazimod
Counts
CD80
B
BMDM CD80
P < 0.001
CD80 MFI (in CD11b+ F4/80+)
3000
2000
1000
0
Vehicle (n = 8)
Pegtarazimod (n = 8)
C
BMDM CD86
Vehicle
Pegtarazimod
Counts
CD86
D
BMDM CD86
P = 0.361
CD86 fold change of MFI (in CD11b+ F4/80+)
1.5
1.0
0.5
0.0
Vehicle (n = 8)
Pegtarazimod (n = 8)
E
Macrophage CD80
BM + T + vehicle
BM + T + pegtarazimod
Counts
CD80
F
Macrophage CD80
P = 0.049
CD80 fold change of MFI (in CD11b+ F4/80+ splenocytes)
2.0
1.5
1.0
0.5
0.0
BM + T + vehicle (n = 8)
BM + T + pegtarazimod (n = 6)
G
Macrophage CD86
BM + T + vehicle
BM + T + pegtarazimod
Counts
CD86
H
Macrophage CD86
P = 0.406
CD86 fold change of MFI (in CD11b+ F4/80+ splenocytes)
2.5
2.0
1.5
1.0
0.5
0.0
BM + T + vehicle (n = 8)
BM + T + pegtarazimod (n = 5)
I
Macrophage MHC-II
BM + T + vehicle
BM + T + pegtarazimod
Counts
MHC-II
J
Macrophage MHC-II
P = 0.950
MHC-II fold change of MFI (in CD11b+ F4/80+ splenocytes)
3.0
2.5
2.0
1.5
1.0
0.5
0.0
BM + T + vehicle (n = 8)
BM + T + pegtarazimod (n = 6)
K
CD25
P = 0.094
P > 0.999
P = 0.039
CD25+ (% of live CD8+ T cells)
40
30
20
10
0
T cells (n = 5)
T cells activated by vehicle-treated BMDMs (n = 6)
T cells activated by pegtarazimod-treated BMDMs (n = 6)
L
CD25
P < 0.001
P = 0.987
P < 0.001
CD25+ (% of live CD4+ T cells)
40
30
20
10
0
T cells (n = 5)
T cells activated by vehicle-treated BMDMs (n = 6)
T cells activated by pegtarazimod-treated BMDMs (n = 6)

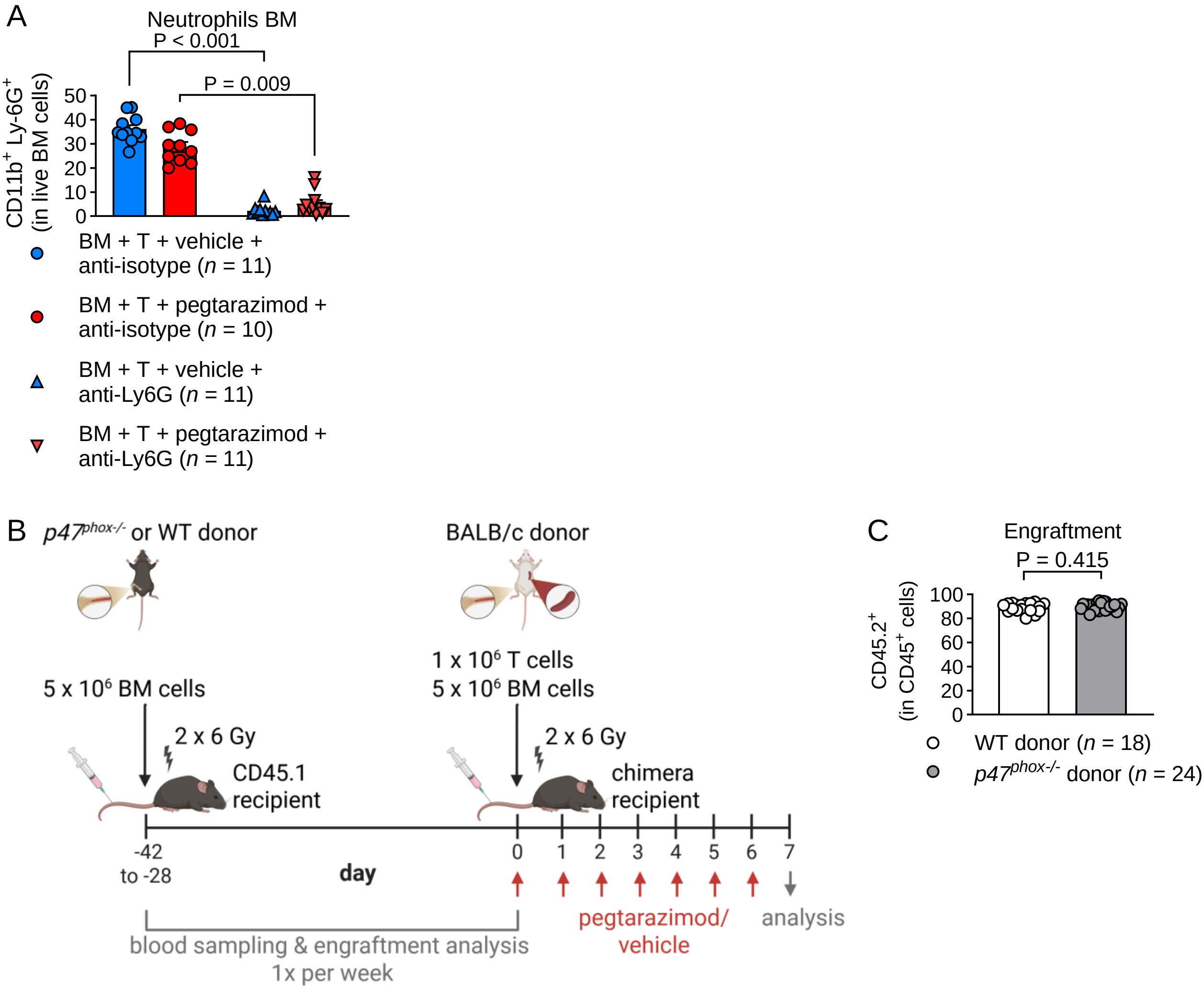
A
Neutrophils BM
P < 0.001
P = 0.009
CD11b+ Ly-6G+ (in live BM cells)
50
40
30
20
10
0
BM + T + vehicle + anti-isotype (n = 11)
BM + T + pegtarazimod + anti-isotype (n = 10)
BM + T + vehicle + anti-Ly6G (n = 11)
BM + T + pegtarazimod + anti-Ly6G (n = 11)
B
p47phox-/- or WT donor
BALB/c donor
5 x 10⁶ BM cells
1 x 10⁶ T cells
5 x 10⁶ BM cells
2 x 6 Gy
CD45.1 recipient
2 x 6 Gy
chimera recipient
-42 to -28
day
0
1
2
3
4
5
6
7
blood sampling & engraftment analysis 1x per week
pegtarazimod/ vehicle
analysis
C
Engraftment
P = 0.415
CD45.2+ (in CD45+ cells)
100
80
60
40
20
0
WT donor (n = 18)
p47phox-/- donor (n = 24)

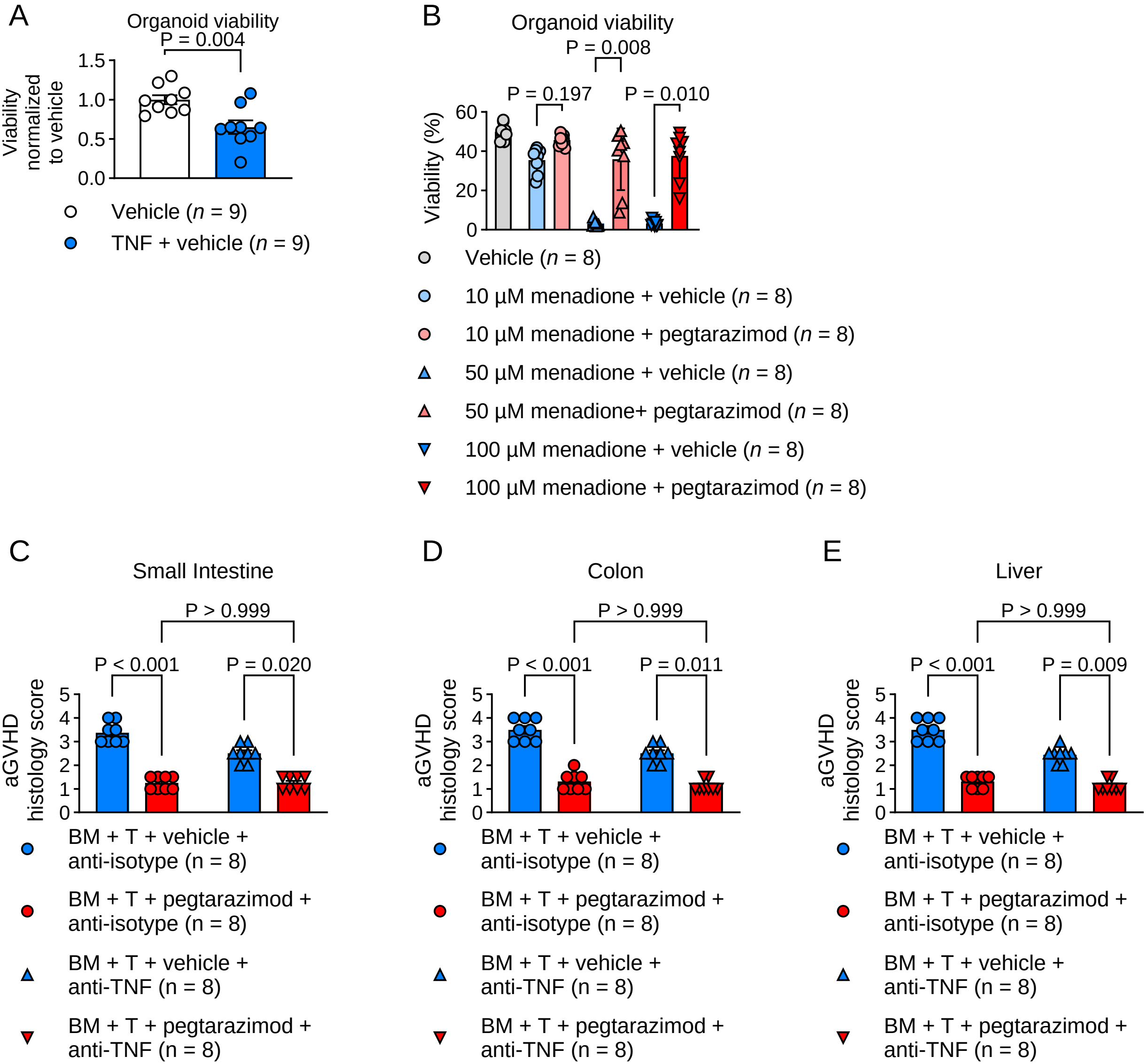
A
Organoid viability
P = 0.004
Viability normalized to vehicle
1.5
1.0
0.5
0.0
Vehicle (n = 9)
TNF + vehicle (n = 9)
B
Organoid viability
P = 0.008
P = 0.197
P = 0.010
Viability (%)
60
40
20
0
Vehicle (n = 8)
10 µM menadione + vehicle (n = 8)
10 µM menadione + pegtarazimod (n = 8)
50 µM menadione + vehicle (n = 8)
50 µM menadione+ pegtarazimod (n = 8)
100 µM menadione + vehicle (n = 8)
100 µM menadione + pegtarazimod (n = 8)
C
Small Intestine
P > 0.999
P < 0.001
P = 0.020
aGVHD histology score
5
4
3
2
1
0
BM + T + vehicle + anti-isotype (n = 8)
BM + T + pegtarazimod + anti-isotype (n = 8)
BM + T + vehicle + anti-TNF (n = 8)
BM + T + pegtarazimod + anti-TNF (n = 8)
D
Colon
P > 0.999
P < 0.001
P = 0.011
aGVHD histology score
5
4
3
2
1
0
BM + T + vehicle + anti-isotype (n = 8)
BM + T + pegtarazimod + anti-isotype (n = 8)
BM + T + vehicle + anti-TNF (n = 8)
BM + T + pegtarazimod + anti-TNF (n = 8)
E
Liver
P > 0.999
P < 0.001
P = 0.009
aGVHD histology score
5
4
3
2
1
0
BM + T + vehicle + anti-isotype (n = 8)
BM + T + pegtarazimod + anti-isotype (n = 8)
BM + T + vehicle + anti-TNF (n = 8)
BM + T + pegtarazimod + anti-TNF (n = 8)

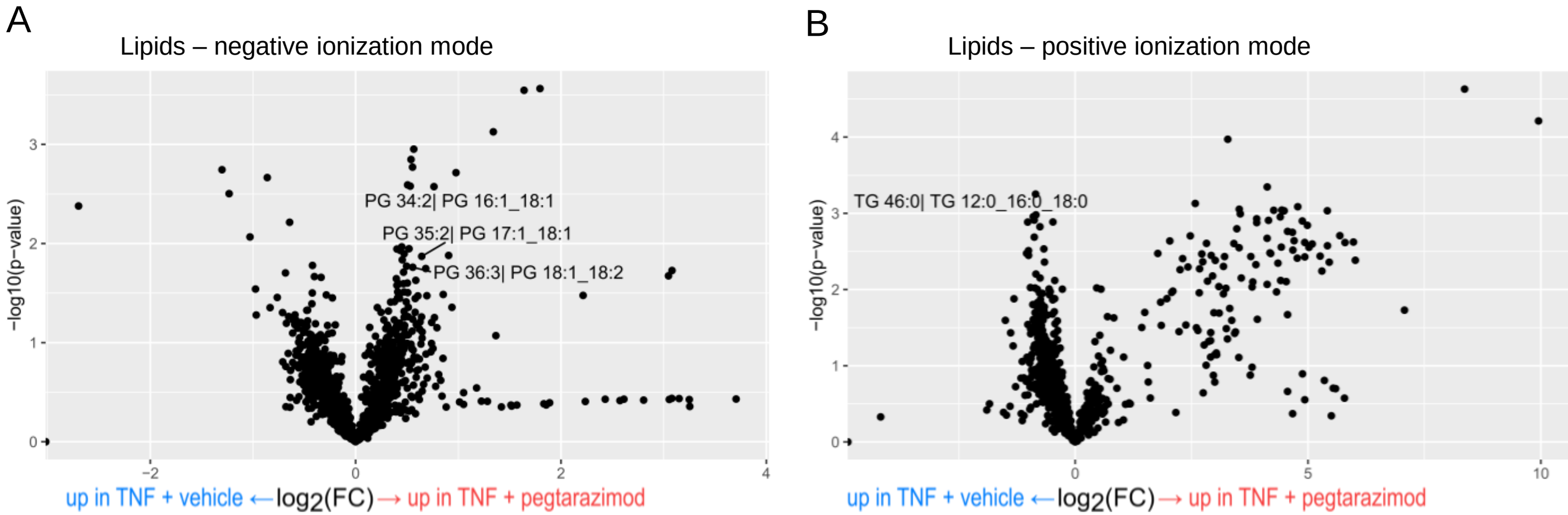
A
Lipids – negative ionization mode
PG 34:2| PG 16:1_18:1
PG 35:2| PG 17:1_18:1
PG 36:3| PG 18:1_18:2
−log10(p−value)
up in TNF + vehicle ← log2(FC) → up in TNF + pegtarazimod
B
Lipids – positive ionization mode
TG 46:0| TG 12:0_16:0_18:0
−log10(p−value)
up in TNF + vehicle ← log2(FC) → up in TNF + pegtarazimod

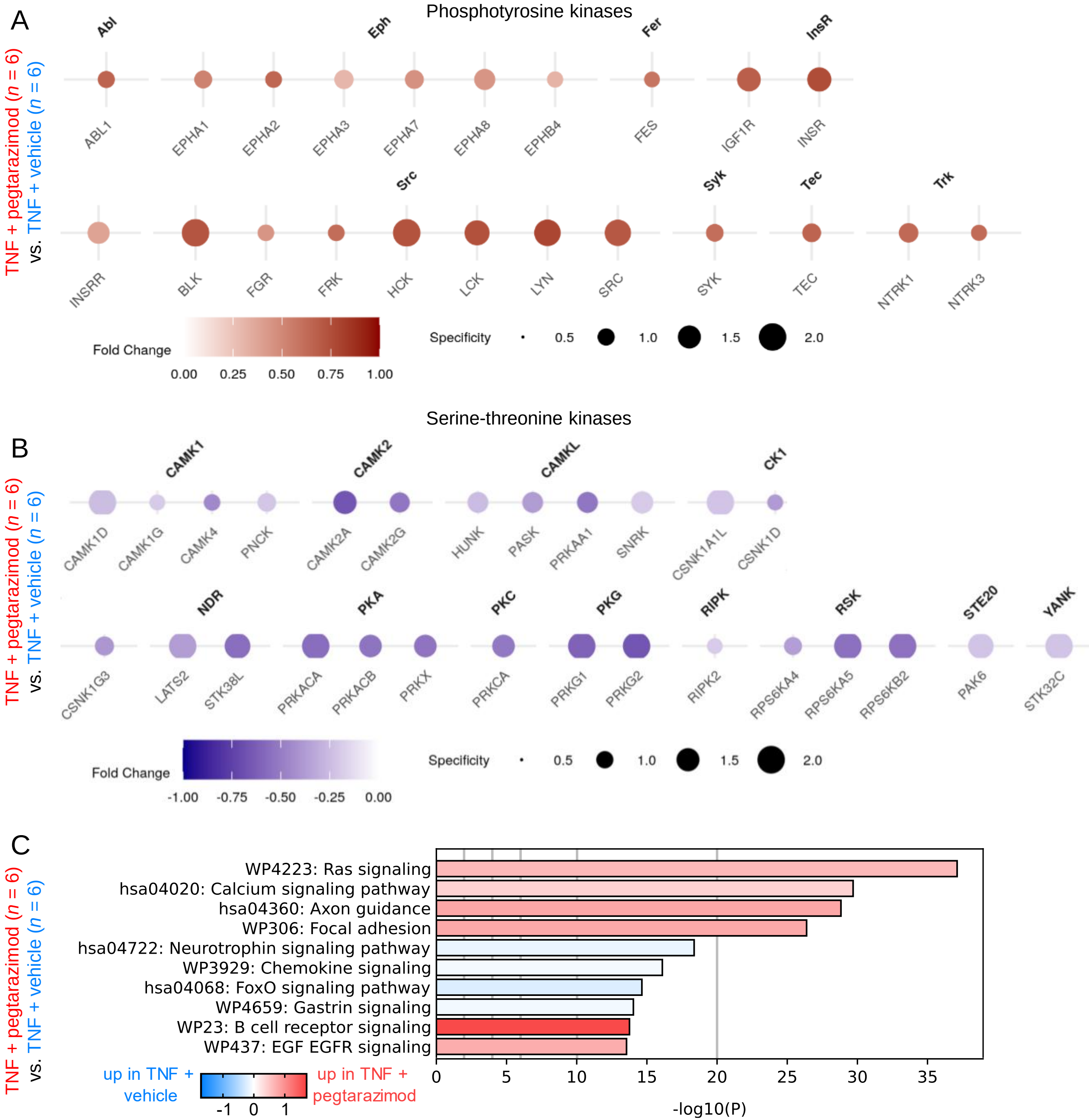
A
Phosphotyrosine kinases
TNF + pegtarazimod (n = 6) vs. TNF + vehicle (n = 6)
Abl
Eph
Fer
InsR
ABL1
EPHA1
EPHA2
EPHA3
EPHA7
EPHA8
EPHB4
FES
IGF1R
INSR
Src
Syk
Tec
Trk
INSRR
BLK
FGR
FRK
HCK
LCK
LYN
SRC
SYK
TEC
NTRK1
NTRK3
Fold Change
0.00 0.25 0.50 0.75 1.00
Specificity 0.5 1.0 1.5 2.0
B
Serine-threonine kinases
TNF + pegtarazimod (n = 6) vs. TNF + vehicle (n = 6)
CAMK1
CAMK2
CAMKL
CK1
CAMK1D
CAMK1G
CAMK4
PNCK
CAMK2A
CAMK2G
HUNK
PASK
PRKAA1
SNRK
CSNK1A1L
CSNK1D
NDR
PKA
PKC
PKG
RIPK
RSK
STE20
YANK
CSNK1G3
LATS2
STK38L
PRKACA
PRKACB
PRKX
PRKCA
PRKG1
PRKG2
RIPK2
RPS6KA4
RPS6KA5
RPS6KB2
PAK6
STK32C
Fold Change
-1.00 -0.75 -0.50 -0.25 0.00
Specificity 0.5 1.0 1.5 2.0
C
TNF + pegtarazimod (n = 6) vs. TNF + vehicle (n = 6)
WP4223: Ras signaling
hsa04020: Calcium signaling pathway
hsa04360: Axon guidance
WP306: Focal adhesion
hsa04722: Neurotrophin signaling pathway
WP3929: Chemokine signaling
hsa04068: FoxO signaling pathway
WP4659: Gastrin signaling
WP23: B cell receptor signaling
WP437: EGF EGFR signaling
0 5 10 15 20 25 30 35
-log10(P)
up in TNF + vehicle
-1 0 1
up in TNF + pegtarazimod

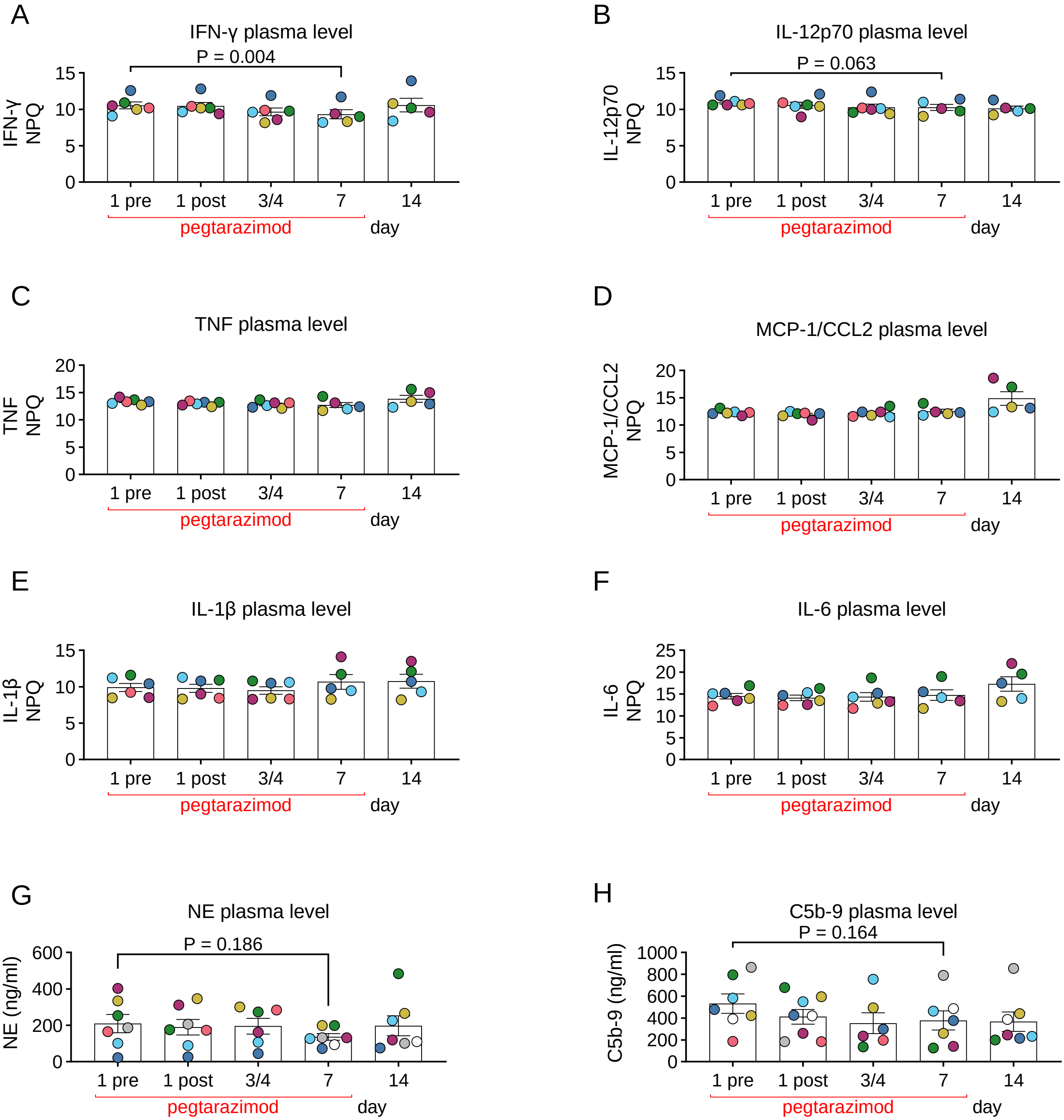

A
IFN-γ plasma level
P = 0.004
IFN-γ NPQ
0
5
10
15
1 pre
1 post
3/4
7
14
pegtarazimod
day
B
IL-12p70 plasma level
P = 0.063
IL-12p70 NPQ
0
5
10
15
1 pre
1 post
3/4
7
14
pegtarazimod
day
C
TNF plasma level
TNF NPQ
0
5
10
15
20
1 pre
1 post
3/4
7
14
pegtarazimod
day
D
MCP-1/CCL2 plasma level
MCP-1/CCL2 NPQ
0
5
10
15
20
1 pre
1 post
3/4
7
14
pegtarazimod
day
E
IL-1β plasma level
IL-1β NPQ
0
5
10
15
1 pre
1 post
3/4
7
14
pegtarazimod
day
F
IL-6 plasma level
IL-6 NPQ
0
5
10
15
20
25
1 pre
1 post
3/4
7
14
pegtarazimod
day
G
NE plasma level
P = 0.186
NE (ng/ml)
0
200
400
600
1 pre
1 post
3/4
7
14
pegtarazimod
day
H
C5b-9 plasma level
P = 0.164
C5b-9 (ng/ml)
0
200
400
600
800
1000
1 pre
1 post
3/4
7
14
pegtarazimod
day

Supplemental material

Supplemental Figure legends

Supplemental Figure 1: Additional experimental groups for aGVHD models.

(**A**) Percentage survival of BALB/c mice that received allogeneic bone marrow without additional T cells, treated with pegtarazimod or vehicle from day 0 to 7 (prevention model). (**B**) Percentage survival of C57BL/6 syn-HCT mice treated with pegtarazimod or vehicle from day 0 to 7. (**C**) Percentage survival of BALB/c mice that received allogeneic bone marrow without additional T cells, treated with pegtarazimod or vehicle from day 7 to 14 (early intervention model).

Supplemental Figure 2: MPO expression in T cells.

(**A** and **B**) Expression of MPO in $CD3^+$ $CD8^+$ (**A**) and $CD3^+$ $CD4^+$ (**B**) spleen T cells of BALB/c allo-HCT mice on day 7, treated with pegtarazimod or vehicle from day 0 to 7, measured by flow cytometry (n = 9-10). P-values were calculated using an unpaired two-tailed Student's *t*-test. (**C**) Gene expression of *Mpo* in isolated spleen T cells of BALB/c allo-HCT mice on day 7, treated with pegtarazimod or vehicle from day 0 to 7, measured by qPCR. P-values were calculated using a Mann-Whitney test.

Supplemental Figure 3: Influence of pegtarazimod on macrophages in vitro and in aGVHD in vivo.

(**A**-**D**) Representative histogram and quantification of CD80 (**A** and **B**), and CD86 (**C** and **D**) expression in BMDM treated with pegtarazimod or vehicle for 24 h, measured by flow cytometry. P-values were calculated using an unpaired two-tailed Student's *t*-test. (**E**-**J**) Representative histogram and quantification of CD80 (**E** and **F**), CD86 (**G** and **H**), and MHC-II (**I** and **J**) expression by spleen macrophages of BALB/c allo-HCT mice on day 7, treated with pegtarazimod or vehicle from day 0 to 7, measured by flow cytometry. P-value was calculated using an unpaired two-tailed Student's *t*-test (**H**) with Welch's correction (**F**) or Mann-Whitney

test (**J**). (**K** and **L**) Expression of CD25 on CD8$^+$ (**K**) or CD4$^+$ (**L**) BALB/c T cells co-cultured for 48 h with allogeneic C57BL/6 BMDM that were pre-treated with pegtarazimod or vehicle for 24 h, measured by flow cytometry. P-values were calculated using Kruskal-Wallis test with Dunn's multiple comparisons test (**K**) or an ordinary one-way ANOVA with Holm-Šídák's multiple comparisons test (**L**).

Supplemental Figure 4: Additional data for neutrophil depletion and p47phox-/- chimera models.

(**A**) Abundance of neutrophils in the bone marrow of neutrophil-depleted (anti-Ly6G) or control (anti-isotype) BALB/c allo-HCT mice treated with pegtarazimod or vehicle from day 0 to 7, measured by flow cytometry. (**B**) Schematic overview of the allo-HCT model with ROS-deficient *p47*$^{phox-/-}$ chimera recipients. (**C**) Engraftment of CD45.2$^+$ cells in the blood of CD45.1 recipient mice after syngeneic transplantation of *p47*$^{phox-/-}$ or wildtype bone marrow, measured by flow cytometry two weeks after transplant.

Supplemental Figure 5: Additional data for SI organoid viability and aGVHD models with anti-TNF treatment.

(**A**) Viability of small intestinal organoids derived from BALB/c mice, treated with TNF and vehicle or vehicle only for 2 days, measured by flow cytometry. Results are derived from three independent experiments. P-values were calculated using an unpaired Student's *t*-test. (**B**) Viability of small intestinal organoids derived from BALB/c mice, treated with menadione for 3 h, measured by flow cytometry. Results are derived from three independent experiments. P-values were calculated using Kruskal-Wallis test with Dunn's multiple comparisons test. (**C**-**E**) GVHD histopathological scores of small intestine (**C**), colon (**D**), and liver (**E**) of BALB/c allo-HCT mice on day 7, treated with pegtarazimod or vehicle from day 0 to 7, and with anti-TNF or anti-isotype on day -1, 1, 3, and 5. P-values were calculated using Kruskal-Wallis test with Dunn's multiple comparisons test.

Supplemental Figure 6: LC-MS metabolite analysis of lipids in SI organoids.

(**A** and **B**) LC-MS metabolite analysis of small intestinal organoids derived from BALB/c mice, challenged with TNF and treated with pegtarazimod or vehicle for 2 days. (**A**) Differential abundance of lipids measured with negative ionization. (**B**) Differential abundance of lipids measured with positive ionization.

Supplemental Figure 7: Kinase activity profiling of SI organoids.

(**A**-**C**) PamDx kinase activity profiling of small intestinal organoids derived from BALB/c mice, challenged with TNF and treated with pegtarazimod for 2 days, compared with organoids challenged with TNF and treated with vehicle for 2 days. (**A** and **B**) Dotplots of significant phosphotyrosine kinases (**A**) and serine-threonine kinases (**B**), clustered by families. Dot color represents $\log_2$ fold change and dot size represents specificity. (**C**) Pathway analysis using metascape. Top 10 most-enriched (ranked by -log10(p value)) clusters. Bar color represents median kinase statistic (positive or negative fold change), weighed by median final score (mean significance score + mean specificity score) of all differentially active kinases in the pathway.

Supplemental Figure 8: Protein levels in clinical study.

(**A**-**F**) Cytokine levels in the plasma of SR-aGVHD patients in the AURORA trial, treated with 10 mg/kg pegtarazimod for 7 days, measured using NULISA. Shown are NULISA Protein Quantification (NPQ) units ($\log_2$-transformation of normalized target counts). (**A**) IFN-γ, (**B**) IL-12p70, (**C**) TNF, (**D**) MCP-1/CCL2, (**E**) IL-1β, and (**F**) IL-6. P values were calculated using a paired t test (**A** and **C**-**F**), or Wilcoxon test (**B**). (**G** and **H**) Plasma levels of NE (**G**), and C5b-9 (**H**). P-values were calculated using a paired t test.

Supplemental Methods

BMDM culture

BM from C57BL/6 mice was isolated, and erythrocytes were lysed as described. 3.5 x 10^6 cells were cultured on non-tissue-culture-treated petri dishes in RPMI 1640 supplemented with 10% FCS, 1% penicillin/streptomycin, and 20 ng/ml macrophage colony-stimulating factor (M-CSF) (Thermo Fisher Scientific). After undisturbed incubation for 4 days at 37 °C and 5% CO_2, cells were incubated for 3 more days with a daily change of culture medium. The next day, cells were washed with 10 mM EDTA, scraped off in PBS, and reseeded to tissue-culture-treated plates. Cells were allowed to adhere for 4 h and treated with 5 mg/ml pegtarazimod or 2.625 mM vehicle. After incubation overnight, cells were analyzed by flow cytometry.

BMDM T cell co-culture

BMDM were generated as described above. On day 6, allogeneic BALB/c T cells were isolated from the spleen using a Pan T cell isolation kit (Miltenyi Biotec) and cultured in RPMI 1640 supplemented with 10% FCS, 1% penicillin/streptomycin, 50 µM 2-Mercaptoethanol, and 30 U/ml IL-2. On day 7, BMDM were reseeded and treated with 5 mg/ml pegtarazimod or 2.625 mM vehicle for 24 h. On day 8, BMDM medium was removed and 200,000 T cells were added to the BMDM culture in RPMI 1640 supplemented with 10% FCS, 1% penicillin/streptomycin, 50 ng/ml M-CSF, 50 µM 2-Mercaptoethanol, and 30 U/ml IL-2. After incubation for 48 h, T cell activation was analyzed by flow cytometry.

Kinase profiling

For profiling of phosphotyrosine kinases (PTK) and serine-threonine kinases (STK), organoids were challenged with TNF and treated with pegtarazimod or vehicle as described. Organoids were collected and Matrigel was dissolved by incubation at 4 °C for 30 min in Cell Recovery Solution (Merck). After three washes with PBS, Organoids were pelleted and stored at -80 °C. PamDx performed kinase profiling on their PamChip microarrays, quality control, phosphosite analysis, upstream kinase analysis and pathway analysis using metascape (3.5).

Supplemental Tables

Supplemental Table 1: Patient characteristics

Total number of patients		8
Age (years)	Median	62
	Range	38-70
Sex	Male	5 (63%)
	Female	3 (38%)
Primary Diagnosis	AML	5 (63%)
	ALL	1 (13%)
	MDS	1 (13%)
	CMML	1 (13%)
Conditioning	RIC	4 (50%)
	MAC	1 (13%)
	Not known	3 (38%)
Donor type	HLA-matched	2 (25%)
	Not known	6 (75%)
Graft source	PBMC	8 (100%)
CMV serostatus	Positive	1 (13%)
	Not known	7 (88%)
Overall GVHD grade	3	6 (75%)
	4	2 (25%)
GVHD Prophylaxis	Tac/MMF	1 (13%)
	Tac/MTX	1 (13%)
	Tac/Sirolimus	2 (25%)
	Tac alone	2 (25%)
	MMF/Everolimus/ATG	1 (13%)
	CyA/Everolimus	1 (13%)

AML, acute myeloid leukemia; ALL, acute lymphoblastic leukemia; MDS, myelodysplastic syndrome; CMML, chronic myelomonocytic leukemia; RIC, reduced-intensity conditioning; MAC, myeloablative conditioning; HLA, human leukocyte antigen; PBMC, peripheral blood mononuclear cell; CMV, cytomegalovirus; GVHD, graft-versus-host disease; Tac, Tacrolimus; MMF, mycophenolate mofetil; MTX, methotrexate; ATG, antithymocyte globulin, CyA, cyclosporin A.

Supplemental Table 2: Severe adverse events

AE	≥ grade 3, related to study drug	0
	leading to permanent study drug discontinuation	0
	leading to death[A], not related to study drug	4 (50%)
Infusion related reaction		0
SAE	related to study drug	0

[A]AE leading to death:

- One patient died from unappreciated catastrophic liver failure at enrollment leading to coagulopathy and gastric bleeding/exsanguination
- One patient died from presumed lung infection (no organism)
- One patient died from aspiration pneumonia, kidney failure and subsequent respiratory failure
- One patient died from multisystem organ failure due to underlying disease

AE, adverse events; SAE, severe adverse events

Supplemental Table 3: Kits

Name	Number	Vendor
Pan T Cell Isolation Kit II, mouse	130-095-130	Miltenyi Biotec
Neutrophil Isolation Kit, mouse	130-097-658	Miltenyi Biotec
Myeloperoxidase (MPO) Peroxidation Activity Assay Kit (Fluorometric)	ab273334	Abcam
Mouse Myeloperoxidase ELISA Kit (MPO)	ab275109	Abcam
Mouse Neutrophil Elastase ELISA Kit	ab252356	Abcam
Transcription Factor Staining Buffer Set	00-5523-00	Thermo Fisher Scientific
Cytofix/Cytoperm Fixation/Permeabilization Kit	554714	BD Biosciences
LDH-Glo Cytotoxicity Assay	J2381	Promega
RNeasy Micro Kit	74004	Qiagen
RNeasy Mini Kit	74104	Qiagen
High-Capacity cDNA Reverse Transcription Kit	4368813	Thermo Fisher Scientific
High Sensitivity RNA ScreenTape Analysis	5067-5579	Agilent
LightCycler 480 SYBR Green I Master	04887352001	Roche
RNA ScreenTape Analysis	5067-5576	Agilent
GeneChip WT PLUS Kit	902281	Thermo Fisher Scientific
Clariom S Assay, mouse	902930	Thermo Fisher Scientific

Supplemental Table 4: Reagents

Name	Number	Vendor
BD Horizon Brilliant Stain Buffer	563794	BD Biosciences
BSA	3854.3	Carl Roth
Cell Recovery Solution	354253	Merck
DAPI	D8417	Merck
DHR123	D1054	Merck
EDTA	CN06.3	Carl Roth
Eosin	3137.2	Carl Roth
FcR Blocking Reagent, human	130-059-901	Miltenyi Biotec
FCS	AC-SM-0027	anprotec
Formaldehyde solution, 4%	FN-5000-4-1	SAV
GlutaMAX Supplement	35050061	Thermo Fisher Scientific
Hematoxilin	1.09249	Merck

HEPES	15630056	Thermo Fisher Scientific
Horse serum	H0146	Merck
IntestiCult Organoid Growth Medium (Mouse)	06005	StemCell Technologies
LIVE/DEAD Fixable Aqua	L34957	Thermo Fisher Scientific
Matrigel Growth Factor Reduced Basement Membrane Matrix	CLS356231-1EA	Merck
Menadione	M5625	Merck
Mouse IL-2	212-12	Thermo Fisher Scientific
Mouse M-CSF	315-01	Thermo Fisher Scientific
Mouse TNF	315-01A	Thermo Fisher Scientific
MEM Non-Essential Amino Acids Solution (100X)	11140035	Thermo Fisher Scientific
2-Mercaptoethanol	31350010	Thermo Fisher Scientific
NaN_3	K305.1	Carl Roth
Normal goat serum	LIN-ENG9010	Biozol
Penicillin/Streptomycin	15140	Thermo Fisher Scientific
Phalloidin Labeling Probes – Alexa Fluor 647 (1:400)	A22287	Thermo Fisher Scientific
PEG	81210	Merck
PMA	Tlrl-pma	InvivoGen
RPMI 1640 Medium	11875093	Thermo Fisher Scientific
Sodium azide NaN_3	K305.1	Carl Roth
Sodium Pyruvate (100 mM)	11360070	Thermo Fisher Scientific
Target Retrieval Solution, Citrate pH 6	S236984-2	Agilent
Taurine	T0625	Merck
Triton X-100	T8787	Merck
Zombie NIR	423106	BioLegend

Supplemental Table 5: In vivo antibodies

Antigen	Clone	Isotype	Number	Vendor
Anti-mouse Ly-6G	1A8	Rat IgG2a, κ	BE0075-1	Bio X Cell
Anti-mouse TNFα	TN3-19.12	Hamster IgG	T258	Leinco Technologies
Anti-rat IgG2a isotype control, anti-trinitrophenol	2A3	Rat IgG2a, κ	BE0089	Bio X Cell
Armenian Hamster IgG Isotype Control	PIP	Hamster IgG	I-140	Leinco Technologies

Supplemental Table 6: Antibodies for immunofluorescence microscopy

Antigen	Fluoro-chrome	Clone	Isotype	Dilu-tion	Number	Vendor
Anti-TUBA4A	unconju-gated	B-5-1-2	Mouse IgG1	1:100	T5168	Merck
Anti-pegtarazimod	unconju-gated	polyclonal	Rabbit	1:1000	custom-made	custom-made
Anti-Histone H3 (citrulline R2 + R8 + R17)	unconju-gated	polyclonal	Rabbit IgG	1:8.33	ab5103	Abcam

Anti-Myeloperoxi-dase	unconju-gated	EPR20257	Rat IgG2a (Chimeric)	1:400	ab300650	Abcam
Anti-H2A.X Phospho (Ser139)	Alexa Fluor 488	2F3	Mouse IgG1, κ	1:50	613406	BioLegend
Anti-Rat IgG H&L	Alexa Fluor 488	polyclonal	Goat	1:500	A11006	Thermo Fisher Scientific
Anti-Rabbit IgG H&L	Alexa Fluor 647	polyclonal	Goat	1:100	PIA32733	Thermo Fisher Scientific
Anti-Rabbit IgG H&L	Alexa Fluor 488	polyclonal	Goat	1:500	ab150077	Abcam
Anti-Rabbit IgG H&L	Alexa Fluor 568	polyclonal	Goat	1:500	ab175471	Abcam
Anti-Mouse IgG H&L	Alexa Fluor 647	polyclonal	Donkey	1:500	A31571	Thermo Fisher Scientific

Supplemental Table 7: Antibodies for flow cytometry

Antigen	Fluorochrome	Clone	Number	Vendor
CD11b	BUV805	M1/70	568345	BD Biosciences
CD11b	BV510	M1/70	101263	BioLegend
CD11b	PerCP/Cyanine5.5	M1/70	101228	BioLegend
CD11c	BV785	N418	117336	BioLegend
CD14	BV605	M5E2	301834	BioLegend
CD16	BUV496	3G8	612944	BD Biosciences
CD16/32	unconjugated	93	101302	BioLegend
CD66b	PerCP/Cyanine5.5	G10F5	305108	BioLegend
CD80	Pacific Blue	16-10A1	104724	BioLegend
CD80	PE	16-10A1	104708	BioLegend
CD86	Pacific Blue	GL-1	105022	BioLegend
CD86	PE/Cyanine5	GL-1	105016	BioLegend
F4/80	Alexa Fluor 488	BM8	123120	BioLegend
F4/80	BUV737	T45-2342	749283	BD Biosciences
IL-1 beta	FITC	JK1B-1	508206	BioLegend
IL-1 beta (Pro-form)	FITC	NJTEN3	11-7114-80	Thermo Fisher Scientific
IL-1 beta (Pro-form)	PE-Cyanine7	NJTEN3	25-7114-82	Thermo Fisher Scientific
Ly-6C	BV711	HK1.4	128037	BioLegend
Ly-6G	BUV615	1A8	751263	BD Biosciences
Ly-6G	Pacific Blue	1A8	127611	BioLegend
MCP-1	PE	2H5	505904	BioLegend
MHC-II	BV510	M5/114.15.2	107636	BioLegend
TNF-α	BV421	MP6-XT22	506328	BioLegend
TNF-α	BV650	MP6-XT22	506333	BioLegend

Supplemental Table 8: Primer sequences

Gene	Forward 5' → 3'	Reverse 5' → 3'
Gapdh	CATCACTGCCACCCAGAAGACTG	ATGCCAGTGAGCTTCCCGTTCAG
Hprt	AGCCTAAGATGAGCGCAAGT	TTACTAGGCAGATGGCCACA
Il1b	GCAACTGTTCCTGAACTCAACT	ATCTTTTGGGGTCCGTCAACT
Mpo	CGTGTCAAGTGGCTGTGCCTAT	AACCAGCGTACAAAGGCACGGT

Phase 2 Open Label Prospective Dose-Ranging Clinical Trial with Escalation and Expansion Cohorts to Evaluate Safety, Tolerability, Pharmacokinetics, Pharmacodynamics, Dosing, and Efficacy of RLS-0071 for the Treatment of Hospitalized Patients with Steroid-Refractory Acute Graft-versus-Host Disease
Protocol Number: RLS-0071-203

Compound:	RLS-0071
Study Phase:	Phase 2
Sponsor Name:	ReAlta Life Sciences, Inc.
Legal Registered Address:	5665 Lowery Road<br>Norfolk, VA 23502, USA
IND	166275
EU CTR No.	2024-510582-42
Protocol Date	29 April 2025 (Amendment 8)
Sponsor's Responsible Medical Officer:	Kenji M. Cunnion, MD, MPH<br>Chief Medical Officer<br>ReAlta Life Sciences, Inc.<br>Phone: +1 757-901-0460<br>Email: kcunnion@realtals.com
Medical Monitor	Richard Curry III, MD<br>Medical Director<br>CTI Clinical Trial and Consulting<br>100 E. RiverCenter Blvd.<br>Covington, KY 41011

Compliance Statement

This trial will be conducted in compliance with the protocol and in accordance with the ethical principles of Good Clinical Practice (GCP), according to the International Council for Harmonisation (ICH) Harmonised Tripartite Guideline and all applicable regulatory requirements.

Signature Page

Sponsor's Responsible Medical Officer

Protocol Title:	Phase 2 Open Label Prospective Dose-Ranging Clinical Trial with Escalation and Expansion Cohorts to Evaluate Safety, Tolerability, Pharmacokinetics, Pharmacodynamics, Dosing, and Efficacy of RLS-0071 for the Treatment of Hospitalized Patients with Steroid-Refractory Acute Graft-versus-Host Disease
Protocol Number:	RLS-0071-203
Date:	29 April 2025 (Amendment 8)

____ ____________________

Kenji M. Cunnion, MD, MPH
Chief Medical Officer
ReAlta Life Sciences, Inc.
5665 Lowery Road
Norfolk, VA 23502, US

Date

Investigator Agreement

I have received and read the Investigator's Brochure for RLS-0071. I have read Protocol RLS-0071-203 and agree to conduct the study as outlined. I agree to maintain the confidentiality of all information received or developed in connection with this protocol.

____
Printed Name of Investigator

______________________________ ______________
Signature of Investigator Date

1. Study Overview

1.1 Synopsis

**Study Title:**<br>Phase 2 Open Label Prospective Dose-Ranging Clinical Trial with Escalation and Expansion Cohorts to Evaluate Safety, Tolerability, Pharmacokinetics, Pharmacodynamics, Dosing, and Efficacy of RLS-0071 for the Treatment of Hospitalized Patients with Steroid-Refractory Acute Graft-versus-Host Disease
**Sponsor:**<br>ReAlta Life Sciences, Inc<br>5665 Lowery Road<br>Norfolk, VA 23502, USA
**Study Intervention:**<br>RLS-0071
**Study Number:**<br>RLS-0071-203
**Study Sites:**<br>US and EU sites anticipated: 10-20
**Phase of Development:**<br>Phase 2
**Study Objectives:**<br>Co-Primary Objectives:<br>• Demonstrate safety and tolerability of RLS-0071 in the treatment of acute graft versus host disease (aGvHD).<br>• Determine Overall Response Rate (ORR) of RLS-0071 at 28 days.<br>Secondary Objectives:<br>• Determine Complete Response (CR) Rate, Very Good Partial Response (VGPR) Rate, and Partial Response (PR) Rate at 7, 14, 28, 56, and 180 days.<br>• Determine ORR at 7, 14, 56, and 180 days.<br>• Determine ORR, and CR Rate, VGPR Rate, and PR Rate at 7, 14, 28, 56, and 180 days based on lower gastrointestinal (GI) response only (overall study population and for the subset with lower GI aGvHD).<br>• Assess refractoriness to RLS-0071 +/- ruxolitinib.<br>• Determine need for initiation of additional or alternative treatments for aGvHD.<br>• Evaluate change in Stage and attainment of Stage 0 or 1 for lower GI aGvHD, liver aGvHD, skin aGvHD, and upper GI aGvHD, and overall aGvHD Grade from baseline to Days 7, 14, 28, 56, and 180.<br>• Assess loss of response, reduction in response, or occurrence of flare in participants who had an initial response to RLS-0071 (with systemic corticosteroids and +/- ruxolitinib).<br>• Evaluate dose response (dose and duration) for the primary and secondary efficacy endpoints.<br>• Characterize overall survival, failure-free survival, and non-relapse mortality.

- Characterize Pharmacokinetics (PK) (based on sparse sampling for all participants and intensive sampling for a subset of participants) of RLS-0071.
- Evaluate aGvHD participant outcomes: FACT-BMT, abdominal pain, volume/frequency of diarrhea, food tolerance and use of total parenteral nutrition (TPN), and duration of hospital stay.
- Evaluate change in Mount Sinai Acute GVHD International Consortium (MAGIC) biomarkers (ST2 and REG3a) at Days 7, 14, and 28.

Exploratory Objectives:

- Evaluate pharmacodynamic (PD) biomarkers (e.g., for assessment of aGvHD and effects of RLS-0071).
- Calculate MAGIC algorithm probability (MAP) and assess correlation of MAP with clinical efficacy and safety.
- Assess ability to taper corticosteroids and characterize overall corticosteroid use at Days 7, 14, 28, 56, and 180.

Study Design:

Phase 2 Open Label Prospective Dose-Ranging Escalation and Expansion Trial to Evaluate Safety, Tolerability, Pharmacokinetics, Pharmacodynamics, Dosing, and Efficacy of RLS-0071 for the secondary treatment of acute Graft-versus-Host Disease (aGvHD) in hospitalized participants who are steroid-refractory. Participants will be enrolled after meeting criteria for steroid-refractory aGvHD, and RLS-0071 will be initiated. Ruxolitinib will also be initiated unless ruxolitinib is contraindicated or considered inappropriate treatment for a specific study participant. No other (secondary) aGvHD treatments are permitted (unless aGvHD progresses further or does not improve after 7 days). Systemic corticosteroids and GvHD prophylactic medications should be continued according to the schedules detailed in Section 7.5.

RLS-0071 will be administered for 7 or 14 days according to the assigned dose group. RLS-0071 should ideally be administered simultaneously to the time of ruxolitinib, but the time interval between ruxolitinib and RLS-0071 initiations must be less than 48 hours. Note that as part of the study design, when ruxolitinib is planned from the time of study initiation and is administered after RLS-0071 initiation but within 48 hours, then the "addition" of ruxolitinib will not be considered as an RLS-0071 treatment failure. The systemic corticosteroid dose should be >2 mg/kg methylprednisolone or equivalent for initial treatment of aGvHD prior to enrollment; tapering schedules are described in Section 7.5.2.

Adults or adolescents (>12 years old) hospitalized with steroid refractory aGvHD after allogeneic hematopoietic stem cell transplantation (allo-HSCT) with any conditioning regimen will be enrolled (see inclusion criteria for specific population definition). aGvHD will be characterized according to the MAGIC criteria as Grade II, III, or IV, defined as:

- Grade II: Stage 3 rash and/or Stage 1 liver and/or Stage 1 upper GI and/or Stage 1 lower GI
- Grade III: Stage 2–3 liver and/or Stage 2–3 lower GI, with Stage 0–3 skin and/or Stage 0-1 upper GI
- Grade IV: Stage 4 skin, liver, or lower GI involvement, with Stage 0–1 upper GI

Note: Patients with Stage 3 or 4 liver are excluded from participation in the study

Consistent with clinical practice, participants will be enrolled, and RLS-0071 treatment started based on aGvHD clinical criteria, and all the key response criteria will also be based on clinical endpoints. The early intervention with RLS-0071 based on clinical criteria underscores the importance of intervening quickly to treat aGvHD, to avoid disease worsening, and to optimize the likelihood of improvement at the time of steroid refractoriness and avoid the need for third-line treatment.

A lower GI endoscopy with biopsy is recommended (as part of standard of care) for participants with signs and symptoms of lower GI aGvHD, as long as the endoscopy (typically a sigmoidoscopy) +/- biopsy are anticipated to be safe and feasible for the participant. If the endoscopy and biopsy definitively rule out a diagnosis of lower GI aGvHD for participants who would only qualify for the study based on lower GI aGvHD (i.e., they do not have Grade >II aGvHD based on skin, liver [Stage 1 or 2], or upper GI aGvHD), then the participant will be discontinued from the study and replaced. If a lower GI infection (e.g., cytomegalovirus [CMV], *Clostridioides difficile* [Cdiff]) is diagnosed, then the participant can be enrolled as long as the infection is not uncontrolled and appropriate anti-infective treatment is being initiated and administered.

Participants should continue prophylactic aGvHD medications during (and potentially beyond) the 7-day or 14-day RLS-0071 treatment period (see Section 7.5.3 for specific parameters).

Importantly, participants who initiate medications other than systemic corticosteroids to treat aGvHD prior to initiating RLS-0071 will be excluded. If ruxolitinib had been initiated prior to RLS-0071 to treat aGvHD (e.g., skin aGvHD), that is acceptable as long as RLS-0071 is able to be initiated within 48 hours after ruxolitinib was initiated and there is no evident failure of ruxolitinib treatment.

Participants who require treatment for conditions other than aGvHD should receive those treatments, including appropriate standard-of-care antifungal prophylaxis, antibiotics for fever, antivirals for CMV, pain medications, dysmotility drugs, and TPN, etc.

Duration of RLS-0071 treatment: RLS-0071 treatment will be administered for 7 days in Cohort 1 and for 14 days in Cohorts 2, 3, 4 and 5. For the one or two planned dosing arms of the expansion cohorts, the dose of RLS-0071 will be determined based on safety, pharmacokinetics (PK), pharmacodynamics (PD), and efficacy findings in the dose-escalation cohorts, and are planned to be 14 days in treatment duration.

Use of corticosteroids: Corticosteroids should have been prescribed according to standard of care prior to enrollment with dosing of methylprednisolone (MPE) (or equivalent) until fulfilling criteria for the standard definition of steroid-refractory aGvHD. Steroid-refractory aGvHD as defined in the Inclusion Criteria: progressed after 3 days of treatment with methylprednisolone equivalents (MPE) >2 mg/kg/day; did not improve after 7 days of treatment with MPE >2 mg/kg/day; progressed to a new organ after treatment with MPE >1 mg/kg/day for isolated skin and/or upper GI GvHD; or recurred during or after a steroid taper.

Clinically, there is a balance between enabling a clinical response and avoiding steroid adverse effects when considering the timing of steroid tapering. The corticosteroid taper is standardized in the protocol (see Section 7.5.2).

Secondary treatment: During the initial 7 days of RLS-0071 +/- ruxolitinib treatment, other secondary treatments are not permitted (corticosteroids are expected to continue). After Day 7, alternative treatments are not permitted for participants with a complete response and are discouraged for participants who have attained a very good partial response.

Tertiary treatment: Tertiary treatments may be needed for non-responders or for those participants whose aGvHD progresses. Alternative (i.e., tertiary) treatment is only permitted after at least 3 days of RLS-0071 treatment if there is clear evidence of aGvHD disease progression or if RLS-0071 was discontinued (e.g., for safety). After Day 7, alternative treatment(s) should be initiated for non-responders or for those with disease progression.

Any participant whose GvHD progresses or who receives an alternative therapy would be considered a treatment failure.

Ruxolitinib is standardly tapered after 6 months (180 days) in participants who have clinical response and have discontinued therapeutic doses of corticosteroids. Accordingly, ruxolitinib should not be tapered during the 180-day duration of this clinical study, unless clinically indicated.

Ruxolitinib is associated with thrombocytopenia, anemia, and neutropenia. These adverse reactions have not been observed in the RLS-0071 nonclinical and clinical phase 1 studies and are not expected to occur based on the RLS-0071 mechanism of action. If thrombocytopenia, anemia, or neutropenia occur on study, this should be managed by ruxolitinib dose reduction, or interruption, or transfusion according to the approved prescribing information for ruxolitinib. For example, for an absolute neutrophil count less than 1 x 10^9/L considered related to ruxolitinib, hold ruxolitinib for up to 14 days and then resume at 1 dose level lower upon recovery.

If participants receiving RLS-0071 are discharged from the hospital, which is unlikely in this time period considering the typical disease severity, they can continue to receive RLS-0071 as outpatients for the duration of the treatment course. Dosing of RLS-0071 as outpatient will be once daily (QD). Participants in Cohort 2a or Cohort 3 who improve and are able to leave the hospital after 7 to 13 days of treatment with RLS-0071 will be given the opportunity to roll over their enrollment into Cohort 4 or Cohort 5 if they are determined to be eligible after discussion between the PI and Medical Monitor. The outpatient dosing for Cohort 4 and Cohort 5 will be once a day (QD) following discharge to outpatient to completion of Day 14 (approximate range of 28 - 42 total doses of RLS-0071).

Treatment assignment: Participants will be followed for 180 days for safety and efficacy, including durability of initial response.

The study will enroll approximately 21 participants initially, with the potential to expand enrollment to up to 66 participants, and the study design comprises:

Dose Escalation Cohorts, Enrolled sequentially based on protocol-specified dose escalation criteria.

7-day dosing:

- **Cohort 1a:** 9 participants will receive 10 mg/kg Q8H RLS-0071 for 7 days with concurrent ruxolitinib. Completion of Cohort 1a will lead to the opening of Cohort 2a, if appropriate.
- **Cohort 1b:** 3 participants will receive 10 mg/kg Q8H RLS-0071 for 7 days without concurrent ruxolitinib. Opens concurrent to Cohort 1a. Completion of Cohort 1b will lead to opening of Cohort 2b, if appropriate. (Based upon DLT findings or Data Safety Monitoring Board (DSMB) recommendation, the cohort may be expanded to n=6)

14-day dosing in hospitalized participants:

- **Cohort 2a:** 6 participants will receive 10 mg/kg Q8H RLS-0071 for 14 days with concurrent ruxolitinib. Completion of Cohort 2a will either lead to the opening of Cohort 3 or lead to the opening of Expansion Cohort 1, based on safety and clinical response data review.
- **Cohort 2b:** 3 participants will receive 10 mg/kg Q8H RLS-0071 for 14 days without concurrent ruxolitinib. This cohort will not open prior to the opening of Cohort 2a. (Based upon DLT findings or DSMB recommendation, the cohort may be expanded to n=6)
- **Cohort 3:** 6 participants will receive 20 mg/kg Q8H RLS-0071 for 14 days with concurrent ruxolitinib. Completion of Cohort 3 will progress to the opening of Expansion Cohort 2, if appropriate.

If a participant in Cohort 2a or 3 is rolled into Cohort 4 or 5, then they will be replaced within Cohort 2a or 3.

14-day dosing with 7 to 13 days inpatient and continued outpatient treatment through Day 14:

Participants in Cohort 2a or Cohort 3 who improve and are able to leave the hospital after 7 to 13 days of treatment with RLS-0071 likely represent a less severe population (e.g., skin disease without intestinal disease). Thus, these participants that are appropriate for hospital discharge after 7 to 13 days are a reasonable population to segregate into outpatient management as would normally be done for participants well enough to go home and are desiring to go home. These less severe participants in Cohort 2a and Cohort 3 will be given the opportunity to roll over into the following cohorts. Determination of eligibility and appropriateness to roll over will be made by the PI in discussion with

the participant as well as the Medical Monitor. These participants will be followed as a separate cohort to determine disease characteristics that are likely different from those requiring continued hospitalization.

- **Cohort 4:** up to 3 participants will receive 10 mg/kg Q8H RLS-0071 for 7 to 13 days and then 10 mg/kg QD RLS-0071 to complete 14 days with concurrent ruxolitinib.
- **Cohort 5:** up to 3 participants will receive 20 mg/kg Q8H RLS-0071 for 7 to 13 days and then 20 mg/kg QD RLS-0071 to complete 14 days with concurrent ruxolitinib.

Dose Expansion Cohorts (1 or 2 dosing arms, up to 24 participants total)

- **Expansion Cohort 1:** 12 participants will receive 10 mg/kg Q8H RLS-0071 for 14 days.
- **Expansion Cohort 2:** 12 participants will receive 20 mg/kg Q8H RLS-0071 for 14 days.

Additional participants may be added to any Cohort based on DSMB recommendation.

Safety and available PK, PD, and efficacy data will be evaluated for each cohort, prior to advancing to each subsequent dose cohort or into the expansion cohorts. A DSMB will be in place to review these data and make recommendations to the Sponsor. All participants will be followed for at least 14 days in each cohort prior to dose escalation. Additionally, regimens shown to be safe in the dose escalation cohorts will be selected for further evaluation in 1 or 2 dose expansion cohorts based on safety, PK, PD, and efficacy. The specific criteria for dose escalation and expansion are defined in the protocol (see Section 8.3).

The study design allows for evaluation of 2 dose levels of RLS-0071 (10 mg/kg and 20 mg/kg), and safety permitting of 7-day vs. 14-day dosing of RLS-0071. This design will be informative for comparative efficacy, safety, pharmacokinetics, pharmacodynamic response, and dose response for RLS-0071.

PK Sampling: Intense PK samples will be collected and evaluated for 3 participants each from dose escalation Cohorts 1a and 3 (6 participants total for intense PK) during the first 7 days of dosing for participants treated with 10 or 20 mg/kg RLS-0071. The remaining study participants will have only sparse PK collected and evaluated.

RLS-0071 should be used with caution upon concomitant administration with sensitive CYP2C19 substrate.

Immunogenicity Assessment: Samples will be collected to evaluate for potential anti-drug antibodies against RLS-0071. Samples will be collected prior to the first dose of RLS-0071 and on Day 28 for all participants. If the Day 28 result is positive, then a repeat sample will be collected on Day 180. The immunogenicity assessment plan and schedule will be described in further detail in the protocol (see Section 9.4).

Additionally, while the study is designed without a concurrent non-RLS-0071 control group, the study efficacy data will also be evaluated in the context of historical control data, including those studies evaluated as part of the ruxolitinib development program for steroid-refractory aGvHD.

Study Rationale:

ReAlta Life Sciences, Inc (Sponsor) has developed a new class of peptides that specifically inhibit multiple inflammatory pathways including the classical and lectin pathways of complement, myeloperoxidase (MPO) and neutrophil extracellular trap (NET) formation. Preliminary preclinical data in a standard mouse model of aGvHD showed that RLS-0071 greatly increased survival and significantly decreased end-organ damage, this is further described in the Investigator Brochure. RLS-0071 has completed a healthy human volunteer Phase 1 clinical trial that defined the PK parameters in healthy adult humans and demonstrated no significant toxicities for any of the doses tested. RLS-0071 has also completed a Ph1b clinical trial in Germany of healthy volunteers inhaling lipopolysaccharide (LPS) simulating an acute neutrophil-driven disease process. This trial demonstrated no toxicities associated with RLS-0071 and showed clinically meaningful

pharmacodynamic effects measured by a reduction in sputum lung neutrophils, myeloperoxidase, NETosis markers, complement, and cytokine levels.

For patients receiving allo-HSCT approximately 60% will develop aGvHD of Grades II – IV, moderate to very severe. The most severe form of aGvHD and that with the greatest unmet medical need is lower GI aGvHD which involves the intestines and leads to pain, diarrhea, malabsorption, and malnutrition although liver, skin, and upper GI aGvHD may also be severe and manifest individually or with lower GI aGvHD. Due to the severity of disease (including the high volume of diarrhea) and the need for systemic IV corticosteroids, these lower GI aGvHD patients are typically hospitalized for weeks including many of those with Stage I lower GI aGvHD and essentially all of those with Stages II, III, or IV lower GI aGvHD. Many patients with liver, skin, or upper GI aGvHD may also require hospitalization.

The initial treatment for aGvHD is systemic corticosteroids, which is the current standard-of-care primary treatment. Corticosteroids do not control aGvHD in approximately half of patients treated; these patients are then considered steroid refractory and require secondary treatments such as ruxolitinib. This initial study of RLS-0071 in the treatment of aGvHD focuses on steroid refractory patients. Ruxolitinib is the only currently approved medication for the treatment of steroid refractory aGvHD, and this study treats patients with RLS-0071 and ruxolitinib together, unless ruxolitinib is contraindicated or inadvisable for a specific patient. It is expected that the systemic corticosteroids will continue, despite the occurrence of steroid refractoriness. The goal of adding RLS-0071 to ruxolitinib treatment as secondary treatment for aGvHD (in steroid refractory patients) is to attain better disease control and avoid the need for other secondary or tertiary treatments to improve the overall response rate.

Of note, even for those patients who had responded to corticosteroids, the response is often partial and/or not durable. Mortality is high in all of these patients, especially those who become refractory or lose initial response. While ruxolitinib is indicated as secondary treatment for aGvHD in patients who become steroid refractory, this drug is ineffective in almost half the patients treated. Therefore, there remains important unmet need for a fast-acting, safe, and effective therapy that could be used in secondary aGvHD (initially with ruxolitinib in this clinical study), and subsequently potentially as a secondary treatment without ruxolitinib and/or in earlier settings of aGvHD (e.g., in combination with systemic corticosteroids for initial treatment of aGvHD to improve response rates, avoid refractoriness, and prevent the need for secondary aGvHD treatments).

The pathogenesis of aGvHD is multifaceted, but the unresponsiveness of at least half the patients to corticosteroids strongly suggests that factors other than lymphocytes must play an important role. Recent work suggests that much of the pathogenesis is driven by the conditioning therapy causing endothelial injury followed by neutrophil activation, NETosis, and complement activation. Thus, there remains a significant unmet medical need and therapeutic gap in terms of addressing these important pathogenic mechanisms, which are not addressed by corticosteroids alone nor with ruxolitinib.

The ability of RLS-0071 to inhibit both complement and neutrophil mediated inflammation suggests that this drug may have efficacy for preventing or treating and reversing the pathogenesis of aGvHD and the intestinal manifestations in particular.

The population for this study is patients who had received allo-HSCT, who have aGvHD, and who have become refractory to systemic corticosteroids and are hospitalized. The main goals of this study are to evaluate the safety/tolerability and Overall Response Rate (ORR) of RLS-0071 in steroid refractory aGvHD. Additional goals include evaluation of dose, pharmacokinetics, pharmacodynamics, and other measures of efficacy.

The primary efficacy endpoint is Overall Response Rate (ORR) at 28 days. ORR (specifically at 28 days) is an accepted primary endpoint for the treatment of aGvHD, and comprises complete response, very good partial response, and partial response, all of which are clinically meaningful assessments. The underlying response criteria (CR, VGPR, and PR) that comprise the ORR assessment evaluate GI,

skin, and liver manifestations of aGvHD. The FDA-approved secondary treatment Jakafi (ruxolitinib) timed their ORR primary endpoint at 28 days in the steroid-refractory participant population.

RLS-0071 is fast-acting and is being developed for treatment of aGvHD, with the goal to enable rapid disease control. Full GI mucosal healing and a full clinical response may take longer than 7 days, and the clinical study design therefore includes dosing regimens of 7 and 14 days duration. Participants will be evaluated at a range of timepoints including 7 and 14 days for rapid response, 28 days, as well as 56 and 180 days for continued improvement and durability of response. Considering the lower GI emphasis within the RLS-0071 treatment goals, the secondary endpoints include ORR and CR, VGPR, and PR that focus on the lower GI manifestations of aGvHD at various timepoints.

In addition to the ORR primary endpoint and a set of other clinical efficacy endpoints, the study includes assessment of the MAGIC biomarkers ST2 and REG3a. The Mount Sinai Acute GVHD International Consortium (MAGIC) algorithm probability (MAP) is derived from these 2 serum biomarkers and measures damage to crypts in the gastrointestinal tract during GvHD. Changes in MAP after treatment suggest that the algorithm can be used as a response biomarker and correlated with clinical outcomes. Additionally, combination of the MAP with clinical endpoints may further predict response. The MAGIC biomarkers should provide further insight into the early clinical response to aGvHD treatment.

The completed Phase 1 (RLS-0071-101) healthy volunteer single ascending dose (SAD)/multiple ascending dose (MAD) PK study and the Phase 1b inhaled LPS proof of mechanism study (RLS-0071-103) included intensive PK sampling, which generated data at the doses being studied in this aGvHD clinical study. Accordingly, this aGvHD clinical study collects sparse PK data on all study participants to confirm and extend the earlier findings in an aGvHD participant population, as well as intense PK on a subset of participants receiving the 10 mg/kg or 20 mg/kg doses to evaluate whether the physiology of aGvHD affects how RLS-0071 is eliminated and metabolized.

Study population:

Adults or adolescents (>12 years old) hospitalized with steroid-refractory aGvHD after allo-HSCT with any conditioning regimen will be enrolled. aGvHD will be characterized as Grade >II aGvHD. The aGvHD staging and grading will be according to the MAGIC guidelines.

Dose Rationale:

RLS-0071 was tested in study RLS-0071-101, a Phase 1 healthy volunteer SAD/MAD design. RLS-0071 demonstrated no safety signals in healthy volunteers at doses of up to 40 mg/kg IV Q8H for 9 doses or single doses up to 120 mg/kg IV. The dosing strategy for aGvHD will utilize a 10 mg/kg IV Q8H for 7 days in Cohort 1, or 10 or 20 mg/kg IV Q8H for 14 days in Cohorts 2, 3, 4 and 5 respectively. This regimen is designed to attain and maintain optimal suppression of inflammation through the typical duration of the most severe gastrointestinal and other aGvHD symptoms.

Consistent with the earlier clinical studies of RLS-0071 as described above, this Phase 2 study in aGvHD initiates enrollment with a 10 mg/kg IV Q8H dose regimen for 7 days. Dosing will be escalated in sequential cohorts as follows: 10 mg/kg IV Q8H dose regimen for 14 days and 20 mg/kg IV Q8H dose regimen for 14 days. Participants will be enrolled in each dose level. Safety/tolerability (for at least 14 days) will be confirmed at each dose level, including DSMB review, before escalating to the next dose level. A period of dosing longer than 7 days addresses the time it may take for full clinical response and gut healing. However, the study also allows for the possibility that clinical response can continue to improve beyond Day 7 based on only 7 days of dosing.

Following the dose escalation cohorts, the study is planned to proceed to a dose expansion component with one or two dose arms based on the safety/tolerability, PK, PD, and efficacy findings in the dose escalation cohorts. Each dose expansion cohort is planned to include up to 12 participants (up to 24 participants total for 2 arms in dose escalation).

Overall, the different dose regimens (dose and duration) and the dose escalation and dose expansion components in the study provide the opportunity to establish safety and efficacy in Phase 2 to determine the RLS-0071 dose regimen to advance further in development.

Planned Number of Participants:

This Phase 2 study will enroll approximately 21 participants initially, with the potential to expand enrollment up to 66 participants, and the study design comprises:

- Dose Escalation:
    - Three sequential cohorts of 6 or more participants each at different dose levels and durations of RLS-0071 in combination with ruxolitinib (approximately 21 participants)
    - Two sequential cohorts at different dose levels and durations of RLS-0071 not in combination with ruxolitinib (up to 12 participants total)
    - Two (2) roll-over cohorts with outpatient management of participants in the 14-day group that are discharged from the hospital treated with RLS-0071 in combination with ruxolitinib (up to 6 total)
- Dose Expansion:
    - One or two cohorts of up to 12 participants each at dose levels informed by the results of the dose escalation (up to 24 total).

Additional participants may be added to any Cohort based on DSMB recommendation.

Study Intervention, Dosage, and Mode of Administration:

Phase 2: RLS-0071 will be administered as an intravenous (IV) infusion in all study participants. Dosing will be 10 mg/kg or 20 mg/kg for either 7 or 14 days. Study intervention will be given in addition to aGvHD systemic corticosteroid standard of care management for at least 7 days and +/- ruxolitinib as an approved secondary treatment for steroid refractory aGvHD. The frequency of RLS-0071 dosing will be 3-times daily through Day 7 or Day 14 depending on cohort and can be reduced to only 1-time daily after Day 7 for participants who have been discharged to the outpatient setting and are unable to continue coming to the clinic three times a day.

Duration of Treatment/Participation:

All participants enrolled in the trial will receive RLS-0071 for 7 days in Cohort 1 or 14 days in Cohorts 2, 3, 4 and 5 during which assessments will be made of safety, PK, efficacy, and PD. Participants will then remain in the study through day 180 to further assess safety and efficacy. Total duration of participation is 180 days following the screening period.

Reference Therapy, Dosage, and Mode of Administration:

No reference therapy is being used.

Inclusion Criteria:

To be enrolled in the study, a participant must meet the following criteria:

1) Male or female adults or adolescents (>12 years old).
2) Have undergone first allogeneic hematopoietic stem cell transplantation (allo-HSCT) from any donor source using bone marrow, peripheral blood stem cells, or cord blood for hematologic malignancies. Recipients of nonmyeloablative and myeloablative conditioning regimens are eligible. Any conditioning regimen and any anti-GVHD prophylactic program are permitted.
3) Steroid-refractory aGvHD defined by one or more of the following criteria: progressed after 3 days of treatment with methylprednisolone equivalents (MPE) >2 mg/kg/day; did not improve after 7 days of treatment with MPE >2 mg/kg/day; progressed to a new organ after treatment with MPE >1 mg/kg/day for isolated skin and/or upper GI GvHD; or recurred during or after a steroid taper.

4) Grade II-IV aGvHD as per MAGIC guidelines, defined as:
   a) Grade II: Stage 3 rash and/or Stage 1 liver and/or Stage 1 upper GI and/or Stage 1 lower GI, or
   b) Grade III: Stage 2 liver and/or Stage 2–3 lower GI, with Stage 0–3 skin and/or Stage 0-1 upper GI, or
   c) Grade IV: Stage 4 skin, liver, or lower GI involvement, with Stage 0–1 upper GI

   Note: Patients with Stage 3 or 4 liver are excluded from participation in the study
5) Hospitalized or being admitted to hospital with aGvHD and a Karnofsky Performance Status (KPS) of at least 50 (Table 3). Inclusion of a participant with a KPS lower than 50 requires discussion with the Medical Monitor.
6) Anticipated hospital length-of-stay of at least 1 week from the time of RLS-0071 initiation.
7) Initiating or recently initiated ruxolitinib for secondary treatment of aGvHD; if ruxolitinib is contraindicated or the investigator considers it inadvisable for a specific participant then that participant can be enrolled in Cohort 1b or Cohort 2b and not treated with ruxolitinib.
8) Feasibility to initiate RLS-0071 dosing simultaneously to ruxolitinib or within 48 hours before or after initiation of ruxolitinib (note that from a timing perspective the goal is simultaneous initiation of RLS-0071 and ruxolitinib whenever possible).
9) No plans to add additional GvHD treatment medications or to add, dose-adjust, or discontinue GvHD prophylactic medications during the 7 days of RLS-0071 treatment. Note that participants who require treatments for conditions other than aGvHD should receive those treatments, including standard-of-care antifungal prophylaxis, antibiotics for fever, antivirals for CMV, pain medications, dysmotility agents, and TPN, etc.
10) Neutrophil recovery following the stem-cell transplantation, defined as blood neutrophil count >500/mL for at least 3 consecutive measurements (use of growth factor supplementation, e.g., filgrastim, at that time is permitted).
11) At the time of study screening prior to RLS-0071 initiation:
   a) Neutrophil count >1000/mL (not supported by growth factor supplementation, e.g., filgrastim);
   b) Platelet count >50,000/ml (not supported by platelet transfusions);
   c) Aspartame aminotransferase (AST) and alanine aminotransferase (ALT) < 5 times the upper limit of normal (ULN);
   d) Serum bilirubin < 6mg/dL; and
   e) Adequate coagulation function (e.g., prothrombin time (PT)/international normalized ratio (INR) and activated partial thromboplastin time (aPPT)/partial thromboplastin time (PTT) <2 X ULN.
12) Weight >40 kg and ≤ 140 kg at Screening.
13) Participant (or legally authorized representative for minors) provides written informed consent; additionally, informed assent for minors.
14) Able to communicate well with the Investigator and/or study site personnel and to comply with the requirements of the entire study.
15) Woman participants of childbearing potential (WOCBP) (not surgically sterile) must agree to use highly effective contraception during the study drug treatment period and for 6 additional months following treatment. Follow manufacture recommendation for other medications.
16) Male participant with partners of childbearing potential must agree to use highly effective contraception during the study drug treatment period and for 3 additional months following treatment. Follow manufacture recommendation for other medications.

Exclusion Criteria:

A participant who meets any of the following criteria at Screening will be excluded from participation in this study:

1) Has received more than 1 allo-HSCT.
2) Current, previous, or planned (in the initial 7 days) use of any systemic treatment in addition to or other than corticosteroids or ruxolitinib (unless initiated within 48 hours of enrollment per Section 6.1) for aGvHD (previously initiated aGvHD prophylaxis is permitted to continue).
3) Previous failure of ruxolitinib treatment.
4) Uncontrolled GI infection (e.g., CMV, Cdiff); note that participants with controlled GI infections can be enrolled as long as appropriate anti-infective treatment is initiated and administered.
5) Endoscopic and biopsy testing (if performed) that definitively rules out lower GI aGvHD in those who are clinically suspected of having lower GI aGvHD; those participants should not be enrolled or should be discontinued from the study (and will be replaced) unless they otherwise meet the study entry criteria for skin, liver, and/or upper GI aGvHD.
6) Chronic GvHD (including presence of GVHD overlap syndrome as per National Institutes of Health [NIH] guidelines).
7) Participants with evidence of relapsed primary disease, or participants who have been treated for relapse after the allo-HSCT was performed.
8) Unresolved toxicity or complications (other than aGvHD) due to the allo-HSCT, which in the opinion of the Investigator would pose a safety risk for participation in this trial.
9) Any corticosteroid therapy for indications other than aGvHD at doses of methylprednisolone or equivalent >1 mg/kg per day within 7 days of enrollment.
10) Severe organ dysfunction unrelated to underlying aGvHD, including:
    a) Cholestatic disorders or unresolved veno-occlusive disease of the liver (defined as persistent bilirubin abnormalities not attributable to GvHD and ongoing organ dysfunction)
    b) Clinically significant or uncontrolled cardiac disease including unstable angina, acute myocardial infarction within 6 months from Day 1 of study drug administration, New York Heart Association Class III or IV congestive heart failure, circulatory collapse requiring vasopressor or inotropic support, or arrhythmia that requires therapy.
    c) Clinically significant respiratory disease that requires mechanical ventilation support or oxygen supplementation.
11) Known hypersensitivity, allergy, or anaphylactic reaction to polyethylene glycol (PEG) or PEG containing material.
12) Severe kidney disease, with an estimated glomerular filtration rate (eGFR) < 30 mL/min/1.73m^2 (chronic kidney disease [CKD] Stages 4-5).
13) Significant liver disease that is unrelated to GvHD
14) Participation in any clinical research study evaluating an investigational product (IP) or therapy for GvHD prophylaxis or treatment within 1 month and less than 5 half-lives of IP prior to the Screening visit.
15) Currently breast feeding.
16) Any medical or psychiatric condition deemed clinically significant by the Investigator or Sponsor such that:
    a) Participation of the study would be unsafe,
    b) OR the condition would make study results uninterpretable.

17) Unable or unwilling to cooperate with the site staff for any reason.
18) Known pregnancy, a positive pregnancy test at screening, or lactation for WOCBP.
19) Active hepatitis B virus (HBV) or hepatitis C virus infection that requires treatment or human immunodeficiency virus (HIV)-1 or HIV-2.
20) Active sepsis.

Concomitant and Prohibited Medications

Participants will receive standard-of-care concomitant systemic corticosteroid treatment (See Section 7.5.2 for specific parameters).

Participants who require treatment for conditions other than aGvHD should receive those treatments, including standard-of-care antifungal prophylaxis, antibiotics for fever, antivirals for CMV, pain medications, dysmotility agents, TPN, etc.

Previously initiated aGvHD prophylactic medications are expected to be continued, at the same dose for at least 7 days on study. Prophylactic medications must be continued as long as initially planned pre-study unless there is a clinical reason (e.g., safety) to taper the prophylactic regimen differently (see Section 7.5.3).

Investigational treatments for aGvHD (other than RLS-0071 study drug) are prohibited. Systemic corticosteroids and ruxolitinib are not considered investigational treatments for aGvHD. Other than corticosteroids, no prior aGvHD treatment is permitted.

Timing and Order of Medication Tapering

RLS-0071 will be administered for 7 or 14 days, based on dose assignment. The medication will then be stopped after the assigned time period is completed.

Systemic corticosteroid dose should be tapered according to one of the standardized tapering regimens (see Section 7.5.2).

Prophylactic medication schedules are typically predetermined. The dose of these previously initiated medications are expected to not be changed during the (initial) 7 days of RLS-0071 treatment, but otherwise should be tapered or discontinued as initially planned. If the 7-day dosing period of RLS-0071 and the timing for a change in the prophylactic regimen overlap, then the prophylactic regimen change should be deferred until after the 7-day RLS-0071 dosing period (see Section 7.5.3). Prophylactic medications must be continued as long as initially planned pre-study unless there is a clinical reason (e.g., safety) to taper the prophylactic regimen differently (see Section 7.5.3).

Ruxolitinib is typically continued for 6 months, and this study is 6 months in duration, so ruxolitinib should not be tapered during this study, unless clinically indicated.

Study Stopping Rules

Study stopping rules are defined in the study protocol (see Section 8.2).

A DSMB will be established for this study.

The criteria to determine dose escalation and safety review are defined in the protocol.

Statistics and Sample Size

The sample size was selected based on clinical considerations. The data will be summarized with descriptive statistics. A statistical section is included in Section 11. Additional details will be included in the statistical analysis plan (SAP) which will be finalized prior to database lock.

Endpoints*:

Co-Primary Endpoints:

- Safety and tolerability of RLS-0071 in the treatment of aGvHD.

- ORR of RLS-0071 at 28 days (note that addition of any acute GvHD therapy, including an increase in steroid dose >2 mg/kg MPE, will be considered a treatment failure).

Secondary Endpoints:

- CR at 7, 14, 28, 56, and 180 days.
- VGPR Rate at 7, 14, 28, 56, and 180 days.
- PR Rate at 7, 14, 28, 56, and 180 days.
- ORR at 7, 14, 56, and 180 days.
- ORR focused on lower GI response criteria only at 7, 14, 28, 56, and 180 days (for the overall study population and for the subset with lower GI aGvHD).
- CR, VGPR, and PR Rates focused on lower GI response criteria only at 7, 14, 28, 56, and 180 days (for the overall study population and for the subset with lower GI aGvHD).
- Incidence of refractoriness (to RLS-0071 +/- ruxolitinib) at Days 7, 14, 28, 56, and 180; refractoriness is defined as at least one of the following: aGvHD increasing in Stage in any organ or developing in a new organ after 3 days of RLS-0071; aGvHD that has not improved in Stage in >1 organ after 7 days of RLS-0071; initiation of additional aGvHD treatment (other than RLS-0071 +/- ruxolitinib); or for after Day 7, participants who progress during corticosteroid tapering before a 50% decrease in corticosteroids is achieved.
- Overall corticosteroid use on Days 7, 14, 28, 56, and 180.
- Initiation of additional or alternative treatment(s) for aGvHD (including an increase in steroid dose to >2 mg/kg methylprednisolone equivalent) to treat refractory aGvHD.
- Change or shift in Stage for lower GI aGvHD, liver aGvHD, skin aGvHD, or upper GI aGvHD from baseline to Days 7, 14, 28, 56, and 180.
- Attainment of Stage 0 or 1 lower GI aGvHD, liver aGvHD, skin aGvHD, or upper GI aGvHD at Days 7, 14, 28, 56, and 180.
- Change or shift in overall Grade of aGvHD (Table 11) from baseline to Days 7, 14, 28, 56, and 180.
- Loss of response, reduction in response, or incidence of flare in participants who had an initial response to RLS-0071 (with systemic corticosteroids and +/- ruxolitinib).
- Dose response (dose and duration) for the primary and secondary efficacy endpoints (across the dose regimens in the study).
- Overall survival, failure-free survival, and non-relapse mortality.
- Pharmacokinetics (based on sparse sampling for all participants and intensive sampling for a subset of participants) of RLS-0071.
- aGvHD participant outcomes: FACT-BMT, abdominal pain, volume/frequency of diarrhea, food tolerance and use of TPN, and duration of hospital stay.
- Change in MAGIC biomarkers (ST2 and REG3a) at Days 7, 14, and 28.

Exploratory Endpoints:

- PD plasma biomarkers (e.g., MPO concentration, MPO activity, NETosis marker cell-free DNA concentration, C1q concentration, sC5b-9 concentration, and functional complement assay: IC-MAC).
- MAGIC algorithm probability (MAP), and correlation of MAP with clinical efficacy and safety.

- Ability to taper corticosteroids: corticosteroid dosing discontinued or decreased to <2 mg/kg methylprednisolone or equivalent, with maintenance or improvement in aGvHD response at Days 14 and 28.

*The primary ORR endpoint and the corresponding response criteria definitions and secondary endpoints (CR, VGPR, and PR) are comprehensive and evaluate lower GI, upper GI, skin, and liver manifestations of aGvHD (Table 12). Secondary endpoints for ORR, and CR, VGPR, and PR are also included that focus on the lower GI manifestations of aGvHD (Table 13). Note that the endpoint timing is from the time of RLS-0071 initiation and not from the time of transplantation.

1.2 Schedule of Activities

Table 1: Schedule of Activities –7-Day Dosing Cohorts

**Study Phase**	**Screening a/ Baseline**		**Treatment Period b,c**							**Follow-up Period c**			
**Timepoint**	D0	D1<br>pre dose	D1<br>post dose	D2	D3	D4	D5	D6	D7	D14	D28	D56	D180/<br>Early<br>Termination
**Visit Window (days)**									±1	±2	±4	±7	±14
Informed consent	X												
Inclusion/exclusion	X												
Demographics	X												
Medical history	X												
Physical examination d	X			X	X	X	X	X	X	X	X	X	X
Vital signs e	X	X	X	X	X	X	X	X	X	X	X	X	X
Weight	X			X	X	X	X	X	X	X	X	X	X
Height f	X												
Safety Labs (chemistry, hematology, urinalysis) g	X			X	X				X	X	X	X	X
Safety Labs (coagulation) g	X								X	X	X		
Pregnancy Test (WOCBP only) h	X												
FSH i	X												
12-Lead ECG j	X			X					X				
Endoscopy (if performed SOC) +/- biopsy k	X												
PK blood samples l		X	X		X				X				
PD blood samples m		X	X		X				X	X			
ADA blood samples n		X									X		X
Stool output o	X			X	X	X	X	X	X	X	X	X	X
MAGIC stage (GI, skin, liver) p	X			X	X	X	X	X	X	X	X	X	X
Blood samples for ST2 & REG3a m		X							X	X	X		
Study drug (Q8H, 21 total doses) q			X	X	X	X	X	X	X				
Corticosteroid Administration r	X	X		X	X	X	X	X	X	X	X	X	X
Ruxolitinib Administration s	X	X		X	X	X	X	X	X	X	X	X	X
Concomitant medications	X	X		X	X	X	X	X	X	X	X	X	X
Participant outcomes t									X	X	X	X	X
Adverse events	X	X	X	X	X	X	X	X	X	X	X	X	X

Abbreviations: C_{trough} = the drug concentration observed prior to subsequent or final dosing; D = Day; EOI = end of infusion; ET = early termination; FU = follow-up; PD = pharmacodynamic; PK = pharmacokinetic; Q8H = every 8 hours; SoA = Schedule of Activities; SOC = standard of care; SC = Screening.

a. Every effort will be made to complete Screening procedures as quickly as possible.
b. Study intervention will be administered as soon as possible after enrollment.
c. If the participant has been discharged from the hospital, the visits may be performed in an outpatient setting.
d. A complete physical examination will be performed at Screening and on Day 180/ET. Abbreviated physical examinations will be performed at all other visits, unless otherwise determined by the Investigator. If the complete physical examination performed as part of Screening is within 6 hours of the infusion, another physical examination is not needed before the infusion. In this case, the Screening physical examination will serve as baseline. Vital signs (heart rate, respiration rate, blood pressure [systolic and diastolic], and temperature) will be recorded pre dose and 10 minutes post dose for each dose. Height will be recorded only at Screening. Clinical laboratory assessments will include standard safety parameters for chemistry, hematology, coagulation, and urinalysis (Table 17). If Screening laboratory assessments are performed within 6 hours before the infusion, they do not need to be repeated. In this case, the Screening values will serve as baseline. Women of childbearing potential will have a serum pregnancy test at Screening. An FSH level > 35 IU/L prior to or at Screening will be considered confirmatory in the absence of a clear postmenopausal history. Electrocardiograms will be performed at Screening, and on Day 2, and Day 7. Post-screening ECGs on Day 2 and Day 7 will be performed within 60 minutes after the end of the infusion. Endoscopy (if performed SOC), +/- biopsy at Screening and can also be performed at any other time during the study as indicated by the Investigator.
e. PK blood collections: **INTENSE PK: 3 participants each in Cohort 1a and Cohort 3 only:** D1 at pre-dose (any time prior to 1st dose), EOI (5 min post dose (± 3 min)), 1 hr post dose (± 10 min), 2 hr post dose (± 30 min), 4 hr post dose (± 30 min), C_{trough} (up to 120 min prior to next dose); D3 (around 7th, 8th, or 9th dose) at C_{trough} (up to 120 min prior to next dose); and D7 (around 21st dose) pre-dose (up to 120 minutes prior to 21st dose), at EOI (5 min post dose (± 3 min)), 1 hr post dose (± 10 min), 2 hr post dose (± 30 min), 4 hr post dose (± 30 min), 8hr post dose (± 120 min), and 24 hr post dose (±3hr). **All other participants will have SPARSE PK** collected at: D1 at pre-dose (any time prior to 1st dose), at EOI (5 min post dose (±3 min)), C_{trough} (up to 120 min prior to next dose); and D7 (around 21st dose) pre-dose (up to 120 minutes prior to 21st dose), at EOI (5 min post dose (± 3 min)), 2 hr post dose (± 30 min), and 24 hr post dose (±3hr). See Table 14 (Intense PK) and Table 15 (Sparse PK) for more details.
f. PD/MAGIC biomarker blood samples should be obtained once on the designated days at the time of collection of the safety labs or PK blood samples. First PD sample prior to first dose (baseline). Day 1 PD (second sample) sample should be obtained at the same time as the EOI (end of infusion) PK sample. Day 3, Day 7, and Day 14 PD blood samples should be drawn with safety labs.
g. First ADA sample prior to first dose (baseline). Samples will be collected on Day 28 for all participants. If the Day 28 result is positive, then a repeat sample will be collected at Day 180.
h. Stool volume, frequency, formation, and gross blood should be recorded daily.
i. For liver aGvHD, bilirubin must be measured as part of Staging. For Upper GI aGvHD, presence of nausea, vomiting, anorexia, and tolerance of oral or enteral feeds must be measured as part of Staging. For Lower GI aGvHD, stool output, frequency, presence of blood, abdominal pain, abdominal cramping, and ileus must be measured for Staging. For skin aGvHD, the percent body area of skin rash or presence of erythroderma with bullae or desquamation must be measured as part of Staging. See Table 10 for more details on the Staging of aGvHD and Table 11 for determination of overall Clinical Grade of aGvHD.
j. Study drug is administered Q8H for 21 total doses while in the hospital. If the participant is discharged, the study drug should be continued as Q8H dosing for the remainder of the assigned 7 day dosing period and can be administered as an outpatient in the clinic.
k. Corticosteroid should be tapered according to one of the tapering regimes. See Section 7.5.2 for specific details.
l. Ruxolitinib should be initiated within 48 hours of the start of RLS-0071. No tapering of dose should be done during the 180 day study period, unless clinically indicated.
m. Participant outcomes include FACT-BMT, abdominal pain, volume/frequency of diarrhea, food tolerance and use of TPN, and duration of hospital stay.

Table 2: Schedule of Activities –14-Day Dosing Cohorts

**Study Phase**	**Screening a / Baseline**		**Treatment Period b,c**														**Follow-up Period c**		
**Timepoint**	D0	D1 pre dose	D1 post dose	D2	D3	D4	D5	D6	D7	D8	D9	D10	D11	D12	D13	D14	D28	D56	D180/ ET
**Visit Window (days)**									±1							±2	±4	±7	±14
Informed consent	X																		
Inclusion/exclusion	X																		
Demographics	X																		
Medical history	X																		
Physical examination d	X			X	X	X	X	X	X	X	X	X	X	X	X	X	X	X	X
Vital signs e	X	X	X	X	X	X	X	X	X	X	X	X	X	X	X	X	X	X	X
Weight	X			X	X	X	X	X	X	X	X	X	X	X	X	X	X	X	X
Height f	X																		
Safety Labs (chemistry, hematology, urinalysis) g	X			X	X				X							X	X	X	X
Safety Labs (coagulation)g	X								X							X	X		
Pregnancy Test (WOCBP only) h	X																		
FSH i	X																		
12-Lead ECG j	X			X					X							X			
Endoscopy (if performed SOC) +/- biopsy k	X																		
PK blood samples l		X	X						X										
PD blood samples m		X	X		X				X							X			
ADA blood samples n		X															X		X
Stool output o	X			X	X	X	X	X	X	X	X	X	X	X	X	X	X	X	X
MAGIC stage (GI, skin, liver) p	X			X	X	X	X	X	X	X	X	X	X	X	X	X	X	X	X
Blood samples for ST2 & REG3a m		X							X							X	X		
Study drug (Q8H, 42 total doses) q			X	X	X	X	X	X	X	X	X	X	X	X	X	X			
Corticosteroid Administration r	X		X	X	X	X	X	X	X	X	X	X	X	X	X	X	X	X	X
Ruxolitinib Administration s	X		X	X	X	X	X	X	X	X	X	X	X	X	X	X	X	X	X
Concomitant medications	X	X		X	X	X	X	X	X	X	X	X	X	X	X	X	X	X	X
Participant outcomes t									X							X	X	X	X

**Study Phase**	**Screening a / Baseline**		**Treatment Period b,c**														**Follow-up Period c**		
**Timepoint**	D0	D1 pre dose	D1 post dose	D2	D3	D4	D5	D6	D7	D8	D9	D10	D11	D12	D13	D14	D28	D56	D180/ ET
**Visit Window (days)**									±1							±2	±4	±7	±14
Adverse events	X	X	X	X	X	X	X	X	X	X	X	X	X	X	X	X	X	X	X

Abbreviations: C_{trough} = the drug concentration observed prior to subsequent or final dosing; D = Day; EOI = end of infusion; ET = early termination; FU = follow-up; PD = pharmacodynamic; PK = pharmacokinetic; Q8H = every 8 hours; SoA = Schedule of Activities; SOC = standard of care; SC = Screening.

a. Every effort will be made to complete Screening procedures as quickly as possible.
b. Study intervention will be administered as soon as possible after enrollment.
c. If the participant has been discharged from the hospital, the visits may be performed in an outpatient setting.
d. A complete physical examination will be performed at Screening and on Day 180/ET. Abbreviated physical examinations will be performed at all other visits, unless otherwise determined by the Investigator. If the complete physical examination performed as part of Screening is within 6 hours of the infusion, another physical examination is not needed before the infusion. In this case, the Screening physical examination will serve as baseline. Vital signs (heart rate, respiration rate, blood pressure [systolic and diastolic], and temperature) will be recorded pre dose and 10 minutes post dose for each dose. Height will be recorded only at Screening. Clinical laboratory assessments will include standard safety parameters for chemistry, hematology, coagulation, and urinalysis (Table 17). If Screening laboratory assessments are performed within 6 hours before the infusion, they do not need to be repeated. In this case, the Screening values will serve as baseline. Women of childbearing potential will have a serum pregnancy test at Screening. An FSH level > 35 IU/L prior to or at Screening will be considered confirmatory in the absence of a clear postmenopausal history. Electrocardiograms will be performed at Screening, and on Day 2, Day 7, and Day 14. Post-screening ECGs will be performed within 60 minutes after the end of the infusion. Endoscopy, if performed SOC, +/- biopsy at Screening and can also be performed at any other time during the study as indicated by the Investigator
e. All 14-day dosing participants will have SPARSE PK collected: D1 at pre-dose (any time prior to 1st dose), EOI (5 min post dose ±3 min), C_{trough} (up to 120 min prior to next dose); and D7 (around 21st dose) at pre-dose (up to 120 minutes prior to 21st dose), EOI (5 min post dose ±3 min)), 2 hr post dose (±30 min), and 24 hr post dose (±3hr). See Table 15 (Sparse PK) for more details.
f. PD/MAGIC biomarker blood samples to be obtained once on the designated days at the time of collection of the safety labs or PK blood samples. First PD sample prior to first dose (baseline). Day 1 PD (second sample) sample should be obtained at the same time as the EOI (end of infusion) PK sample. Day 3, Day 7, and Day 14 PD blood sample to be drawn with safety labs.
g. First ADA sample prior to first dose (baseline). Samples will be collected on Day 28 for all participants. If the Day 28 result is positive, then a repeat sample will be collected at Day 180.
h. Stool volume, frequency, formation, and gross blood should be recorded daily.
i. For liver aGvHD, bilirubin must be measured as part of Staging. For Upper GI aGvHD, presence of nausea, vomiting, anorexia, and tolerance of oral or enteral feeds must be measured as part of Staging. For Lower GI aGvHD, stool output, frequency, presence of blood, abdominal pain, abdominal cramping, and ileus must be measured for Staging. For skin aGvHD, the percent body area of skin rash or presence of erythroderma with bullae or desquamation must be measured as part of Staging. See Table 10 for more details on the Staging of aGvHD and Table 11 for determination of overall Clinical Grade of aGvHD.
j. Study drug is administered Q8H for up to 42 total doses while in the hospital. If the participant is discharged, the study drug should be continued as Q8H dosing for the remainder of the assigned 14 day dosing period and can be administered as an outpatient in the clinic. Participants in Cohort 2a or Cohort 3 who improve and are able to leave the hospital any time between 7 to 13 days of treatment with RLS-0071 and who are rolled over into Cohort 4 or Cohort 5 will receive RLS-0071 QD following discharge to outpatient to completion of Day 14 (approximate range of 28 - 42 total doses of RLS-0071).
k. Corticosteroid should be tapered according to one of the tapering regimes See Section 7.5.2 for specific details.
l. Ruxolitinib should be initiated within 48 hours of the start of RLS-0071. No tapering of dose should be done during the 180 day study period, unless clinically indicated.
m. Participant outcomes include FACT-BMT, abdominal pain, volume/frequency of diarrhea, food tolerance and use of TPN, and duration of hospital stay.

2. Table of Contents for the Clinical Study Protocol

Compliance Statement 1
Signature Page 2
Investigator Agreement 3
1. Study Overview 4
1.1 Synopsis...4
1.2 Schedule of Activities 17
2. Table of Contents for the Clinical Study Protocol 21
List of Tables 26
List of Figures..26
List of Abbreviations 27
3. Introduction...29
3.1 Background 29
3.1.1 Acute Graft-versus-Host Disease...29
3.1.2 Unmet Medical Need..30
3.2 Study Rationale 31
3.3 Risks/Benefit Assessment 32
4. Objective and Endpoints...33
4.1 Objectives..33
4.1.1 Primary Objectives 33
4.1.2 Secondary Objectives...33
4.1.3 Exploratory Objectives...33
4.2 Endpoints* 34
4.2.1 Primary Endpoints..34
4.2.2 Secondary Endpoints 34
4.2.3 Exploratory Endpoints 35
5. Study Design...36
5.1 Overall Design 36
5.2 Scientific Rationale for Study Design...41
5.3 Justification for Dose 41
5.4 End of Study Definition 41

5.5 Duration of Participation 41
6. Selection and Withdrawal of Participants 42
6.1 Participant Inclusion Criteria 42
6.2 Participant Exclusion Criteria 43
6.3 Screen Failures 44
6.4 Replacement of Participants 44
7. Treatment of Participants 46
7.1 Treatment to be Administered 46
7.2 Preparation/Handling/Storage/Accountability 49
7.3 Procedures for Monitoring Participant Compliance 49
7.4 Medication(s) Treatment(s) Permitted and Not Permitted 49
7.4.1 Corticosteroids 49
7.4.2 Ruxolitinib 50
7.4.3 Treatments for aGvHD 50
7.4.4 Treatments for Conditions other than aGvHD 50
7.4.5 Contraception 51
7.4.6 Medications to be used with Caution 51
7.4.6.1 CYP2C19 Substrates 51
7.4.6.2 CYP3A4 Inhibitors or Fluconazole 51
7.4.7 Prohibited Medications 51
7.5 Timing and Order of Medication Taper 52
7.5.1 RLS-0071 52
7.5.2 Corticosteroids 52
7.5.3 Prophylactic Medication 53
7.5.4 Ruxolitinib 53
7.6 Dose Modification 54
7.6.1 Dose Modification in Case of Infusion-Related Reaction 54
7.6.2 Dose Modification in Case of Moderate Renal Impairment 55
7.6.3 Dose Modification in Case of Concomitant Use with Strong CYP3A4 Inhibitors or Fluconazole 56
7.7 Intervention After the End of the Study 56
8. Discontinuation of Study Intervention and Participant Discontinuation/Withdrawal 57

8.1 Discontinuation of Study Intervention 57
8.2 Dose Limiting Toxicities and Study Stopping Rules 57
8.2.1 AEs that are Generally Considered Related to Another Medication or the Underlying Disease 57
8.2.1.1 Expected Adverse Events due to Ruxolitinib 57
8.2.1.2 Expected aGvHD Manifestations 57
8.2.2 Dose Escalation and Study Stopping Rules 58
8.3 Decision to Proceed with Enrollment in the Next Cohort or Dose Expansion 58
8.3.1 Dose Expansion 58
8.4 Participant Discontinuation/Withdrawal from the Study 58
8.5 Lost to Follow-Up 59
8.6 Study Discontinuation 59
9. Study Assessments and Procedures 61
9.1 Study Procedures 61
9.1.1 Screening/Baseline Procedures (Day 0) 61
9.1.2 Study Intervention Administration (7-day dosing Cohorts) 62
9.1.3 Study Intervention Administration (14-day dosing Cohorts) 63
9.1.4 Follow-Up Visits 65
9.1.5 Early Termination 65
9.1.6 Unscheduled Assessments 65
9.2 Assessment of Efficacy 65
9.2.1 Response to RLS-0071 65
9.2.2 Taper of Corticosteroids 66
9.2.3 Use of Rescue Medications for Refractory Disease 67
9.2.4 Change in Stage of aGvHD 67
9.2.5 Survival 67
9.2.6 MAGIC Biomarkers 67
9.2.7 Pharmacodynamic Biomarkers 67
9.2.8 MAGIC Algorithm Probability (MAP) 67
9.2.9 Participant Outcomes 68
9.2.9.1 Functional Assessment of Cancer Therapy - Bone Marrow Transplantation (FACT-BMT) 68
9.2.9.2 Abdominal Pain 68

9.2.9.3 Volume/Frequency of Diarrhea 68
9.2.9.4 Food Tolerance and Use of TPN 68
9.2.9.5 Duration of Hospital Stay 68
9.3 Assessments of Pharmacokinetics 68
9.3.1 Blood Sample Collection 68
9.3.2 Sample Processing 69
9.3.3 Sample Analysis and Analytical Methods 70
9.4 Assessment of Immunogenicity 71
9.5 Assessment of Safety 71
9.5.1 Safety Parameters 71
9.5.2 Physical Examination 71
9.5.2.1 Vital Signs 72
9.5.2.2 Electrocardiogram 72
9.5.2.3 Stool Output 72
9.5.2.4 Clinical Safety Laboratory Assessments 72
10. Adverse Events and Serious Adverse Events 74
10.1 Definition of Adverse Events (AEs) 74
10.2 Definition of Serious Adverse Event 75
10.2.1 Notification of Serious Adverse Event 76
10.3 Recording Adverse Events 76
10.4 Severity of an AE/SAE 76
10.5 Causal Relationship of an AE/SAE 77
10.6 Action Taken with Investigational Products 78
10.7 Outcome 79
10.8 Follow-up of Adverse Events and Serious Adverse Events 79
10.9 Adverse Events that are Expected 79
10.9.1 Expected Adverse Events due to RLS-0071 79
10.9.2 Expected Adverse Events due to Ruxolitinib 80
10.9.3 Expected aGvHD Manifestations 80
10.10 Adverse Events of Special Interest 80
10.11 Pregnancy 80
11. Statistics 81

11.1 Sample Size Determination 81
11.2 Populations for Analysis 81
11.3 Statistical Analyses 81
11.3.1 Primary Endpoints 81
11.3.2 Secondary Endpoints 82
11.3.3 Pharmacokinetic Endpoints 82
11.3.4 Exploratory Endpoints 82
11.4 Interim Analysis 82
11.5 Data Safety Monitoring Board (DSMB) 82
12. Direct Access to Source Data/Documents 84
13. Quality Assurance and Quality Control 85
14. Ethics Description and Ethical Considerations 86
14.1 Informed Consent 86
15. Data Handling and Recordkeeping 88
15.1 Dissemination of Clinical Study Data 88
16. Financial Statement 89
17. Publication Policy 90
18. References 91
Appendix 1. Summary of Changes 93
Amendment 1 93
Amendment 2 95
Amendment 3 96
Amendment 4 97
Amendment 5 97
Amendment 6 98
Amendment 7 99
Amendment 8 99

List of Tables

Table 1: Schedule of Activities –7-Day Dosing Cohorts 17
Table 2: Schedule of Activities –14-Day Dosing Cohorts 19
Table 3: Karnofsky Performance Status Scale Definitions Rating (%) Criteria 45
Table 4: Dose Escalation Study Interventions (7-day dosing Cohorts) 47
Table 5: Dose Escalation Study Interventions (14-day dosing Cohorts) 48
Table 6: Management of Infusion Related Reactions 55
Table 7: Management of Subsequent Infusion Related Reactions after Re-challenge 55
Table 8: Dose Modifications for Ruxolitinib for Concomitant Use with Renal Impairment 55
Table 9: Dose Modifications for Ruxolitinib for Concomitant Use with Strong CYP3A4 Inhibitors or Fluconazole 56
Table 10: GVHD Target Organ Staging (from MAGIC Criteria) 62
Table 11: Overall aGvHD Clinical Grade (based upon most severe target organ involvement) MAGIC Criteria 62
Table 12: Response Criteria (GI, skin, and liver) 66
Table 13: Response Criteria: Lower GI aGvHD 66
Table 14: Intense PK Blood Sampling Schedule (3 Participants each in Cohort 1a and Cohort 3) 69
Table 15: Sparse PK Blood Sampling Schedule (All Other Participants) 69
Table 16: Pharmacokinetic Parameters Derived for RLS-0071 Concentrations 70
Table 17: Clinical Laboratory Assessments 73
Table 18: Causality Categories 78

List of Figures

Figure 1: Treatment Cohorts 40
Figure 2: Corticosteroid Taper 53

List of Abbreviations

Abbreviation	Definition
ADA	Anti-drug antibodies
AE	Adverse event
aGvHD	Acute graft versus host disease
allo-HSCT	Allogeneic hematopoietic stem cell transplantation
Cdiff	*Clostridioides difficile*
CFR	Code of Federal Regulations
CH50	Complement activity assay
CIOMS	Council for International Organizations of Medical Sciences
CKD	Chronic kidney disease
CMV	Cytomegalovirus
CR	Complete response
CRO	Contract Research Organization
CRP	C-reactive protein
CSR	Clinical study report
CTCAE	Common Terminology Criteria for Adverse Events
C_{trough}	Trough concentrations prior to dose administration
CV	Coefficient of variation
DLT	Dose limiting toxicity
DMARD	Disease-modifying antirheumatic drugs
DNA	Deoxyribonucleic acid
DSMB	Data Safety Monitoring Board
ECG	Electrocardiogram
eCRF	Electronic case report form
EDC	Electronic data capture
eGFR	Estimated glomerular filtration rate
EOI	End of infusion
ET	Early Termination
FACT-BMT	Functional Assessment of Cancer Therapy - Bone Marrow Transplantation
FDA	Food and Drug Administration
FSH	Follicle-stimulating hormone
GCP	Good Clinical Practice
GI	Gastrointestinal
HBV	Hepatitis B virus
HEENT	Head, eyes, ears, nose, throat
HIPAA	Health Insurance Portability and Accountability Act
HIV	Human immunodeficiency virus
ICF	Informed consent form
ICH	International Council for Harmonisation
IEC	Independent Ethics Committee
IL	Interleukin
IP	Investigational product
IRB	Institutional Review Board
ITT	Intent to treat

Abbreviation	Definition
IV	Intravenous
LPS	Lipopolysaccharide
MAD	Multiple ascending dose
MAGIC	Mount Sinai Acute GVHD International Consortium
MAP	MAGIC algorithm probability
MBL	Mannose biding lectin
MHC	Major histocompatibility complex
MPO	Myeloperoxidase
MTD	Maximum tolerated dose
NET	Neutrophil extracellular trap
NETosis	Formation of neutrophil extracellular traps
NIH	National Institutes of Health
ORR	Overall response rate
PD	Pharmacodynamic
PEG	Polyethylene glycol
PK	Pharmacokinetics
PR	Partial Response
Q8H	Every 8 hours
QD	Once daily
REG3α	Regenerating islet-derived 3-alpha
SAD	Single ascending dose
SAE	Serious adverse event
SAP	Statistical analysis plan
SD	Standard deviation
SoA	Schedule of activities
SOP	Standard operating procedure
ST-2	Suppressor of tumorigenesis 2
TEAE	Treatment-emergent adverse event
TPN	Total parenteral nutrition
VAS	Visual analog scale
VGPR	Very good partial response
WOCBP	Woman of childbearing potential

3. Introduction

ReAlta Life Sciences, Inc (Sponsor) has developed a peptide platform, termed "EPICC," with dual targeting mechanisms of action. This platform presents a novel opportunity to modify disease progression through moderation of the most robust components of both humoral (complement system) and cellular (neutrophils) components of innate immunity.

EPICC molecules rapidly and dose dependently inhibit C1 and mannose binding lectin (MBL), the earliest initiators of the classical and lectin complement pathway. Inhibition is via transformational changes of serine proteases at C1 and MBL.

- Selective upstream targeting of the complement system increases selectivity, efficacy, and reduces adverse events.
- EPICC molecules do not interfere with C1q homeostatic functions and preserve the alternative pathway, maintaining the body's immunosurveillance capacity.
- EPICC molecules' small size and function lead to excellent tissue penetration.

In addition to complement modulation, the EPICC platform acts as a potent inhibitor of innate, neutrophil-mediated, inflammatory processes, including inhibition of myeloperoxidase (MPO) activity and the formation of neutrophil extracellular traps (NETosis).

- Blocks MPO activity that generates hypochlorous acid and host tissue injury.
- Inhibits NETosis, a neutrophil death pathway contributing to host tissue damage.
- Exhibits antioxidative properties and neutralizes free radicals in the body.

The lead compound, previously referenced as PIC1, PA-dPEG24, PIC1dPEG, and PIC1-dPEG24, is hereto referred to as RLS-0071. The product is composed of the peptide sequence IALILEPICCQERAA with a monodisperse polyethylene glycol (PEG) tail.

3.1 Background

3.1.1 Acute Graft-versus-Host Disease

The proposed indication for RLS-0071 is for the treatment of acute Graft-versus-Host Disease (aGvHD).

Acute GvHD remains a common complication for patients receiving allogeneic hematopoietic transplantation. For patients receiving allogeneic hematopoietic transplantation approximately 60% will develop aGvHD of grades II – IV, moderate to very severe (Pasquini and Zhu, 2014). The disease process of aGvHD is characterized by a complex immune-mediated attack on host tissues and inflammation that can lead to morbidity and has been reported to be associated with a 16% mortality rate, more than 3-fold higher than allogeneic hematopoietic transplantation patients without aGvHD (Yu et al., 2019). The most severe form of aGvHD involves the intestines and leads to pain, diarrhea, malabsorption and malnutrition (Naymagon et al., 2017).

The pathogenesis of aGvHD is multifaceted, but the incomplete response or unresponsiveness of most patients to corticosteroids strongly suggests that other factors than lymphocytes must play an important role. To date, the general perspective of how GvHD develops is that antigen-presenting cells expressing foreign major histocompatibility complex (MHC) and minor histocompatibility antigens activate donor-derived T-cells in lymphatic and end organs. To this end, therapeutic targets have focused on moderating the alloreactive T-cell activation and

expansion such as calcineurin inhibitors, antimetabolites (methotrexate and mycophenolate) (Storb, 1986), post-transplant cyclophosphamide (Kanakry, 2014) or anti-thymocyte globulin (Finke, 2009; Kröger, 2016). Recent work suggests that much of the pathogenesis is driven by the conditioning therapy causing endothelial injury followed by neutrophil recruitment and activation, NETosis and complement activation (Gloude et al., 2017). The importance of these mechanisms have been demonstrated in patient biomarker studies (Qiao et al., 2015) and animal models (Hulsdunker et al., 2018) of aGvHD. The unique dual-targeting mechanisms of RLS-0071 to inhibit complement activation and neutrophil effectors, MPO and NETosis, are likely -ideal for addressing these critical aspects of aGvHD pathogenesis.

Understanding the role of neutrophils in acute GvHD has been significantly enhanced by the animal model work of Dr. Robert Zeiser at the University of Freiburg. In a standard mouse model of transplantation conditioning with total body irradiation, Dr. Zeiser's group showed that post insult neutrophils localize adjacent to intestinal crypts in the ilium and then migrate to the mesenteric lymph nodes. In the mesenteric lymph nodes neutrophils closely associate with transplanted T cells ($CD45.1^{+}$) and can present antigens in an MHC-II-dependent manner. These findings corroborate those of other investigators demonstrating that the conditioning regimen for allogeneic hematopoietic transplantation initiates a neutrophil-driven process to sensitize donor T-cells contributing to the pathogenesis of GvHD.

3.1.2 Unmet Medical Need

Approximately 45,000 allogeneic hematopoietic transplants were performed in the U.S. between 2016 and 2020 (HRSA, 2022). As noted above, over half of those transplanted patients will develop moderate to very severe aGvHD. Prophylactic therapy for GvHD is standardly administered to patients. These often include calcineurin inhibitors and/or other medicines. Orencia (abatacept), has been recently approved for prevention of aGvHD for a select group of patients, but it has limitations and is not widely used. Despite prophylactic therapy, many participants then develop aGvHD, often beginning around 30 days following transplantation. Corticosteroids, which are administered systemically for treatment of moderate to severe aGvHD, are the cornerstone and are typically the only primary treatment for aGvHD. Many patients (approximately half) fail corticosteroid treatment and their aGvHD either persists or worsens which leads to requirement for secondary treatment. Jakafi (ruxolitinib) has been recently approved for the treatment of steroid-refractory aGvHD (i.e., secondary treatment), but patients must first demonstrate failure after at least 3-7 days of corticosteroid treatment. Additionally, Jakafi (ruxolitinib) has a significantly adverse side effect profile dissuading usage by physicians. Currently, systemic methylprednisolone remains the mainstay of treatment for aGvHD patients with grade II – IV (Saad et al., 2020) despite limited effectiveness and a very problematic side effect profile at the high doses given for aGvHD. Unfortunately, as described above approximately 50% or more of intestinal aGvHD patients will be unresponsive to corticosteroids (Naymagon et al., 2017). The mortality rate in these patients is unacceptably high. There is a clear opportunity to provide an additional therapy at the onset of aGvHD (steroid refractory for this study, or potentially earlier for future studies) that addresses other mechanisms contributing to the disease process in order to provide effective treatment for more patients earlier in their disease process. RLS-0071 is therefore being developed as a treatment for aGvHD on top of systemic corticosteroids, and in this secondary treatment study also +/- ruxolitinib.

Overall, the unmet medical need in aGvHD remains high and the patient outcomes poor. New therapies are needed to address other important aspects of aGvHD pathogenesis beyond

lymphocytes, such as neutrophil-initiated inflammation post conditioning, NETosis and complement activation. Orthogonal interventions directed towards these currently unaddressed mechanisms of action have tremendous potential to treat aGvHD patients early and quickly moderate the disease process.

3.2 Study Rationale

ReAlta Life Sciences, Inc (Sponsor) has developed a new class of peptides that specifically inhibit multiple inflammatory pathways including the classical and lectin pathways of complement, MPO and neutrophil extracellular trap (NET) formation. Preliminary preclinical data in a standard mouse model of aGvHD showed that RLS-0071 greatly increased survival and significantly decreased end-organ damage, this is further described in the Investigator Brochure. RLS-0071 has completed a healthy human volunteer Phase 1 clinical trial that defined the PK parameters in healthy adult humans and demonstrated no significant toxicities for any of the doses tested. RLS-0071 has also completed a Phase 1b clinical trial in Germany of healthy volunteers inhaling LPS simulating neutrophil-driven disease process. This trial demonstrated no toxicities associated with RLS-0071 and showed clinically meaningful pharmacodynamic effects measured by a reduction in sputum lung neutrophils, myeloperoxidase, NETosis markers, complement, and cytokine levels.

The ability of RLS-0071 to inhibit both complement and neutrophil mediated inflammation suggests that this drug may have efficacy for preventing or treating and reversing the pathogenesis of aGvHD and the intestinal manifestations in particular.

The population for this study is patients who had received allo-HSCT, who have aGvHD, and who have become refractory to systemic corticosteroids and are hospitalized. The main goals of this study are to evaluate the safety/tolerability and Overall Response Rate (ORR) of RLS-0071 in steroid refractory aGvHD. Additional goals include evaluation of dose, pharmacokinetics, pharmacodynamics, and other measures of efficacy.

The primary efficacy endpoint is ORR at 28 days. ORR (specifically at 28 days) is an accepted primary endpoint for the treatment of aGvHD, and comprises complete response, very good partial response, and partial response, all of which are clinically meaningful assessments. The underlying response criteria (CR, VGPR, and PR) that comprise the ORR assessment evaluate GI, skin, and liver manifestations of aGvHD. The FDA-approved secondary treatment Jakafi (ruxolitinib) timed their ORR primary endpoint at 28 days in the steroid-refractory patient population. RLS-0071 is fast-acting and is being developed for treatment of aGvHD, with the goal to enable rapid disease control. Full GI mucosal healing and a full clinical response may take longer than 7 days, and the clinical study design therefore includes dosing regimens of 7 and 14 days duration. Patients will be evaluated at a range of timepoints including 7 and 14 days for rapid response, 28 days, as well as 56 and 180 days for continued improvement and durability of response. Considering a particular opportunity for GI GvHD, the secondary endpoints include ORR and CR, VGPR, and PR that focus on the lower GI manifestations of aGvHD at various timepoints.

In addition to the ORR primary endpoint and a set of other clinical efficacy endpoints, the study includes assessment of the MAGIC biomarkers ST2 and REG3α. The Mount Sinai Acute GVHD International Consortium (MAGIC) algorithm probability (MAP) is derived from these 2 serum biomarkers and measures damage to crypts in the gastrointestinal tract during GVHD. Changes in MAP after treatment suggest that the algorithm can be used as a response biomarker and

correlated with clinical outcomes. Additionally, combination of the MAP with clinical endpoints may further predict response. The MAGIC biomarkers should provide further insight into the early clinical response to aGvHD treatment.

The completed phase 1 (RLS-0071-101) healthy volunteer SAD/MAD PK study and the Phase 1b inhaled LPS proof of mechanism study (RLS-0071-103) included intensive PK sampling, which generated data at the doses being studied in this aGvHD clinical study. Accordingly, this aGvHD clinical study collects sparse PK data on all study participants to confirm and extend the earlier findings in an aGvHD patient population, as well as intense PK on a subset of participants receiving the 10 mg/kg or 40 mg/kg doses to evaluate whether the physiology of aGvHD affects how RLS-0071 is eliminated and metabolized.

3.3 Risks/Benefit Assessment

Allogeneic hematopoietic transplants remain a common therapy for hematogenous cancers but continue to carry a high risk for developing aGvHD, over half of which will be between moderate severity and extremely severe. Moreover, over half of these patients will become corticosteroid-resistant, the mainstay of treatment. Thus, outcomes for many aGvHD patients remain poor particularly for those that develop intestinal aGvHD, the form which carries the highest morbidity and mortality.

RLS-0071 will be added on top of corticosteroid standard of care +/- ruxolitinib in participants with steroid-refractory aGvHD. When used in this way, RLS-0071 is intended to help address the unmet need and achieve faster and better disease control. If the therapy is effective, disease signs and symptoms would reduce or become eliminated, with fewer participants progressing to the need for additional secondary or tertiary treatments. If RLS-0071 is not effective, then the option is preserved to add or switch to other therapies used to treat refractory disease.

RLS-0071 has been shown to be safe with no demonstrable safety signal in adult humans at doses up to 120 mg/kg in two Phase 1 clinical trials involving over 80 participants, total. Thus, the safe therapeutic window appears to be wide and encompassing the doses proposed for treating aGvHD. RLS-0071 mechanisms of action target the vital aspects of complement activation, myeloperoxidase activity and NETosis that drive intestinal aGvHD pathogenesis and are unaddressed by current standard of care therapies. Additionally, RLS-0071 preclinical data in a range of animal models, including intestinal necrosis and LPS-mediated acute lung injury, provide a strong rationale for the potential for RLS-0071 to lessen the severity of aGvHD. From discussions with key opinion leaders for aGvHD it is clear there is consensus for finding better treatment options than the current standard of care. Thus, RLS-0071 offers tremendous potential for decreasing the morbidity and mortality associated with aGvHD with a low risk of adverse effects from the medication.

4. Objective and Endpoints

4.1 Objectives

4.1.1 Primary Objectives

- Demonstrate safety and tolerability of RLS-0071 in the treatment of aGvHD.
- Determine Overall Response Rate (ORR) of RLS-0071 at 28 days.

4.1.2 Secondary Objectives

- Determine Complete Response (CR) Rate, Very Good Partial Response (VGPR) Rate, and Partial Response (PR) Rate at 7, 14, 28, 56, and 180 days.
- Determine ORR at 7, 14, 56, and 180 days.
- Determine ORR, and CR Rate, VGPR Rate, and PR Rate at 7, 14, 28, 56, and 180 days based on lower gastrointestinal (GI) response only (overall study population and for the subset with lower GI aGvHD).
- Assess refractoriness to RLS-0071 +/- ruxolitinib.
- Determine need for initiation of additional or alternative treatments for aGvHD.
- Evaluate change in Stage and attainment of Stage 0 or 1 for lower GI aGvHD, liver aGvHD, skin aGvHD, and upper GI aGvHD, and overall aGvHD Grade from baseline to Days 7, 14, 28, 56, and 180.
- Assess loss of response, reduction in response, or occurrence of flare in participants who had an initial response to RLS-0071 (with systemic corticosteroids and +/- ruxolitinib).
- Evaluate dose response (dose and duration) for the primary and secondary efficacy endpoints.
- Characterize overall survival, failure-free survival, and non-relapse mortality.
- Characterize Pharmacokinetics (based on sparse sampling for all participants and intensive sampling for a subset of participants) of RLS-0071.
- Evaluate aGvHD participant outcomes: FACT-BMT, abdominal pain, volume/frequency of diarrhea, food tolerance and use of total parenteral nutrition (TPN), and duration of hospital stay.
- Evaluate change in Mount Sini Acute GVHD International Consortium (MAGIC) biomarkers (ST2 and REG3α) from Baseline to Days 7, 14, and 28.

4.1.3 Exploratory Objectives

- Evaluate PD biomarkers (e.g., for assessment of aGvHD and effects of RLS-0071).
- Calculate MAGIC algorithm probability (MAP) and assess correlation of MAP with clinical efficacy and safety.
- Assess ability to taper corticosteroids and characterize overall corticosteroid use at Days 7, 14, 28, 56, and 180.

4.2 Endpoints*

4.2.1 Primary Endpoints

- Safety and tolerability of RLS-0071 in the treatment of aGvHD.
- ORR of RLS-0071 at 28 days (note that addition of any acute GvHD therapy, including an increase in steroid dose to >2 mg/kg MPE, will be considered a treatment failure).

*The primary ORR endpoint and the corresponding response criteria definitions and secondary endpoints (CR, VGPR, and PR) are comprehensive and evaluate lower GI, upper GI, skin, and liver manifestations of aGvHD (Table 12). Secondary endpoints for ORR, and CR, VGPR, and PR are also included that focus on the lower GI manifestations of aGvHD (Table 13). Note that the endpoint timing is from the time of RLS-0071 initiation and not from the time of transplantation.

4.2.2 Secondary Endpoints

- CR Rate at 7, 14, 28, 56, and 180 days.
- VGPR Rate at 7, 14, 28, 56, and 180 days.
- PR Rate at 7, 14, 28, 56, and 180 days.
- ORR at 7, 14, 56, and 180 days.
- ORR focused on lower GI response criteria only at 7, 14, 28, 56, and 180 days (for the overall study population and for the subset with lower GI aGvHD).
- CR, VGPR, and PR Rates focused on lower GI response criteria only at 7, 14, 28, 56, and 180 days (for the overall study population and for the subset with lower GI aGvHD).
- Incidence of refractoriness (to RLS-0071 +/- ruxolitinib) at Days 7, 14, 28, 56, and 180; refractoriness is defined as at least one of the following: aGvHD increasing in Stage in any organ or developing in a new organ after 3 days of RLS-0071; aGvHD that has not improved in Stage in >1 organ after 7 days of RLS-0071; initiation of additional aGvHD treatment (other than RLS-0071 +/- ruxolitinib); or for after Day 7, participants who progress during corticosteroid tapering before a 50% decrease in corticosteroids is achieved.
- Overall corticosteroid use at Days 7, 14, 28, 56, and 180.
- Initiation of additional or alternative treatment(s) for aGvHD (including an increase in steroid dose to >2 mg/kg methylprednisolone equivalent) to treat refractory aGvHD.
- Change or shift in Stage for lower GI aGvHD, liver aGvHD, skin aGvHD, or upper GI aGvHD from baseline to Days 7, 14, 28, 56, and 180.
- Attainment of Stage 0 or 1 lower GI aGvHD, liver aGvHD, skin aGvHD, or upper GI aGvHD at Days 7, 14, 28, 56, and 180.
- Change or shift in overall Grade of aGvHD (Table 11) from baseline to Days 7, 14, 28, 56, and 180.
- Loss of response, reduction in response, or incidence of flare in participants who had an initial response to RLS-0071 (with systemic corticosteroids and +/- ruxolitinib).
- Dose response (dose and duration) for the primary and secondary efficacy endpoints (across the dose regimens in the study).

- Overall survival, failure-free survival, and non-relapse mortality.
- Pharmacokinetics (based on sparse sampling for all participants and intensive sampling for a subset of participants) of RLS-0071.
- aGvHD participant outcomes: FACT-BMT, abdominal pain, volume/frequency of diarrhea, food tolerance and use of TPN, and duration of hospital stay.
- Change in MAGIC biomarkers (ST2 and REG3α) from Baseline to Days 7, 14, and 28.

4.2.3 Exploratory Endpoints

- PD plasma biomarkers (e.g., MPO concentration, MPO activity, NETosis marker cell-free DNA concentration, C1q concentration, sC5b-9 concentration, and functional complement assay: IC-MAC).
- MAGIC algorithm probability (MAP), and correlation of MAP with clinical efficacy and safety.
- Markers of acute inflammatory response, such as C-reactive protein (CRP) and selected cytokine levels (e.g., interleukin (IL)-1β, IL-8) may also be evaluated.
- Ability to taper corticosteroids after Day 7: corticosteroid dosing discontinued or decreased to <2 mg/kg methylprednisolone or equivalent, with maintenance or improvement in aGvHD response.

5. Study Design

5.1 Overall Design

Phase 2 Open Label Prospective Dose-Ranging Escalation and Expansion Trial to Evaluate Safety, Tolerability, Pharmacokinetics, Pharmacodynamics, Dosing, and Efficacy of RLS-0071 for the secondary treatment of acute Graft-versus-Host Disease (aGvHD) in hospitalized participants who are steroid-refractory. Participants will be enrolled after meeting criteria for steroid-refractory aGvHD, and RLS-0071 will be initiated. Ruxolitinib will also be initiated unless ruxolitinib is contraindicated or considered inappropriate treatment for a specific study participant. No other (secondary) aGvHD treatments are permitted (unless aGvHD progresses further or does not improve after 7 days). Systemic corticosteroids and GvHD prophylactic medications should be continued according to the schedules detailed in Section 7.5.

RLS-0071 will be administered for 7 or 14 days according to the assigned dose group. RLS-0071 should ideally be administered simultaneously to the time of ruxolitinib, but the time interval between ruxolitinib and RLS-0071 initiations must be less than 48 hours of each other. Note that as part of the study design, when ruxolitinib is planned from the time of study initiation and is administered after RLS-0071 initiation but within 48 hours, then the "addition" of ruxolitinib will not be considered as an RLS-0071 treatment failure. The systemic corticosteroid dose should be ≥2 mg/kg methylprednisolone or equivalent for initial treatment of aGvHD prior to enrollment. The parameters for tapering are listed in Section 7.5.2.

Adults or adolescents (>12 years old) hospitalized with steroid refractory aGvHD after allogeneic hematopoietic stem cell transplantation (allo-HSCT) with any conditioning regimen will be enrolled (see inclusion criteria for specific population definition). aGvHD will be characterized according to the MAGIC criteria as Grade II, III, or IV, defined as:

- Grade II: Stage 3 rash and/or stage 1 liver and/or stage 1 upper GI and/or stage 1 lower GI
- Grade III: Stage 2–3 liver and/or stage 2–3 lower GI, with stage 0–3 skin and/or stage 0-1 upper GI
- Grade IV: Stage 4 skin, liver, or lower GI involvement, with stage 0–1 upper GI

Note: Patients with Stage 3 or 4 liver are excluded from participation in the study

Consistent with clinical practice, participants will be enrolled, and RLS-0071 treatment started based on aGvHD clinical criteria, and all the key response criteria will also be based on clinical endpoints. The early intervention with RLS-0071 based on clinical criteria underscores the importance of intervening quickly to treat aGvHD, to avoid disease worsening, and to optimize the likelihood of improvement at the time of steroid refractoriness and avoid the need for third-line treatment.

A lower GI endoscopy with biopsy is recommended (as part of standard of care) for participants with signs and symptoms of lower GI aGvHD, as long as the endoscopy (typically a sigmoidoscopy) +/- biopsy are anticipated to be safe and feasible for the participant. If the endoscopy and biopsy definitively rule out a diagnosis of lower GI aGvHD for participants who would only qualify for the study based on lower GI aGvHD (i.e., they do not have Grade >II aGvHD based on skin, liver [Grade 1 or 2], or upper GI aGvHD), then the participant will be discontinued from the study and replaced. If a lower GI infection (e.g., CMV, Cdiff) is

diagnosed, then the participant can be enrolled as long as the infection is not uncontrolled and appropriate anti-infective treatment is being initiated and administered.

Prophylactic medication schedules are typically predetermined. The doses of these previously initiated medications are expected to not be changed during the (initial) 7 days of RLS-0071 treatment, but otherwise should be tapered or discontinued as initially planned. If the 7-day dosing period of RLS-0071 and the timing for a change in the prophylactic regimen overlap, then the prophylactic regimen change should be deferred until after the 7-day RLS-0071 dosing period (see Section 7.5.3). Prophylactic medications must be continued as long as initially planned pre-study unless there is a clinical reason (e.g., safety) to taper the prophylactic regimen differently (see Section 7.5.3).

Importantly, participants who initiate medications other than systemic corticosteroids to treat aGvHD prior to initiating RLS-0071 will be excluded. If ruxolitinib had been initiated prior to RLS-0071 to treat aGvHD (e.g., skin aGvHD), that is acceptable as long as RLS-0071 is able to be initiated within 48 hours after ruxolitinib was initiated and there is no evident failure of ruxolitinib treatment.

Participants who require treatment for conditions other than aGvHD should receive those treatments, including appropriate standard-of-care antifungal prophylaxis, antibiotics for fever, antivirals for CMV, pain medications, dysmotility drugs, and TPN, etc.

Duration of RLS-0071 treatment: RLS-0071 treatment will be administered for 7 days in Cohort 1, and for 14 days in Cohorts 2, 3, 4 and 5. For the one or two planned dosing arms of the expansion cohort, the dose of RLS-0071 will be determined based on safety, PK, PD, and efficacy findings in the dose-escalation cohorts.

Use of corticosteroids: Corticosteroids steroids should have been prescribed according to standard of care prior to enrollment with dosing of methylprednisolone (MPE) (or equivalent) until fulfilling criteria for the standard definition of steroid-refractory aGvHD. Steroid-refractory aGvHD as defined in the Inclusion Criteria: progressed after 3 days of treatment with methylprednisolone equivalents (MPE) ≥2 mg/kg/day; did not improve after 7 days of treatment with MPE ≥2 mg/kg/day; progressed to a new organ after treatment with MPE ≥1 mg/kg/day for isolated skin and/or upper GI GvHD; or recurred during or after a steroid taper.

Clinically, there is a balance between enabling a clinical response and avoiding steroid adverse effects when considering the timing of steroid tapering. The corticosteroid tapering parameters are standardized in the protocol (see Section 7.5.2).

Secondary treatment: During the initial 7 days of RLS-0071 +/- ruxolitinib treatment, other secondary treatments are not permitted (corticosteroids expected to continue). After Day 7, alternative treatments are not permitted for participants with a complete response and are discouraged for participants who have attained a very good partial response.

Tertiary treatment: Tertiary treatments may be needed for non-responders or for those participants whose aGvHD progresses. Alternative (i.e. tertiary) treatment is only permitted after at least 3 days of RLS-0071 treatment if there is clear evidence of aGvHD disease progression or discontinuation of RLS-0071 (e.g., for safety). After Day 7, alternative treatment(s) should be initiated for non-responders or for those with disease progression.

Any participant whose GvHD progresses or who receives an alternative therapy would be considered a treatment failure.

Ruxolitinib is standardly tapered after 6 months (180 days) in participants who have clinical response and have discontinued therapeutic doses of corticosteroids. Accordingly, ruxolitinib should not be tapered during the 180 day duration of this clinical study, unless clinically indicated.

Ruxolitinib is associated with thrombocytopenia, anemia, and neutropenia. These adverse reactions have not been observed in the RLS-0071 nonclinical and clinical phase 1 studies and are not expected to occur based on the RLS-0071 mechanism of action. If thrombocytopenia, anemia, or neutropenia occur on study, this should be managed by ruxolitinib dose reduction, or interruption, or blood or platelet transfusion according to the approved prescribing information for ruxolitinib (Incyte Corporation, 2023). For example, for an absolute neutrophil count less than 1 x 10^9/L considered related to ruxolitinib, hold ruxolitinib for up to 14 days and then resume at 1 dose level lower upon recovery.

If participants receiving RLS-0071 are discharged from the hospital, which is unlikely in this time period considering the typical disease severity, they can continue to receive RLS-0071 as outpatients for the duration of the treatment course. Dosing of RLS-0071 will continue to be every 8 hours (Q8H). Participants in Cohort 2a or Cohort 3 who improve and are able to leave the hospital after 7 to 13 days of treatment with RLS-0071 will be given the opportunity to roll over into Cohort 4 or Cohort 5 if they are determined to be eligible after discussion between the PI and Medical Monitor. The outpatient dosing for Cohort 4 and Cohort 5 will be once a day (QD) following discharge to outpatient to completion of Day 14.

Treatment assignment: Participants will be followed for 180 days for safety and efficacy, including durability of initial response.

The study will enroll approximately 21 participants initially, with the potential to expand enrollment to up to 66 participants, and the study design comprises the following cohorts, which are described below and illustrated in Figure 1:

Dose Escalation Cohorts, Enrolled Sequentially based on protocol-specified dose escalation criteria.

7-day dosing:

- **Cohort 1a:** 9 participants will receive 10 mg/kg Q8H RLS-0071 for 7 days with concurrent ruxolitinib. Completion of Cohort 1a will lead to the opening of Cohort 2a, if appropriate.
- **Cohort 1b:** 3 participants will receive 10 mg/kg Q8H RLS-0071 for 7 days without concurrent ruxolitinib. Opens concurrent to Cohort 1a. Completion of Cohort 1b will lead to the opening of Cohort 2b, if appropriate. (Based upon DLT finding the cohort may be expanded to n=6)

14 day dosing in hospitalized participants:

- **Cohort 2a:** 6 participants will receive 10 mg/kg Q8H RLS-0071 for 14 days with concurrent ruxolitinib. Completion of Cohort 2a will either lead to the opening of Cohort 3 or lead to the opening of Expansion Cohort 1, based on safety and clinical response data review.

- **Cohort 2b:** 3 participants will receive 10 mg/kg Q8H RLS-0071 for 14 days without concurrent ruxolitinib. This cohort will not open prior to the opening of Cohort 2a. (based upon DLT finding cohort may be expanded to n=6).
- **Cohort 3:** 6 participants will receive 20 mg/kg Q8H RLS-0071 for 14 days with concurrent ruxolitinib. Completion of Cohort 3 will lead to the opening of Expansion Cohort 2, if appropriate.

If a participant in Cohort 2a or Cohort 3 is rolled into Cohort 4 or 5, then they will be replaced within Cohort 2a or Cohort 3.

14-day dosing with 7 to 13 days inpatient and continued outpatient treatment through Day 14:

Participants in Cohort 2a or Cohort 3 who improve and are able to leave the hospital after 7 to 13 days of treatment with RLS-0071 likely represent a less severe population (e.g. skin disease without intestinal disease). Thus, these participants that are appropriate for hospital discharge after 7 to 13 days are a reasonable population to segregate into outpatient management as would normally be done for participants well enough to go home and are desiring to go home. These less severe participants in Cohort 2a and Cohort 3 will be given the opportunity to roll over into the following cohorts. Determination of eligibility and appropriateness to roll over will be made by the PI in discussion with the participant as well as the Medical Monitor. These participants will be followed as a separate cohort to determine disease characteristics that are likely different from those requiring continued hospitalization.

- **Cohort 4:** up to 3 participants will receive 10 mg/kg Q8H RLS-0071 for 7 to 13 days and then 10 mg/kg QD RLS-0071 to complete 14 days with concurrent ruxolitinib.
- **Cohort 5:** up to 3 participants will receive 20 mg/kg Q8H RLS-0071 for 7 to 13 days and then 20 mg/kg QD RLS-0071 to complete 14 days with concurrent ruxolitinib.

Dose Expansion Cohorts (1 or 2 dosing arms, up to 24 participants total)

- **Expansion Cohort 1:** 12 participants will receive 10 mg/kg Q8H RLS-0071 for 14 days.
- **Expansion Cohort 2:** 12 participants will receive 20 mg/kg Q8H RLS-0071 for 14 days.

Additional participants may be added to any Cohort based on DSMB recommendation.

Figure 1: Treatment Cohorts

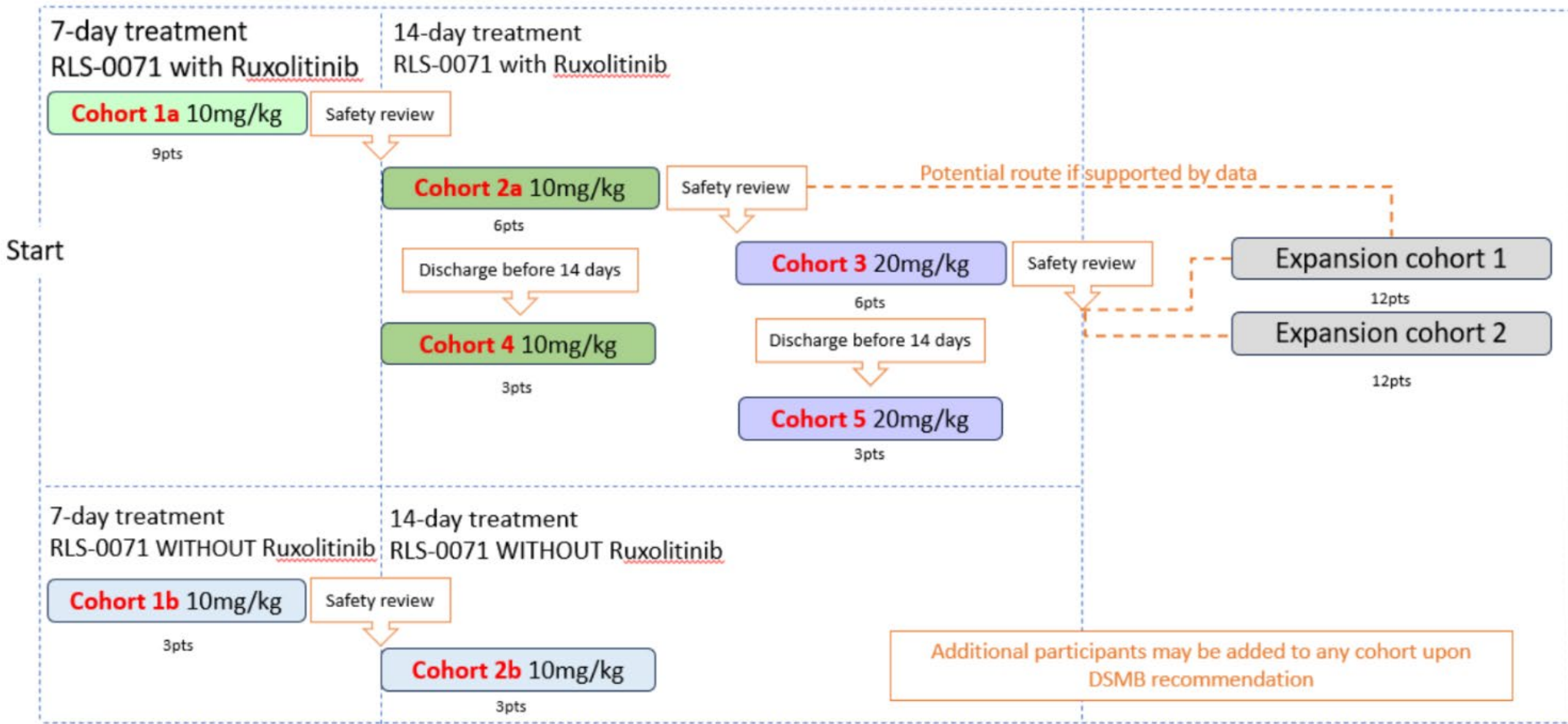

Safety and available PK, PD, and efficacy data will be evaluated for each cohort, prior to advancing to each subsequent dose cohort or into the expansion cohorts. A DSMB will be in place to review these data and make recommendations to the Sponsor. All participants will be followed for at least 14 days in each cohort, prior to dose escalation. Additionally, regimens (dose and duration) shown to be safe in the dose escalation cohorts will be selected for further evaluation in 1 or 2 dose expansion cohorts based on safety, PK, PD, and efficacy. The specific criteria for dose escalation and expansion are defined in Section 8.3.

The study design allows for evaluation of 2 dose levels of RLS-0071 (10 mg/kg and 20 mg/kg), and safety permitting of 7-day vs. 14-day dosing of RLS-0071. This design will be informative for comparative efficacy, safety, pharmacokinetics, pharmacodynamic response, and dose response for RLS-0071.

PK Sampling: Intense PK samples will be collected and evaluated for 3 participants each from dose escalation Cohorts 1 and 3 (6 participants total for intense PK) during the first 7 days of dosing for participants treated with 10 or 20 mg/kg RLS-0071. The remaining study participants will have only sparse PK collected and evaluated.

RLS-0071 should be used with caution upon concomitant administration with sensitive CYP2C19 substrate.

Immunogenicity Assessment: Samples will be collected to evaluate for potential anti-drug antibodies against RLS-0071. Samples will be collected prior to the first dose of RLS-0071 and on Day 28 for all participants. If the Day 28 result is positive, then a repeat sample will be collected on Day 180. The immunogenicity assessment plan and schedule will be described in further detail in Section 9.4.

Additionally, while the study is designed without a concurrent non-RLS-0071 control group, the study efficacy data will also be evaluated in the context of historical control data, including those studies evaluated as part of the ruxolitinib development program for steroid-refractory aGvHD.

5.2 Scientific Rationale for Study Design

5.3 Justification for Dose

RLS-0071 was tested in study RLS-0071-101, a Phase 1 healthy volunteer SAD/MAD design. RLS-0071 demonstrated no safety signals in healthy volunteers at doses of up to 40 mg/kg IV Q8H for 9 doses or single doses up to 120 mg/kg IV. The dosing strategy for aGvHD will utilize a 10 mg/kg IV Q8H for 7 days in Cohort 1, or 10 or 20 mg/kg IV Q8H for 14 days in Cohorts 2 and 3 respectively. This regimen is designed to attain and maintain optimal suppression of inflammation through the typical duration of the most severe gastrointestinal and other aGvHD symptoms.

Consistent with the earlier clinical studies of RLS-0071 as described above, this Phase 2 study in aGvHD initiates enrollment with a 10 mg/kg IV Q8H dose regimen for 7 days. Dosing will be escalated in sequential cohorts as follows: 10 mg/kg IV Q8H dose regimen for 14 days, and 20 mg/kg IV Q8H dose regimen for 14 days. Six (6) participants will be enrolled in each dose level. Safety/tolerability (for at least 14 days) will be confirmed at each dose level, including DSMB review, before escalating to the next dose level. A period of dosing longer than 7 days addresses the time it may take for full clinical response and gut healing. However, the study also allows for the possibility that clinical response can continue to improve beyond Day 7 based on only 7 days of dosing.

Following the dose escalation cohorts, the study is planned to proceed to a dose expansion component with one or two dose arms based on the safety/tolerability, PK, PD, and efficacy findings in the dose escalation cohorts. Each dose expansion cohort is planned to include up to 12 participants (up to 24 participants total for 2 arms in dose expansion).

Overall, the different dose regimens (dose and duration) and the dose escalation and dose expansion components in the study provide the opportunity to establish safety and efficacy in Phase 2 to determine the RLS-0071 dose regimen to advance further in development.

5.4 End of Study Definition

A participant is considered to have completed the study if he/she has completed all phases of the study including the last follow-up evaluation on Day 180.

5.5 Duration of Participation

All participants enrolled in the trial will receive RLS-0071 for 7 days in Cohort 1, and 14 days in Cohorts 2, 3, 4 and 5 during which assessments will be made of safety, PK, efficacy, and PD. Participants will then remain in the study through Day 180 to further assess safety and efficacy. The total duration of participation is 180 days following the screening period.

6. Selection and Withdrawal of Participants

6.1 Participant Inclusion Criteria

To be enrolled in the study, a participant must meet the following criteria:

1. Male or female adults or adolescents (>12 years old).
2. Have undergone first allogeneic hematopoietic stem cell transplantation (allo-HSCT) from any donor source using bone marrow, peripheral blood stem cells, or cord blood for hematologic malignancies. Recipients of nonmyeloablative and myeloablative conditioning regimens are eligible. Any conditioning regimen and any anti-GVHD prophylactic program are permitted.
3. Steroid-refractory aGvHD defined by one or more of the following criteria: progressed after 3 days of treatment with methylprednisolone equivalents (MPE) ≥2 mg/kg/day; did not improve after 7 days of treatment with MPE ≥2 mg/kg/day; progressed to a new organ after treatment with MPE ≥1 mg/kg/day for isolated skin and/or upper GI GvHD; or recurred during or after a steroid taper.
4. Grade II-IV aGvHD as per MAGIC guidelines, defined as:
    a. Grade II: Stage 3 rash and/or Stage 1 liver and/or Stage 1 upper GI and/or Stage 1 lower GI, or
    b. Grade III: Stage 2 liver and/or Stage 2–3 lower GI, with Stage 0–3 skin and/or stage 0-1 upper GI, or
    c. Grade IV: Stage 4 skin, liver, or lower GI involvement, with Stage 0–1 upper GI.

    Note: Patients with Stage 3 or 4 liver are excluded from participation in the study
5. Hospitalized or being admitted to hospital with aGvHD and a Karnofsky Performance Status (KPS) of at least 50 (Table 3). Inclusion of a participant with a KPS lower than 50 requires discussion with the Medical Monitor.
6. Anticipated hospital length-of-stay of at least 1 week from the time of RLS-0071 initiation.
7. Initiating or recently initiated ruxolitinib for secondary treatment of aGvHD; if ruxolitinib is contraindicated or the investigator considers it inadvisable for a specific participant then that participant can be enrolled in Cohort 1b or Cohort 2b and not treated with ruxolitinib.
8. Feasibility to initiate RLS-0071 dosing simultaneously to ruxolitinib or within 48 hours before or after initiation of ruxolitinib (note that from a timing perspective the goal is simultaneous initiation of RLS-0071 and ruxolitinib whenever possible).
9. No plans to add additional GvHD treatment medications or to add, dose-adjust, or discontinue GvHD prophylactic medications during the 7-days of RLS-0071 treatment. Note that participants who require treatments for conditions other than aGvHD should receive those treatments, including standard-of-care antifungal prophylaxis, antibiotics for fever, antivirals for CMV, pain medications, dysmotility agents, and TPN, etc.
10. Neutrophil recovery following the stem-cell transplantation, defined as blood neutrophil count >500/mL for at least 3 consecutive measurements (use of growth factor supplementation, e.g., filgrastim, at that time is permitted).
11. At the time of study screening prior to RLS-0071 initiation:

   a. Neutrophil count >1000/mL (not supported by growth factor supplementation, e.g., filgrastim);
   b. Platelet count >50,000/ml (not supported by platelet transfusions);
   c. Aspartame aminotransferase (AST) and alanine aminotransferase (ALT) < 5 times the upper limit of normal (ULN);
   d. Serum bilirubin < 6mg/dL; and
   e. Adequate coagulation function (e.g., prothrombin time (PT)/international normalized ratio (INR) and activated partial thromboplastin time (aPPT)/partial thromboplastin time (PTT) <2 X ULN.

12. Weight ≥40 kg and ≤ 140 kg at screening.
13. Participant (or legally authorized representative for minors) provides written informed consent; additionally, informed assent for minors.
14. Able to communicate well with the Investigator and/or study site personnel and to comply with the requirements of the entire study.
15. Women participants of childbearing potential (WOCBP) (not surgically sterile) must agree to use highly effective contraception during the study drug treatment period and for 6 additional months following treatment. Follow manufacture recommendation for other medications.
16. Male participants with partners of childbearing potential must agree to use highly effective contraception during the study drug treatment period and for 3 additional months following treatment. Follow manufacture recommendation for other medications.

6.2 Participant Exclusion Criteria

A participant who meets any of the following criteria at Screening will be excluded from participation in this study:

1. Has received more than 1 allo-HSCT.
2. Current, previous, or planned (in the initial 7 days) use of any systemic treatment in addition to or other than corticosteroids or ruxolitinib (unless initiated within 48 hours of enrollment per Section 6.1) for aGvHD (previously initiated aGvHD prophylaxis is permitted to continue).
3. Previous failure of ruxolitinib treatment.
4. Uncontrolled GI infection (e.g., CMV, Cdiff); note that participants with controlled GI infections can be enrolled as long as appropriate anti-infective treatment is initiated and administered.
5. Endoscopic and biopsy testing (if performed) that definitively rules out lower GI aGvHD in those who are clinically suspected of having lower GI aGvHD; those participants should not be enrolled or should be discontinued from the study (and will be replaced) unless they otherwise meet the study entry criteria for skin, liver, and/or upper GI aGvHD.
6. Chronic GvHD (including presence of GVHD overlap syndrome as per NIH guidelines).
7. Participants with evidence of relapsed primary disease, or participants who have been treated for relapse after the allo-HSCT was performed.

8. Unresolved toxicity or complications (other than aGvHD) due to the allo-HSCT, which in the opinion of the Investigator would pose a safety risk for participation in this trial.
9. Any corticosteroid therapy for indications other than aGvHD at doses of methylprednisolone or equivalent >1 mg/kg per day within 7 days of enrollment.
10. Severe organ dysfunction unrelated to underlying aGvHD, including:
    a. Cholestatic disorders or unresolved veno-occlusive disease of the liver (defined as persistent bilirubin abnormalities not attributable to GvHD and ongoing organ dysfunction).
    b. Clinically significant or uncontrolled cardiac disease including unstable angina, acute myocardial infarction within 6 months from Day 1 of study drug administration, New York Heart Association Class III or IV congestive heart failure, circulatory collapse requiring vasopressor or inotropic support, or arrhythmia that requires therapy.
    c. Clinically significant respiratory disease that requires mechanical ventilation support or oxygen supplementation.
11. Known hypersensitivity, allergy, or anaphylactic reaction to polyethylene glycol (PEG) or PEG containing material.
12. Severe kidney disease, with an eGFR < 30 mL/min/1.73m^2 (CKD Stages 4-5).
13. Significant liver disease not related to GvHD
14. Participation in any clinical research study evaluating an investigational product (IP) or therapy for GvHD prophylaxis or treatment within 1 month and less than 5 half-lives of IP prior to the Screening visit.
15. Currently breast feeding.
16. Any medical or psychiatric condition deemed clinically significant by the Investigator or Sponsor such that:
    a. Participation of the study would be unsafe,
    b. OR the condition would make study results uninterpretable.
17. Unable or unwilling to cooperate with the site staff for any reason.
18. Known pregnancy, a positive pregnancy test at screening, or lactation for WOCBP.
19. Active hepatitis B virus (HBV) or hepatitis C virus infection that requires treatment or human immunodeficiency virus (HIV)-1 or HIV-2.
20. Active sepsis

6.3 Screen Failures

Screen failures are defined as participants who consent to participate in the clinical study but are not subsequently enrolled to receive study intervention because they fail to satisfy one or more of the inclusion/exclusion criteria. Participants who fail screening will not be permitted to rescreen.

6.4 Replacement of Participants

Participants who are enrolled but do not receive study intervention will be replaced. Participants will be discontinued from the study and will be replaced if endoscopic and biopsy testing is performed that clearly rules out lower GI aGvHD unless they otherwise meet the study entry criteria for skin, liver, and/or upper GI aGvHD.

If a participant is discharged from the hospital and moved to QD dosing between 7 to 13 days, after discussion between the PI and Medical Monitor, they will be moved to Cohort 4 or Cohort 5 and replaced in Cohort 2a or Cohort 3.

Table 3: Karnofsky Performance Status Scale Definitions Rating (%) Criteria

Able to carry on normal activity and to work; no special care needed.	100	Normal no complaints; no evidence of disease.
	90	Able to carry on normal activity; minor signs of symptoms or disease.
	80	Normal activity with effort; some signs or symptoms of disease.
Unable to work; able to live at home and care for most personal needs; varying amount of assistance needed.	70	Cares for self; unable to carry on normal activity or to do active work.
	60	Requires occasional assistance, but is able to care for most of their personal needs.
	50	Requires considerable assistance and frequent medical care.
Unable to care for self; requires equivalent of institutional or hospital care; disease may be progressing rapidly.	40	Disable; requires special care and assistance.
	30	Severely disabled; hospital admission is indicated although death is not imminent.
	20	Very sick; hospital admission necessary; active supportive treatment necessary.
	10	Moribund; fatal processes progressing rapidly.
	0	Dead

(Crooks et al., 1991; de Haan et al., 1993; Hollen et al., 1994; O'Toole and Golden, 1991; *Oxford Textbook of Palliative Medicine*, 1993; Schag et al., 1984)

7. Treatment of Participants

Study treatment is defined as any investigational intervention(s), marketed product(s), or medical device(s) intended to be administered to a study participant according to the study protocol.

This Phase 2 study will enroll approximately 21 participants initially, with the potential to expand enrollment to up to 66 participants, and the study design comprises:

- Dose Escalation:
    - Three sequential cohorts of 6 or more participants each at different dose levels and durations of RLS-0071 in combination with ruxolitinib (approximately 21 participants)
    - Two sequential cohorts at different dose levels and durations of RLS-0071 not in combination with ruxolitinib (up to 12 total)
    - Two (2) roll-over cohorts with outpatient management of participants in the 14-day group that are discharged from the hospital treated with RLS-0071 in combination with ruxolitinib (up to 6 total)
- Dose Expansion:
    - One or two cohorts of up to 12 participants each at dose levels and durations informed by the results of the dose escalation (up to 24 total).
- Each participant completing the study will be evaluated for up to 180 days from Baseline through Follow Up.

Additional participants may be added to any Cohort based on DSMB recommendation.

7.1 Treatment to be Administered

The investigational product, RLS-0071, is a peptide that is provided as a solution, in vials, at a concentration of 80 mg/mL. RLS-0071 is prepared in a histidine buffered solution (0.05 M histidine, pH 6.5). The solution is clear and faintly colored.

In both parts of the study, RLS-0071 will be administered by IV infusion over a period of 7.5 minutes (± 1 minutes) by study site personnel.

RLS-0071 will be administered as an intravenous (IV) infusion in all study participants. Dosing will be 10 mg/kg or 20 mg/kg for either 7 or 14 days. Study intervention will be given in addition to aGvHD systemic corticosteroid standard of care management for at least 7 days and +/- ruxolitinib as an approved secondary treatment for steroid refractory aGvHD. The frequency of RLS-0071 dosing will be 3-times daily every 8 hours (Q8H). Participants in Cohort 2a or Cohort 3 who improve and are able to leave the hospital after 7 to 13 days of treatment with RLS-0071 will be given the opportunity to roll over into Cohort 4 or Cohort 5 following discussion between the PI and Medical Monitor. Determination of eligibility to roll over will be made by the PI in discussion with the Medical Monitor. The outpatient dosing for Cohort 4 and Cohort 5 will be QD following discharge to outpatient to completion of Day 14 (approximate range of 28 - 42 total doses of RLS-0071).

Study interventions are summarized in Table 4 (7 day dosing cohorts) and Table 5 (14 day dosing cohorts) as well as illustrated in Figure 1. Doses (mg/kg) will be prepared based on the participant's weight at Screening.

Table 4: Dose Escalation Study Interventions (7-day dosing Cohorts)

**Arm Name**	**Cohort 1a**	**Cohort 1b**
**Intervention Name**	RLS-0071	RLS-0071
**Type**	Drug	Drug
**Dose Formulation**	Vial	Vial
**Unit Dose Strength**	80 mg/mL	80 mg/mL
**Dosage Level**	10 mg/kg	10 mg/kg
**Route of Administration**	IV infusion	IV infusion
**Ruxolitinib Use**	Yes	No
**Dosing Frequency**	Q8H	Q8H
**Dosing Length**	7 days (21 total doses)	7 days (21 total doses)
**Use**	Experimental	Experimental
**Sourcing**	Provided centrally by the Sponsor	Provided centrally by the Sponsor
**Storage**	-20°C ± 5°C	-20°C ± 5°C
**Packaging and Labeling**	Study intervention will be provided in vials. Each vial will be labeled as required per country requirement	Study intervention will be provided in vials. Each vial will be labeled as required per country requirement

Abbreviations: IV=intravenous; Q8H=every 8 hours

Table 5: Dose Escalation Study Interventions (14-day dosing Cohorts)

Arm Name	Cohort 2a	Cohort 2b	Cohort 3	Cohort 4	Cohort 5
**Intervention Name**	RLS-0071	RLS-0071	RLS-0071	RLS-0071	RLS-0071
**Type**	Drug	Drug	Drug	Drug	Drug
**Dose Formulation**	Vial	Vial	Vial	Vial	Vial
**Unit Dose Strength**	80 mg/mL	80 mg/mL	80 mg/mL	80 mg/mL	80 mg/mL
**Dosage Level**	10 mg/kg	10 mg/kg	20 mg/kg	10 mg/kg	20 mg/kg
**Route of Administration**	IV infusion	IV infusion	IV infusion	IV infusion	IV infusion
**Ruxolitinib Use**	Yes	No	Yes	Yes	Yes
**Dosing Frequency**	Q8H	Q8H	Q8H	Q8H then QD post discharge	Q8H then QD post discharge
**Dosing Length**	14 days (42 total doses)	14 days (42 total doses)	14 days (42 total doses)	14 days (approx. range of 28 - 42 total doses)	14 days (approx.. range of 28 - 42 total doses)
**Use**	Experimental	Experimental	Experimental	Experimental	Experimental
**Sourcing**	Provided centrally by the Sponsor	Provided centrally by the Sponsor	Provided centrally by the Sponsor	Provided centrally by the Sponsor	Provided centrally by the Sponsor
**Storage**	-20°C ± 5°C	-20°C ± 5°C	-20°C ± 5°C	-20°C ± 5°C	-20°C ± 5°C
**Packaging and Labeling**	Study intervention will be provided in vials. Each vial will be labeled as required per country requirement	Study intervention will be provided in vials. Each vial will be labeled as required per country requirement	Study intervention will be provided in vials. Each vial will be labeled as required per country requirement	Study intervention will be provided in vials. Each vial will be labeled as required per country requirement	Study intervention will be provided in vials. Each vial will be labeled as required per country requirement

Abbreviations: IV=intravenous; Q8H=every 8 hours; QD=once daily

7.2 Preparation/Handling/Storage/Accountability

The study interventions supplied by the Sponsor are to be used exclusively in the clinical study according to the instructions of this protocol. The Investigator is responsible for dispensing the study interventions according to the dosage scheme and for ensuring proper storage of the study interventions.

1. The Investigator or qualified designee must confirm appropriate temperature conditions have been maintained during transit for all study intervention received and any discrepancies are to be reported and resolved before use of the study intervention. Only participants enrolled in the study may receive study intervention and only authorized site staff may supply or administer study intervention. All study intervention must be stored in a secure, environmentally controlled, and monitored (manual or automated) area in accordance with the labeled storage conditions with access limited to the Investigator and authorized site staff.
2. The Investigator, institution, or the head of the medical institution (where applicable) is responsible for study intervention accountability, reconciliation, and record maintenance (i.e., receipt, reconciliation, and final disposition records).
3. Further guidance and information for the final disposition of unused study interventions are provided in the Pharmacy Manual.

Details on the preparation of RLS-0071 for infusion are provided in the Pharmacy Manual.

7.3 Procedures for Monitoring Participant Compliance

Participants are dosed at the hospital or outpatient clinic and will receive study intervention directly from the Investigator or qualified designee, under medical supervision. The date and time of each dose administered in the clinic will be recorded in the source documents and recorded in the electronic case report form (eCRF). The dose of study intervention and study participant identification will be confirmed at the time of dosing by a member of the study site staff other than the person administering the study intervention.

7.4 Medication(s) Treatment(s) Permitted and Not Permitted

Any medication (including over the counter or prescription medicines, vitamins, and/or herbal supplements) that the participant has received within 30 days before Day 1 or receives during the study (through the Day 180 visit) must be recorded along with:

- Reason for use
- Dates of administration including start and end dates
- Dosage information including dose and frequency.

The Sponsor's Medical Monitor should be contacted if there are any questions regarding concomitant or prior therapy.

7.4.1 Corticosteroids

The systemic corticosteroid dose should be ≥2 mg/kg methylprednisolone or equivalent for initial treatment of aGvHD prior to enrollment, parameters for tapering are described in Section 7.5.2.

7.4.2 Ruxolitinib

RLS-0071 should be administered simultaneously to the time of ruxolitinib, but the time interval between ruxolitinib and RLS-0071 initiations must be less than 48 hours of each other. Note that as part of the study design, when ruxolitinib is planned from the time of study initiation and is administered after RLS-0071 initiation but within 48 hours, then the "addition" of ruxolitinib will not be considered as an RLS-0071 treatment failure.

If ruxolitinib is contraindicated or the investigator considers it inadvisable for a specific participant, then that participant can be enrolled and not treated with ruxolitinib. They will be enrolled in Cohort 1b or Cohort 2b.

Dosing and dose modifications for ruxolitinib should be done according the Jakafi Package Insert (Incyte Corporation, 2023).

7.4.3 Treatments for aGvHD

No medications other than systemic corticosteroids to treat aGvHD are allowed prior to initiating RLS-0071. If ruxolitinib had been initiated prior to RLS-0071 to treat aGvHD (e.g., skin aGvHD), that is acceptable as long as RLS-0071 is able to be initiated within 48 hours after ruxolitinib was initiated and there is no evident failure of ruxolitinib treatment.

During the initial 7 days of RLS-0071 +/- ruxolitinib treatment, other secondary treatments are not permitted (corticosteroids expected to continue). After Day 7, alternative treatments are not permitted for participants with a complete response and are discouraged for participants who have attained a very good partial response.

Tertiary treatments may be needed for non-responders or for those participants whose aGvHD progresses. Alternative (i.e. tertiary) treatment is only permitted after at least 3 days of RLS-0071 treatment if there is clear evidence of aGvHD disease progression or if RLS-0071 needed to be discontinued (e.g., for safety). After Day 7, alternative treatment(s) should be initiated for non-responders or for those with disease progression.

Any participant whose GvHD progresses or who receives an alternative therapy would be considered a treatment failure.

7.4.4 Treatments for Conditions other than aGvHD

Participants who require treatment for conditions other than aGvHD should receive those treatments, including standard-of-care antifungal prophylaxis, antibiotics for fever, antivirals for CMV, pain medications, dysmotility agents, total parenteral nutrition (TPN), etc.

Prophylactic medication schedules are typically predetermined. The doses of these previously initiated medications are expected to not be changed during the (initial) 7 days of RLS-0071 treatment, but otherwise should be tapered or discontinued as initially planned. If the 7-day dosing period of RLS-0071 and the timing for a change in the prophylactic regimen overlap, then the prophylactic regimen change should be deferred until after the 7-day RLS-0071 dosing period (see Section 7.5.3). Prophylactic medications must be continued as long as initially planned pre-study unless there is a clinical reason (e.g., safety) to taper the prophylactic regimen differently (see Section 7.5.3).

Investigational treatments for aGvHD (other than RLS-0071 study drug) are prohibited. Systemic corticosteroids and ruxolitinib are not considered investigational treatments for aGvHD. Other than corticosteroids, no prior aGvHD treatment is permitted.

7.4.5 Contraception

WOCBP must agree to use at least one primary form of highly effective contraception during the study treatment and for an additional 6 months following treatment. Women must agree to not donate eggs for at least 6 months after the last dose of study intervention. Follow manufacture recommendation for other medications.

Acceptable forms of highly effective contraception are:

a. Abstinence from heterosexual intercourse
b. Hormonal contraceptives (birth control pills, injectable/implant/insertable hormonal birth control products, transdermal patch) and a barrier method (e.g., condom) from the Screening visit and until the participant completes the study.
c. Intrauterine device (with or without hormones)

To be considered of nonchildbearing potential, female participants must meet the following requirements:

a. Surgically sterile (documented hysterectomy, tubal ligation, or bilateral salpingo-oophorectomy at least 3 months prior to Day 1) or
b. Postmenopausal (defined as 12 months from the time of last spontaneous amenorrhea). A follicle-stimulating hormone (FSH) level > 35 IU/L measured prior to or at Screening will be considered confirmatory in the absence of a clear postmenopausal history.

Men must agree to be abstinent from heterosexual activity or use a barrier contraception method (e.g., condom) during the study treatment and for an additional 3 months following treatment. Follow manufacture recommendation for other medications. Men must agree to not donate sperm for at least 3 months after the last dose of study intervention.

7.4.6 Medications to be used with Caution

7.4.6.1 CYP2C19 Substrates

Caution should be used with concomitant administration of RLS-0071 with sensitive CYP2C19 substrates.

7.4.6.2 CYP3A4 Inhibitors or Fluconazole

Avoid concomitant use of Ruxolitinib with fluconazole doses of greater than 200 mg daily. Dose modification should be done according to Table 9 and the Jakafi Package Insert (Incyte Corporation, 2023).

7.4.7 Prohibited Medications

Investigational treatments for aGvHD (other than RLS-0071 study drug) are prohibited. Systemic corticosteroids and ruxolitinib are not considered investigational treatments for aGvHD. Other than corticosteroids, no prior aGvHD treatment is permitted.

Participants who have received any investigational agent (i.e., no FDA approved label) within 5 half-lives prior to the Screening visit, will be excluded from the study. Off-label or investigational use of FDA approved medications for indications other than GvHD is permitted.

7.5 Timing and Order of Medication Taper

7.5.1 RLS-0071

RLS-0071 will be administered for 7 or 14 days, based on dose assignment. The medication will then be stopped after the assigned time period is completed.

7.5.2 Corticosteroids

Participants enrolled in this study will be steroid refractory, and there is a range of steroid tapering approaches used clinically in these participant types. For some participants, the steroid may be providing some benefit even though the participant is refractory, supporting continuation of the steroids for several additional days before initiating tapering. For other participants, if the steroid is likely providing no benefits and/or there are adverse event considerations, the steroid taper is begun immediately. The protocol allows for these different scenarios by providing 2 tapering schedules that align with these scenarios, providing standardization in the protocol. From a clinical study perspective, it is expected that the conservative tapering schedule is followed to prevent medication changes during the initial 7 days of RLS-0071 treatment. The more aggressive tapering schedule is permitted if the Investigator has determined that the corticosteroid is providing no benefit, or the participant has an evident safety issue with the corticosteroids.

After the pre-study standard of care corticosteroid regimen (which will most likely be 2 mg/kg methylprednisolone (or equivalent) for 3-7 days) until fulfilling criteria for the standard definition of steroid-refractory aGvHD and subsequent enrollment, the 2 steroid tapering regimens based on methylprednisolone (or equivalent) are (Figure 2):

- Conservative taper: Upon enrollment continue 2 mg/kg for the first 7 days on study protocol, then 1.5 mg/kg for 7 days, then 1 mg/kg for 7 days (this is the expected regimen)
- Aggressive taper: Upon enrollment decrease to 1.5 mg/kg for 4 days, then 1 mg/kg for 4 days (permitted if corticosteroid is providing no benefit or the participant has an evident safety issue with the corticosteroid)

If an Investigator selects the aggressive taper for a specific participant, the clinical rationale (i.e., lack of benefit or safety issue) should be recorded in the study eCRF. After the steroid dose has been tapered to 1.0 mg/kg, the Investigator has the discretion to maintain participants at that dose, or to further taper or discontinue the steroids.

Figure 2: Corticosteroid Taper

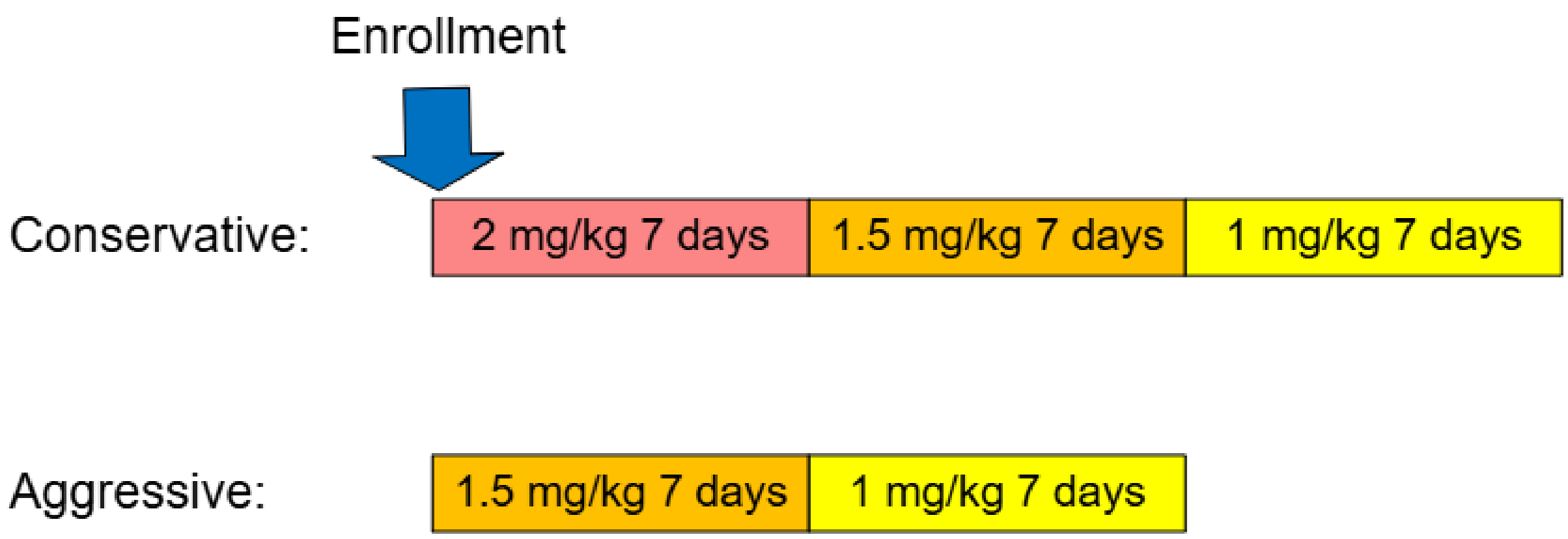

Note that if the taper had initiated prior to enrollment (e.g., 1-2 days prior) and the participant otherwise qualifies for the study, then the Investigator should still follow one of the 2 tapering schedules as closely as possible, based on the parameters described above.

Note too that if a participant's dose is not precisely 2 mg/kg methylprednisolone (or equivalent), then the Investigator should still adhere to one of the 2 tapering options but initiate the taper from the dose that participant is on and decrease from there by 0.5 mg/kg decrements.

The goal is to discontinue corticosteroids or achieve a minimal dose, although ideally the corticosteroid dose is not changed during the (first) 7 days of RLS-0071 treatment.

Note that if aGvHD had flared previously during or following a steroid taper or flares during steroid tapering in this study and the aGvHD is brought again under control, these same 2 options for schedule of tapering should be followed to provide an additional opportunity for steroid tapering. In this scenario, if the Investigator assesses that there is a concern (e.g., safety) for a specific participant with the protocol tapering schedule, then the Investigator can follow Institutional guidance in consultation with the study Medical Monitor, and these changes must be documented in the study records.

7.5.3 Prophylactic Medication

Prophylactic medication schedules are typically predetermined. The doses of these previously initiated medications are expected not be changed during the (initial) 7 days of RLS-0071 treatment, but otherwise should be tapered or discontinued as initially planned. If the 7-day dosing period of RLS-0071 and the timing for a change in the prophylactic regimen overlap, then the prophylactic regimen change should be deferred until after the 7-day RLS-0071 dosing period. Prophylactic medications must be continued as long as initially planned pre-study unless there is a clinical reason (e.g., safety) to taper the prophylactic regimen differently.

7.5.4 Ruxolitinib

Ruxolitinib is typically continued for 6 months, and as this study is 6 months (180 days) in duration ruxolitinib should not be tapered during this study, unless clinically indicated.

7.6 Dose Modification

Study intervention will be administered as an IV infusion over 7.5 minutes (± 1 minutes). Participants in Cohort 1a/Cohort 1b will receive study intervention Q8H (± 1 hour) for 7 days (up to 21 consecutive doses total). Participants in Cohort 2a/Cohort 2b and Cohort 3 will receive study intervention Q8H (± 1 hour) for 14 days (up to 42 consecutive doses total). Participants in Cohort 2a or Cohort 3 who improve and are able to leave the hospital after 7 to 13 days of treatment with RLS-0071 will be given the opportunity to roll over into Cohort 4 or Cohort 5 following discussion between the PI and the participant as well as the Medical Monitor. Determination of eligibility to roll over will be made by the PI in discussion with the Medical Monitor. The outpatient dosing for Cohort 4 and Cohort 5 will be QD following discharge to outpatient to completion of Day 14 (approximate range of 28 - 42 total doses of RLS-0071).

Ruxolitinib is associated with thrombocytopenia, anemia, and neutropenia. These adverse reactions have not been observed in the RLS-0071 nonclinical and clinical phase 1 studies and are not expected to occur based on the RLS-0071 mechanism of action. If thrombocytopenia, anemia, or neutropenia occur on study, this should be managed by ruxolitinib dose reduction, or interruption, or transfusion according to the approved prescribing information for ruxolitinib. For example, for an absolute neutrophil count less than 1×10^9/L considered related to ruxolitinib, hold ruxolitinib for up to 14 days and then resume at 1 dose level lower upon recovery.

Additional dose modification of RLS-0071 is not permitted except as stated in the following sections. Refer to Section 8 for information regarding discontinuation of study intervention.

7.6.1 Dose Modification in Case of Infusion-Related Reaction

Example of Infusion-related Reactions include:

- Fever
- Nausea
- Chills
- Bronchospasm

The grade of the observed infusion-related reactions are defined as follows:

- Grade 1: Mild transient reaction; infusion interruption not indicated; intervention not indicated.
- Grade 2: Therapy or infusion interruption indicated but responds promptly to symptomatic treatment (e.g., antihistamines, NSAIDS, narcotics, IV fluids); prophylactic medications indicated for ≤24 hrs.
- Grade 3: Prolonged (e.g., not rapidly responsive to symptomatic medication and/or brief interruption of infusion); recurrence of symptoms following initial improvement; hospitalization indicated for clinical sequelae.
- Grade 4: Life-threatening consequences; urgent intervention indicated.

Management of Infusion Related Reactions should be done according to Table 6. If a participant has an infusion-related reaction following a re-challenge they should be managed according to Table 7. If the study intervention infusion is stopped prematurely and not restarted, the participant should remain on study through Day 180, if possible, to be followed for safety (see Section 8).

The Investigator will inform the Sponsor's Medical Monitor as soon as possible if any adverse event (AE) results in temporary delay of treatment, interruption of an IV infusion, or discontinuation of study intervention administration dosing.

Table 6: Management of Infusion Related Reactions

Infusion Related Reactions	Management for Current Dose of RLS-0071	Subsequent Doses of RLS-0071
≤ Grade 1	No change in dose	Premedicate with diphenhydramine and acetaminophen. No change in dose. Flow rate decreased by 50%.
Grade 2	Pause infusion for 15 minutes and resume at lower flow rate decreased by 50%	Premedicate with diphenhydramine and acetaminophen. No change in dose. Flow rate decreased by 50%.
Grade 3	Pause infusion for 30 minutes or until ≤ Grade 1, whichever is longer. Resume at lower flow rate decreased by 50%	Premedicate with diphenhydramine and acetaminophen. No change in dose. Flow rate decreased by 50%.
Grade 4	Stop treatment and remove from study.	Off protocol therapy

Table 7: Management of Subsequent Infusion Related Reactions after Re-challenge

Infusion Related Reactions	Management for Current Dose of RLS-0071
≤ Grade 1	Premedicate with diphenhydramine and acetaminophen. Pause infusion for 15 minutes are resume at lower flow rate (50%)
Grade 2	Pause infusion for 30 minutes or until ≤ Grade 1, whichever is longer. Resume at lower flow rate. (50%)
Grade 3	Off protocol therapy
Grade 4	Off protocol therapy

If CTCAE Grade 1-2 infusion-related reactions are observed in 2 or more participants in a given dose cohort, the DSMB may choose to recommend modification of the infusion or addition of premedication with diphenhydramine and acetaminophen for all participants based on medical judgment as part of its dose escalation recommendation following a review of the accumulating safety data.

7.6.2 Dose Modification in Case of Moderate Renal Impairment

Dose modification of ruxolitinib should be done according to Table 8 and the Jakafi Package Insert (Incyte Corporation, 2023).

Table 8: Dose Modifications for Ruxolitinib for Concomitant Use with Renal Impairment

Renal Impairment Status	Recommended Ruxolitinib Starting Dose
Moderate Renal Impairment (eGFR ≥30 mL/min/1.73m^2 but <60 mL/min/1.73m^2)	5 mg once daily for participants with aGVHD

7.6.3 Dose Modification in Case of Concomitant Use with Strong CYP3A4 Inhibitors or Fluconazole

Dose modification of ruxolitinib should be done according to Table 9 and the Jakafi Package Insert (Incyte Corporation, 2023).

Table 9: Dose Modifications for Ruxolitinib for Concomitant Use with Strong CYP3A4 Inhibitors or Fluconazole

Starting dose for participants with aGVHD	Recommended Ruxolitinib Dose Modification
Fluconazole doses of less than or equal to 200 mg	5 mg once daily for participants with aGVHD
Other CYP3A4 inhibitors	Monitor blood counts more frequently for toxicity and modify the Jakafi dosage for adverse reactions if they occur

7.7 Intervention After the End of the Study

Participants will receive standard of care treatment for management of their aGvHD after the end of the study, as needed.

8. Discontinuation of Study Intervention and Participant Discontinuation/Withdrawal

8.1 Discontinuation of Study Intervention

The Investigator may consider it necessary for a participant to permanently discontinue (definitive discontinuation) study intervention, in the interests of his/her safety. If study intervention is definitively discontinued, the participant will remain in the study to be evaluated for safety.

8.2 Dose Limiting Toxicities and Study Stopping Rules

Dose Limiting Toxicities (DLTs) will be the determining factor for continued dosing of individual participants, administration of higher doses of RLS-0071 to new participants, and whether additional participants may be enrolled in subsequent phases of the study.

For the purposes of this study, A DLT is defined by the following criteria:

1. A > Grade 3 treatment emergent adverse event (TEAE) that occurs at any time from the initial dose of RLS-0071 through to the last RLS-0071 dose administered +7 days. If the TEAE is clearly and incontrovertibly attributable to another medication or the underlying disease (see Sections 8.2.1, 10.9.2, and 10.9.3), then this will not be a DLT. For more information and a complete list of adverse events, see the Jakafi (ruxolitinib) prescribing information (Incyte Corporation, 2023). The TEAEs that will be of particular focus for the Sponsor, Medical Monitor and DSMB are infections and engraftment.
2. A > Grade 3 TEAE that involves an allergic reaction/anaphylaxis.
3. Inability to receive dosing on at least 50% of the planned study dosing days due to a treatment-related adverse event. If the adverse event preventing dosing of RLS-0071 is clearly and incontrovertibly attributable to another medication or the underlying disease (see Sections 8.2.1, 10.9.2, and 10.9.3), then inability to dose will not be a DLT.

Adverse event grading will be defined by the CTCAEv5.0.

8.2.1 AEs that are Generally Considered Related to Another Medication or the Underlying Disease

8.2.1.1 Expected Adverse Events due to Ruxolitinib

In acute graft-versus-host disease, the most common hematologic adverse reactions with ruxolitinib (incidence > 50%) are anemia, thrombocytopenia, and neutropenia.

The most common nonhematologic adverse reactions with ruxolitinib (incidence > 50%) are infections (pathogen not specified) and edema.

For more information and a complete list of adverse events, see the Jakafi (ruxolitinib) prescribing information (Incyte Corporation, 2023).

8.2.1.2 Expected aGvHD Manifestations

- Skin: rash (especially an extensive rash); generalized erythroderma plus bullous formation and desquamation
- Liver: elevated bilirubin
- Upper GI: nausea, vomiting, or anorexia

- Lower GI: diarrhea (especially of high or increased volume or frequency; severe abdominal pain (without ileus); grossly bloody stool

8.2.2 Dose Escalation and Study Stopping Rules

The study will pause to assess safety and DLTs after the enrollment of the first 3 participants of each cohort with a new dosing regimen. The DLT review will be conducted with the DSMB. The DSMB will review relevant safety data and provide a recommendation on whether to continue, modify, temporarily suspend, or terminate the study.

Additionally, enrollment may be paused if the Sponsor judges it is necessary for medical, safety, regulatory, or other reasons consistent with applicable laws, regulations, and Good Clinical Practice (GCP).

8.3 Decision to Proceed with Enrollment in the Next Cohort or Dose Expansion

Enrollment in each Dose Escalation Cohort will be sequential. All participants will be followed for at least 14 days in each cohort, prior to starting enrollment in the next cohort. However, if any of the stopping rules in Section 8.2 are met, the DSMB will be convened to recommend if the study should proceed to the next cohort. Additional participants may be added to any cohort based on DSMB recommendation.

8.3.1 Dose Expansion

At the end of the dose escalation phase, the Sponsor and DSMB will review all available safety, pharmacokinetic and pharmacodynamic data prior to opening the dose expansion phase. The optimal dose and dosing regimen to be used in the expansion cohort(s) will be determined based on the totality of safety, tolerability, clinical activity, and pharmacokinetic (PK) data as appropriate. The tolerability of doses administered in cohorts after the dose limiting toxicity (DLT) observation window should be taken into account in determination of the dose and/or dosing regimen. For example, if 2 dose levels have equivalent PKs and the lower dose has a better safety profile, this dose may be chosen as the optimal regimen. The optimal regimen may be determined to be the highest dose level, the maximum tolerated dose (MTD), or it may be a lower dose based on the determination of the Sponsor and recommendation of the DSMB. Once the optimal dose and dosing regimen(s) are determined, the selected regimen(s) will be operationally implemented in the designated expansion cohort(s). An additional 12 participants will be dosed in one or two expansion cohorts. For the expansion cohort, participants will continue to be monitored for occurrence of DLT. If 2 of the first 6 participants experience DLT, the Sponsor and DSMB will assess if enrollment should continue to the next 6 participants in the expansion cohort.

8.4 Participant Discontinuation/Withdrawal from the Study

Discontinuation from the study will be as follows:

- A participant may withdraw from the study at any time at his/her own request or may be withdrawn at any time at the discretion of the Investigator for safety, behavioral, compliance, or administrative reasons. This is expected to be uncommon.
- At the time of discontinuing from the study, if possible, an early termination (ET) visit should be conducted, as shown in the SoA (see Section 1.2).

- The participant will be permanently discontinued both from the study intervention and from the study at that time.
- If the participant withdraws consent for disclosure of future information, the Sponsor may retain and continue to use any data collected before such a withdrawal of consent.

8.5 Lost to Follow-Up

Participants in the study will have Follow-Up visits on Day 14 (± 2 days) (7-day dosing cohorts only), Day 28 (± 4 days), Day 56 (± 7 days), and Day 180 (± 14 days). Evaluations to be conducted at these visits are shown in Table 1 (7-day dosing Cohorts) and Table 2 (14-day dosing Cohorts).

A participant will be considered lost to follow-up if he or she repeatedly fails to return for scheduled visits and is unable to be contacted by the study site.

The following actions must be taken if a participant fails to return for a required study visit:

- The site must attempt to contact the participant and reschedule the missed visit as soon as possible. Site staff should counsel the participant on the importance of maintaining the assigned visit schedule and ascertain whether or not the participant wishes to and/or should continue in the study.
- Before a participant is deemed lost to follow-up, the Investigator must make a reasonable effort to regain contact with the participant (where possible, 3 telephone calls and, if necessary, a certified letter to the participant's last known mailing address or local equivalent methods). These contact attempts should be documented in the participant's medical record.
- Should the participant not be reachable following completion of the aforementioned contact attempts, he/she will be considered to have withdrawn from the study.

8.6 Study Discontinuation

The Sponsor designee reserves the right to close the study site or terminate the study at any time for any reason at the sole discretion of the Sponsor. Study sites will be closed upon study completion. A study site is considered closed when all required documents and study supplies have been collected and a study site closure visit has been performed.

The Investigator may initiate study site closure at any time, provided there is reasonable cause and sufficient notice is given in advance of the intended termination. Reasons for the early closure of a study site by the Sponsor or Investigator may include but are not limited to:

- Failure of the Investigator to comply with the protocol, the requirements of the Institutional Review Board (IRB)/Independent Ethics Committee (IEC) or local health authorities, the Sponsor's procedures, or GCP guidelines.
- Inadequate recruitment of participants by the Investigator.
- Discontinuation of further study intervention development.

If the study is prematurely terminated or suspended, the Sponsor shall promptly inform the Investigators, the IRB/IECs, the regulatory authorities, and any Contract Research Organizations (CROs) used in the study of the reason for termination or suspension, as specified by the

applicable regulatory requirements. The Investigator shall promptly inform the participant and should ensure appropriate participant therapy and/or follow-up.

9. Study Assessments and Procedures

Study procedures and their timing are summarized in the SoA (Table 1 [7-day dosing Cohorts] and Table 2 [14-day dosing Cohorts]). Adherence to the study design requirements, including those specified in the applicable SoA, is essential and required for study conduct.

9.1 Study Procedures

9.1.1 Screening/Baseline Procedures (Day 0)

All screening evaluations must be completed and reviewed to confirm that potential participants meet all eligibility criteria. The Investigator will maintain a screening log to record details of all participants who provide informed consent, and to confirm eligibility or record reasons for screen failure, as applicable. The participant must provide written informed consent before any assessments are made under this protocol. However, assessments performed as part of standard of care within 24 hours before obtaining informed consent may be used for Screening values.

Eligible participants will be hospitalized and have steroid refractory aGvHD after allo-HSCT with any conditioning regimen will be enrolled and will meet all of the inclusion criteria and none of the exclusion criteria. Every effort will be made to complete Screening procedures as quickly as possible.

Participants will be identified at study sites and will undergo the informed consent process. After providing written informed consent, participants will undergo a review of their demographic (age, race, ethnicity), medical history (including organ impairment), an assessment of the inclusion/exclusion criteria for participant eligibility, height, body weight, and calculated body mass index (BMI), and a review of prior and concomitant medications. Additional Screening assessments will include a complete physical examination, vital sign measurements, a 12-lead ECG, safety labs (chemistry, hematology, coagulation, urinalysis, see Table 17), serum pregnancy test (WOCBP only), follicle-stimulating hormone (FSH) (if needed to confirm menopausal status), endoscopy (if performed as part of standard of care) +/- biopsy, and determination of stool output (e.g., volume, frequency, formation, and presence of gross blood). Participants will receive corticosteroids and ruxolitinib (if applicable). This information will be recorded in the source documents.

At Screening (i.e., baseline), aGvHD will be characterized according to the MAGIC criteria in Table 10. Participants must have aGvHD that is Grade II, III, or IV, defined in Table 11 to be eligible. AEs will be monitored from the time of consent.

Table 10: GVHD Target Organ Staging (from MAGIC Criteria)

Stage	Skin (active erythema only)	Liver (bilirubin)	Upper GI	Lower GI (stool output/day)
**0**	No active (erythematous) GVHD rash	< 2 mg/dl	No or intermittent nausea, vomiting or anorexia	Adult: < 500 ml/day or <3 episodes/day<br>Child: < 10 ml/kg/day or <4 episodes/day
**1**	Maculopapular rash <25% BSA	2–3 mg/dl	Persistent nausea, vomiting or anorexia	Adult: 500–999 ml/day or 3–4 episodes/day<br>Child: 10–19.9 ml/kg/day or 4–6 episodes/day
**2**	Maculopapular rash 25 – 50% BSA	3.1–6 mg/dl	-	Adult: 1000–1500 ml/day or 5–7 episodes/day<br>Child: 20 – 30 ml/kg/day or 7–10 episodes/day
**3**	Maculopapular rash > 50% BSA	6.1–15 mg/dl	-	Adult: >1500 ml/day or >7 episodes/day<br>Child: > 30 ml/kg/day or >10 episodes/day
**4**	Generalized erythroderma (>50% BSA) plus bullous formation and desquamation > 5% BSA	>15 mg/dl	-	Severe abdominal pain with or without ileus, or grossly bloody stool (regardless of stool volume).

Table 11: Overall aGvHD Clinical Grade (based upon most severe target organ involvement) MAGIC Criteria

aGvHD Clinical Grades	Permitted For Enrollment*
• Grade 0: No stage 1–4 of any organ	No
• Grade I: Stage 1–2 skin without liver, upper GI, or lower GI involvement	No
• Grade II: Stage 3 rash and/or stage 1 liver and/or Stage 1 upper GI and/or Stage 1 lower GI	Yes
• Grade III: Stage 2–3 liver and/or Stage 2–3 lower GI, with Stage 0–3 skin and/or stage 0-1 upper GI	Yes
• Grade IV: Stage 4 skin, liver, or lower GI involvement, with Stage 0–1 upper GI	Yes

*Providing that all study inclusion/exclusion criteria are met

Note: Patients with Stage 3 or 4 liver are excluded from participation in the study

9.1.2 Study Intervention Administration (7-day dosing Cohorts)

Participants who are confirmed to be eligible for the study will be allocated to a treatment cohort. Study intervention (RLS-0071) will be administered as soon as possible after enrollment and completion of the screening activities. Participants will receive RLS-0071 Q8H (± 1 hour) for 7 days (21 consecutive doses total) in addition to corticosteroids and with or without ruxolitinib for treatment of steroid resistant aGvHD. If the participant is discharged, the participant should continue to receive RLS-0071 in the outpatient setting. The timing of dosing should be recorded in the eCRF.

During Study Intervention Administration, participants will be closely monitored. Some assessments will be performed in relation to infusions. Refer to Section 7.6.1 for how to manage infusion-related reactions.

On Day 1 Prior to the first infusion, AEs, concomitant medications, corticosteroids and ruxolitinib (if applicable) will be administered, vital signs will be recorded prior to the start of infusion. Blood samples for PK, PD, anti-drug antibodies (ADA), MAGIC biomarkers markers will be collected any time prior to the first infusion of RLS-0071 on Day 1.

On Day 1 After the first infusion of RLS-0071, AEs, and vital signs (10 minutes post dose). PK samples will be collected at EOI, and within 120 minutes before the next infusion (C_{trough}) (see Table 15), and a PD blood sample will be collected at EOI. In the 3 participants each from Cohort 1a getting the Intense PK sampling: additional PK blood samples will be collected at 1 hour, 2 hours, and 4 hours post dose (see Table 14).

The following procedures will be performed around **each subsequent RLS-0071 treatment day (Days 2-7)**: AEs, concomitant medications, abbreviated physical exam, vital signs (pre dose and 10 minutes post dose for each dose), weight, stool output (e.g., volume, frequency, formation, and presence of gross blood), MAGIC Staging and overall Grade of aGvHD (Table 10 and Table 11), RLS-0071 will be administered Q8H, and corticosteroids and ruxolitinib will be administered.

On **Day 2**, in addition to the procedures done on every RLS-0071 treatment day, safety labs (chemistry, hematology, urinalysis, see Table 17) will be collected and a 12-lead ECG will be done within 60 minutes after the EOI.

On **Day 3**, in addition to the procedures done on every RLS-0071 treatment day, safety labs (chemistry, hematology, urinalysis, see Table 17), blood samples for PD will be collected with the safety labs. In the 3 participants each from Cohort 1a getting the Intense PK sampling only: PK blood samples will be collected within 120 minutes prior to the next dose after either the 7th or 8th or 9th dose of RLS-0071 (see Table 14).

On **Day 7**, in addition to the procedures done on every RLS-0071 treatment day, safety labs (chemistry, hematology, coagulation, urinalysis, see Table 17), 12-lead ECG within 60 minutes after EOI, participant reported outcomes, blood samples for PK (prior to 19th, 20th, or 21st dose, EOI, 2 hours post dose, and 24 hours post dose, see Table 15), blood samples for PD and MAGIC biomarkers will be collected with the safety labs. In the 3 participants each from Cohort 1a getting the Intense PK sampling only: additional PK blood samples will be collected at 1 hour post dose, 4 hour post dose, and 8 hours post dose (see Table 14).

9.1.3 Study Intervention Administration (14-day dosing Cohorts)

Participants who are confirmed to be eligible for the study will be allocated to a treatment cohort. Study intervention (RLS-0071) will be administered as soon as possible after enrollment and completion of the screening activities. Participants will receive RLS-0071 Q8H (± 1 hour) for 14 days (up to 42 consecutive doses total) in addition to corticosteroids and ruxolitinib for treatment of steroid resistant aGvHD. Participants in Cohort 2a or Cohort 3 who improve and are able to leave the hospital after 7 to 13 days of treatment with RLS-0071 will be given the opportunity to roll over into Cohort 4 or Cohort 5 if they are determined to be eligible after discussion between the PI and the participant as well as the Medical Monitor. The outpatient dosing for Cohort 4 and Cohort 5 will be once a day (QD) following discharge to outpatient to completion of Day 14

(approximate range of 28 - 42 total doses of RLS-0071). The timing of dosing should be recorded in the eCRF.

During Study Intervention Administration, participants will be closely monitored. Some assessments will be performed in relation to infusions. Refer to Section 7.6.1 for how to manage infusion-related reactions.

On Day 1 Prior to the first infusion, AEs, concomitant medications, corticosteroids and ruxolitinib (if applicable) will be administered, vital signs will be recorded within 10 minutes prior to the start of infusion. Blood samples for PK, PD, anti-drug antibodies (ADA), MAGIC biomarkers markers will be collected any time prior to the first infusion of RLS-0071 on Day 1.

On Day 1 After the first infusion of RLS-0071, AEs, and vital signs (10 minutes post dose). PK samples will be collected at EOI, and within 120 minutes before the next infusion (C_{trough}) (see Table 15), and a PD blood sample will be collected at EOI. In the 3 participants from Cohort 3 getting the Intense PK sampling: additional PK blood samples will be collected at 1 hour, 2 hours, and 4 hours post dose (see Table 14).

The following procedures will be performed on **every RLS-0071 treatment day (Days 2-14)**: AEs, concomitant medications, abbreviated physical exam, vital signs (pre dose and 10 minutes post dose for each dose), weight, stool output (e.g., volume, frequency, formation, and presence of gross blood), MAGIC Staging and overall Grade of aGvHD (Table 10 and Table 11), RLS-0071 will be administered Q8H. Participants in Cohorts 4 or Cohort 5 who improve and are able to leave the hospital after 7 to 13 days of treatment with RLS-0071 and who are rolled over into Cohort 4 or Cohort 5 will receive RLS-0071 QD following discharge to outpatient to completion of Day 14 (approximate range of 28 - 42 total doses of RLS-0071).

On **Day 2**, in addition to the procedures done on every RLS-0071 treatment day, safety labs (chemistry, hematology, urinalysis, see Table 17) will be collected and a 12-lead ECG will be done within 60 minutes after the EOI.

On **Day 3**, in addition to the procedures done on RLS-0071 treatment day, safety labs (chemistry, hematology, urinalysis, see Table 17), blood samples for PD will be collected with the safety labs. In the 3 participants from Cohort 3 getting the Intense PK sampling : PK blood samples will be collected within 120 minutes prior to the next dose after either the 7th or 8th or 9th dose of RLS-0071 (see Table 14)

On **Day 7**, in addition to the procedures done on every RLS-0071 treatment day, safety labs (chemistry, hematology, coagulation, urinalysis, see Table 17), 12-lead ECG within 60 minutes after EOI, participant reported outcomes, blood samples for PK (prior to 21st dose, EOI, 2 hours post dose, and 24 hours post dose, see Table 15), blood samples for PD and MAGIC biomarkers will be collected with the safety labs. In the 3 participants from Cohort 3 getting the Intense PK sampling : PK blood samples will be collected within 120 minutes prior to the next dose after either the 7th or 8th or 9th dose of RLS-0071 (see Table 14)

On **Day 14**, in addition to the procedures done on every RLS-0071 treatment day, safety labs (chemistry, hematology, coagulation, urinalysis see Table 17), 12-lead ECG within 60 minutes after EOI, participant reported outcomes, and blood samples for PD and MAGIC biomarkers will be collected with the safety labs.

9.1.4 Follow-Up Visits

Participants will have 4 Follow-Up evaluations. If the participant has already been discharged from the hospital, the Follow-Up visit may be performed in an outpatient setting.

On **Day 14 (± 2 days)** [7-day dosing Cohorts only], AEs, concomitant medications, abbreviated physical exam, MAGIC Staging and overall Grade of aGvHD (Table 10 and Table 11), vital signs, weight, safety labs (chemistry, hematology, coagulation, urinalysis see Table 17), participant reported outcomes, blood samples for PD and MAGIC biomarkers, and stool output (e.g., volume, frequency, formation, and presence of gross blood) will be recorded. Corticosteroids (if applicable) and ruxolitinib will continue to be administered.

On **Day 28 (± 4 days)**, AEs, concomitant medications, abbreviated physical exam, MAGIC Staging and overall Grade of aGvHD (Table 10 and Table 11), vital signs, weight, safety labs (chemistry, hematology, coagulation, urinalysis, see Table 17), participant reported outcomes, blood samples for MAGIC biomarkers and ADA, and stool output (e.g., volume, frequency, formation, and presence of gross blood) will be recorded. Corticosteroids (if applicable) and ruxolitinib will continue to be administered.

On **Day 56 (± 7 days)**, AEs, concomitant medications, abbreviated physical exam, MAGIC Staging and overall Grade of aGvHD (Table 10 and Table 11), vital signs, weight, safety labs (chemistry, hematology, urinalysis), participant reported outcomes, and stool output (e.g., volume, frequency, formation, and presence of gross blood) will be recorded. Corticosteroids (if applicable) and ruxolitinib will continue to be administered.

On **Day 180 (± 14 days)**, AEs, concomitant medications, full physical exam, MAGIC Staging and overall Grade of aGvHD (Table 10 and Table 11), vital signs, weight, safety labs (chemistry, hematology, urinalysis, see Table 17), participant reported outcomes, blood samples for ADA (if the participant had ADAs elevated over baseline are detected in the participants' sample at Day 28), and stool output (e.g., volume, frequency, formation, and presence of gross blood) will be recorded. Corticosteroids (if applicable) and ruxolitinib will continue to be administered.

9.1.5 Early Termination

Participants who terminate prior to the Day 180 visit will undergo assessments as outlined above for Day 180.

9.1.6 Unscheduled Assessments

Participants may require additional assessments if they experience an AE. In addition, a participant may need to return to the clinical site for follow up if they experience an AE after discharge from the hospital during the follow-up period.

9.2 Assessment of Efficacy

The following parameters will be captured to assess efficacy and clinical improvement: overall response rate (ORR) at 28 days as primary, and additional measures and timepoints secondarily. The efficacy parameters are described below.

9.2.1 Response to RLS-0071

The primary objective of the study is ORR at 28 days. The response to RLS-0071 treatment at 7, 14, 28, 56, and 180 days will be determined according to the criteria in Table 12. In the subset of participants that have lower GI aGvHD, the response to RLS-0071 treatment will be assessed

according to the criteria in Table 13. The refractoriness of aGvHD defined as at least one of the following:

1) aGvHD increasing in stage in any organ or developing in a new organ after 3 days of RLS-0071; -
2) aGvHD that has not improved in Stage in >1 organ after 7 days of RLS-0071; initiation of additional aGvHD treatment (other than RLS-0071 +/- ruxolitinib); or
3) for after Day 7, participants who progress (e.g., increase in stage in any organ or development in a new organ) during corticosteroid tapering before a 50% decrease in corticosteroids is achieved will be assessed.

Table 12: Response Criteria (GI, skin, and liver)

Endpoint	Definition
Overall Response Rate (ORR)	Proportion of Participants with a CR, VGPR, or PR
Complete Response (CR)	Stage 0 for each organ (skin, liver, and GI tract), and no intervening additional therapy
Very Good Partial Response (VGPR)	Improvement by at least 1 stage in 1 or more organs and<br>Skin: No bullae and no residual erythematous rash involving <25% of the body surface, and<br>Liver: Total serum bilirubin concentration <2 mg/dL or <25% of baseline at enrollment, and<br>Gut: Tolerating food or enteral feeding, predominantly formed stools, no overt gastrointestinal bleeding or abdominal cramping, and no more than occasional nausea or vomiting, and<br>No intervening additional therapy
Partial Response (PR)	Improvement by at least 1 stage in 1 or more organs without progression in other organs, and no intervening additional therapy

Table 13: Response Criteria: Lower GI aGvHD

Endpoint	Definition
Overall Response Rate (ORR)	Proportion of Participants with a CR, VGPR, or PR
Complete Response (CR)	Stage 0 for lower GI aGvHD, and no intervening additional therapy
Very Good Partial Response (VGPR)	Improvement by at least 1 stage in lower GI aGvHD and<br>Tolerating food or enteral feeding, predominantly formed stools, no overt gastrointestinal bleeding or abdominal cramping, and no more than occasional nausea or vomiting, and<br>No intervening additional therapy
Partial Response (PR)	Improvement by at least 1 stage in lower GI aGvHD without progression in other organs, and no intervening additional therapy

9.2.2 Taper of Corticosteroids

The number and percentage of participants who are able to taper corticosteroids to <2 mg/kg methylprednisolone or equivalent or discontinue corticosteroids while maintaining an aGvHD response will be analyzed.

In addition, the overall use of corticosteroids on Day 7, Day 14, Day 28, Day 56, and Day 180 will be analyzed. See Section 7.5.2 for corticosteroid tapering options.

9.2.3 Use of Rescue Medications for Refractory Disease

The number and percentage of participants to initiate additional or alternative treatment(s) for aGvHD (including an increase in steroid dose to >2 mg/kg methylprednisolone equivalent) to treat refractory aGvHD will be analyzed.

9.2.4 Change in Stage of aGvHD

The number and percentage of participants that have a change in stage and the number and percentage of participants to achieve Stage 0 or Stage 1 lower GI, liver, or skin aGvHD according to Table 10 at Day 7, Day 14, Day 28, Day 56, and Day 180 will be analyzed.

The number and percentage of participants who have a change in the overall grade of aGvHD according to Table 11 at Day 7, Day 14, Day 28, Day 56, and Day 180 will be analyzed.

9.2.5 Survival

The overall survival, failure-free survival, and non-relapse mortality will be determined for participants following treatment with RLS-0071.

9.2.6 MAGIC Biomarkers

The Mount Sinai Acute GVHD International Consortium (MAGIC) biomarkers are suppressor of tumorigenesis 2 (ST2), the soluble receptor for the alarmin IL-33, which is shed from multiple cell types in the injured gastrointestinal crypt; and regenerating islet-derived 3-alpha (REG3α) a multifunctional protein that spills into the bloodstream from damaged Paneth cells. The serum concentrations at each timepoint will be summarized for each of the biomarkers.

9.2.7 Pharmacodynamic Biomarkers

The dose response relationship of RLS-0071 (10 mg/kg and 20 mg/kg) on MPO concentration, MPO activity, NETosis marker cell-free DNA concentration, C1q concentration, sC5b-9 concentration, and functional complement assay (IC-MAC) will be assessed.

Markers of acute inflammatory response, such as CRP and selected cytokine levels (e.g., IL-1β, IL-8) may be evaluated.

Refer to the Laboratory Manual for additional information on sample collection, processing, and storage. Samples will be collected as outlined in the SoA (Section 1.2). When samples for PD are collected with PK samples, refer to Section 9.3.2 and the Laboratory Manual for sample processing instructions. Sample analysis and analytical methods will be provided in the Laboratory Manual or other applicable document.

9.2.8 MAGIC Algorithm Probability (MAP)

The Mount Sinai Acute GVHD International Consortium (MAGIC) validated algorithm probability (MAP) that combines serum concentrations of two biomarkers of aGvHD (REG3α and ST2) to generate an estimated probability of 6 month non-relapse mortality for individual participants. The MAP will be summarized.

9.2.9 Participant Outcomes

9.2.9.1 Functional Assessment of Cancer Therapy - Bone Marrow Transplantation (FACT-BMT)

FACT-BMT is a 47-item, valid and reliable measure of five dimensions of quality of life in bone marrow transplant participants, physical well-being; social/family well-being, emotional well-being, functional well-being, and additional concerns.

9.2.9.2 Abdominal Pain

Using a numeric pain rating scale participants will be asked to make 3 pain ratings, on a scale of 0 (no pain) to 10 (worst pain imaginable) what is the intensity of your current, best, or worst pain levels over the past 24 hours. The average of the three readings will be used to represent the participant's pain over the past 24 hours.

9.2.9.3 Volume/Frequency of Diarrhea

The 24 hour volume, frequency, and formation of stool including assessment of gross blood in stool will be recorded daily.

9.2.9.4 Food Tolerance and Use of TPN

Whether the participant is tolerating food and feeding, and the need for TPN will be recorded.

9.2.9.5 Duration of Hospital Stay

The date of hospital admission and discharge for each participant should be recorded in the eCRF.

9.3 Assessments of Pharmacokinetics

Evaluation of the single-dose and multiple-dose PK of RLS-0071 are secondary objectives of the study. Pharmacokinetic evaluation will include parameters for RLS-0071 (Table 16). The PK analysis will be conducted in the PK population (see Section 11.2).

9.3.1 Blood Sample Collection

Serial PK blood samples for determination of RLS-0071 concentrations in plasma will be collected via an indwelling catheter or by individual venipuncture on the opposite arm from the infusion at the timepoints in Table 14 or Table 15. Blood collection tubes should be prechilled. Blood samples should be placed immediately in a wet ice bath for processing in accordance with the sample handling instructions, which will be provided separately. RLS-0071 is not stable in blood or plasma at room temperature. Blood collection in prechilled tubes, held on wet ice, and timely processing to frozen plasma is critical to the integrity of the sample and the study. The PK sampling schedule can be reduced for feasibility, if required.

Table 14: Intense PK Blood Sampling Schedule (3 Participants each in Cohort 1a and Cohort 3)

Study Day	Pre-Dose	Post Dose
Day 1	Pre-dose (any time prior to 1st dose)	EOI (5 min post dose ± 3 min),<br>1 h (±10 min),2 h (±30 min),<br>4 h (±30 min),<br>C_{trough} (up to 120 minutes prior to next dose)
Day 3<br>(around 7th, 8th or 9th dose)	N/A	C_{trough} (up to 120 minutes prior to next dose)
Day 7<br>(around 21st dose)[a]	Pre-dose (up to 120 minutes prior to 21st dose)	EOI (5 min post dose ± 3 min),<br>1 h (±10 min),<br>2 h (±30 min),<br>4 h (±30 min),<br>8 h (±120 min),<br>24 h (±3hr)

C_{trough}=trough plasma concentration; EOI=End of infusion; N/A=not applicable

a. If collection of PK sample is not possible around the 21st dose, the samples can be collected around the 19th or 20th dose. If the PK collection is done around these earlier doses, the 24 hour sample is not required.

Table 15: Sparse PK Blood Sampling Schedule (All Other Participants)

Study Day	Pre-Dose	Post Dose
Day 1	Pre-dose (any time prior to 1st dose)	EOI (5 min post dose ± 3 min),<br>C_{trough} (up to 120 minutes prior to next dose)
Day 7<br>(around 21st dose) [a]	Pre-dose (up to 120 minutes prior to 21st dose)	EOI (5 min post dose ± 3 min),<br>2 h (±30 min),<br>24 h (±3hr)

C_{trough}=trough plasma concentration; EOI=End of infusion; N/A=not applicable

a. If collection of PK samples around the 21st dose is not possible, the samples can be collected around the 19th or 20th dose. If the PK collection is done around these earlier doses, the 24 hour sample is not required.

9.3.2 Sample Processing

Blood samples for PK and PD analysis should be placed immediately in a wet ice bath for processing in accordance with the sample handling instructions, which will be provided separately. RLS-0071 is not stable in blood or plasma at room temperature. Blood collection in prechilled tubes, held on wet ice, and timely processing to frozen plasma is critical to the integrity of the sample and the study.

Blood samples for PK and PD analysis will be processed, separated into aliquots, temporarily stored frozen (≤ -70°C), and shipped frozen as specified in the sample handling instructions. Samples will be stored in accordance with the terms included in the informed consent form (ICF). The bioanalytical laboratory will store unused PK samples until informed by the Sponsor that they may be destroyed.

9.3.3 Sample Analysis and Analytical Methods

Plasma concentrations for RLS-0071 will be determined using a robust, highly sensitive validated bioanalytical method for human plasma. Refer to Table 16. for the PK parameters to be analyzed.

Where possible, all samples from each participant will be analyzed on the same standard curve. Quality control samples will be distributed through each batch of study samples assayed. Samples with drug concentrations greater than the upper limit of quantitation of the assay range will be diluted with the appropriate drug-free biological fluid and re-assayed; those that are below the lower limit of this range will be reported as below the lower limit of quantitation.

Unacceptable values attributable to bioanalytical reasons will be determined according to the standard operating procedures (SOPs) of the bioanalytical facility. Such re-assayed samples will be termed “repeats.” The method of re-assay and the acceptance criteria for selecting which value to report for the re-assayed samples will also follow the SOPs of the bioanalytical facility. All cases of re-assay will be detailed in the final bioanalytical report. No samples will be repeated for reasons relating to PK analysis. To be compliant with regulatory requirements and to establish the reproducibility of the assay with incurred samples, at least 10% of the total analyzable study samples will be selected and re-assayed. The replicate measurement will not be averaged with the original one, but both values will be presented in the bioanalytical report with the initial value being used for PK calculations. The concentrations of the original and replicate samples will be tabulated, along with the percentage difference between the 2 values.

Table 16: Pharmacokinetic Parameters Derived for RLS-0071 Concentrations

Variable	Definition
C_{max}	Maximum plasma concentration observed.
T_{max}	Time to reach C_{max}. If the maximum observed concentration value occurs at more than one time point, T_{max} is defined as the first time point with this value.
AUC0-τ	Area under the plasma concentration-time curve during the dosing interval, calculated by the linear trapezoidal rule
AUC_{0-48}	Area under the plasma-concentration-time curve from 0 to 48 hours, calculated by the linear trapezoid rule.
C_{avg}	average plasma drug concentration observed during multiple-dose administration
C_{trough}	Trough concentrations prior to dose administration
λ_x	Apparent first-order terminal elimination rate constant, calculated from a semi-log plot of the plasma concentration-time curve and by linear least-squares regression analysis using the maximum number of points in the terminal log-linear phase (e.g., 3 or more nonzero plasma concentrations)
V_D/F	Apparent total volume of distribution, $V_D/F = \left(\frac{Dose}{\lambda_Z * AUC_{0-\infty}}\right)$
CL/F	Apparent total body clearance, $CL/F = \left(\frac{Dose}{AUC_{0-\infty}}\right)$ Note: Weight-adjusted CL/F (CL/F/kg) will also be calculated
$T_{1/2}$	Apparent first-order terminal elimination half-life, calculated as ln(2)/λz

9.4 Assessment of Immunogenicity

Evaluation of the immunogenicity of RLS-0071 through measurement of ADAs before and after IV administration will inform the safety evaluation of RLS-0071.

Antibodies to RLS-0071 will be evaluated in blood samples collected from all participants according to the SoA (see Section 1.2). If a participant has ADAs elevated over baseline at the Day 28 visit, they have a second sample collected Day 180 (± 14 days).

These samples will be tested by the Sponsor or Sponsor's designee. Blood samples will be screened for antibodies binding to RLS-0071. RLS-0071 itself comprises a PEG domain conjugated to a peptide. Samples that have antibodies directed to RLS-0071 will be further analyzed to determine the domain specificity of their binding (anti-PEG versus anti-peptide) and the titer of confirmed positive samples will be reported. Other analyses may be performed to verify the stability of antibodies to RLS-0071 and/or further characterize the immunogenicity of RLS-0071.

The detection and characterization of antibodies to RLS-0071 will be performed using a validated assay method by or under the supervision of the Sponsor. Antibodies may be further characterized and/or evaluated for their ability to neutralize the activity of the study intervention. ADA analyses will conform with the FDA Guidance for Industry: Immunogenicity Testing of Therapeutic Protein Products - Developing and Validating Assays for Anti-Drug Antibody Detection (February 2019). An ADA will be considered positive prior to initiation of dosing (baseline) if it exceeds a relevant population parameter. However, an ADA will only be considered positive at subsequent timepoints (post dose) if it is confirmed to exceed the baseline titer. The Sponsor is aware that some participants may have pre-existing anti-PEG antibodies and increased titers following dosing will be participant to follow-up and reporting as ADA-positives.

9.5 Assessment of Safety

9.5.1 Safety Parameters

Clinical and laboratory status will be monitored throughout the study period. Assessments of safety will include the incidence and severity of AEs including serious adverse events (SAEs), clinical laboratory tests (, clinical chemistry, hematology, and urinalysis), ADA, physical examination, vital signs, 12-lead ECG, radiography, and concomitant medications.

All AEs will be collected from the time of consent until the completion of the Day 180 visit. Adverse event grading will be defined by the CTCAEv5.0.

Immediate safety concerns should be discussed with the Sponsor's Medical Monitor as soon as possible upon occurrence or awareness to determine if the participant should continue or discontinue study intervention.

A DSMB will be established to review available safety, tolerability, PK, and PD data. The details on the DSMB are provided in Section 11.5.

Planned time points for all safety assessments are provided in the SoA (Section 1.2).

9.5.2 Physical Examination

The Investigator will conduct complete physical examinations and abbreviated physical examinations in accordance with the SoA (see Section 1.2).

- A complete physical examination will include, at a minimum, assessments of the skin, head, eyes, ears, nose, throat (HEENT), neck, thyroid, lungs, heart, abdomen, lymph nodes, and extremities.
- An abbreviated physical examination will include, at a minimum, assessments of the HEENT, heart, lungs, abdomen, and lower extremities.
- Investigators should pay special attention to clinical signs related to previous serious illnesses.
- If the complete physical examination performed as part of Screening is within 6 hours of the first infusion, an abbreviated physical examination is not needed before the first infusion. In this case, the Screening physical examination will serve as baseline.
- Height and body weight will be measured in accordance with the SoA (see Section 1.2).

9.5.2.1 Vital Signs

Vital signs (including heart rate, respiration rate, blood pressure [systolic and diastolic], and temperature) will be monitored. Vital signs will be recorded in accordance with the SoA (see Section 1.2) as follows:

- Vital signs will be recorded before blood collection for laboratory tests.
- Routine blood pressure and heart rate measurements should be preceded by at least 5 minutes of rest for the participant. Where feasible, the participant's resting condition (whether sitting or supine) should be the same for each measurement.
- On the days of study intervention administration, vital signs will be recorded pre dose and 10 minutes post dose for each dose (± 10 minutes).

9.5.2.2 Electrocardiogram

Electrocardiograms will be conducted as indicated in the SoA (Section 1.2).

- 12-lead ECGs will be obtained using an ECG machine that automatically calculates the heart rate and measures PR, QRS, QT, and QT interval corrected for heart rate using Fridericia's formula (QTcF) intervals.
- ECGs will be performed at screening and within 60 minutes after the end of the infusion on Days 2, 7, (14, if received 14 days of dosing).

9.5.2.3 Stool Output

Stool output will be measured in mL/24hours as indicated in the SoA (Section 1.2).

9.5.2.4 Clinical Safety Laboratory Assessments

Safety laboratory assessments (CBC, chemistry, hematology, urinalysis) and serum pregnancy tests will be conducted according to the institutional procedures (see Table 17 for details of clinical safety laboratory parameters).

- See the SoA (Section 1.2) for the timing and frequency of sampling.
- If the Screening clinical safety laboratory assessments are performed within 6 hours before the first infusion, they do not need to be repeated. In this case, the Screening values may be used as baseline.

- Pregnancy tests are required for WOCBP only. Refer to Section 7.4.5 for the definition of WOCBP. If needed, FSH level may be determined at Screening to confirm menopausal status.

Table 17: Clinical Laboratory Assessments

**Laboratory Assessments**	**Parameters**		
Hematology	Hemoglobin<br>Hematocrit<br>Platelet count	Red blood cell indices:<br>Erythrocyte count<br>Mean corpuscular volume<br>Mean corpuscular hemoglobin concentration<br>%Reticulocytes	White blood cell count with differential (percentage and absolute number):<br>Neutrophils<br>Lymphocytes<br>Monocytes<br>Eosinophils<br>Basophils
Clinical Chemistry	Sodium<br>Potassium<br>Chloride<br>CO_2 (bicarbonate)<br>Glucose (nonfasting)	Blood urea nitrogen<br>Creatine[a]<br>Total protein<br>Albumin<br>Bilirubin, total and direct<br>Aspartate aminotransferase<br>Alanine aminotransferase<br>Lactate dehydrogenase<br>Alkaline phosphatase	Calcium<br>Amylase
Coagulation	Prothrombin time (PT)<br>International normalized ratio (INR)<br>Activated partial thromboplastin, time (APTT)		
Routine Urinalysis	Specific gravity<br>pH, glucose, blood, ketones by dipstick[d]<br>Microscopic examination (if blood or protein is abnormal)		
Other Screening Tests	Highly sensitive serum human chorionic gonadotropin pregnancy test (as needed for WOCBP)[b]<br>Follicle-stimulating hormone [c]		

Abbreviations: ET = early termination.
[a] Serum creatinine will be used to estimate the glomerular filtration rate.
[b] A serum pregnancy test will be conducted at Screening.
[c] Follicle-stimulating hormone level may be determined at Screening to confirm menopausal status.
[d] Urinalysis lab may also be used, rather than dipstick.

10. Adverse Events and Serious Adverse Events

In this study, AEs will be reported for all participants from the time of consent until the completion of the Day 180 visit. SAEs will be reported for all participants (enrolled and not enrolled) from the time of consent. Adverse events reported from the time of consent to start of study treatment will be recorded as pre-treatment AEs. Treatment emergent- AEs (TEAEs) will be evaluated from the first administration of study drug until the Follow-up/EOS visit or up to a 30day follow-up period for AEs deemed- related to treatment. Adverse events that are ongoing at the EOS visit will be marked as Not Recovered/Not resolved on the AE eCRF page (see Section 10.7).

All spontaneously volunteered and enquired for, as well as observed AEs, will be recorded in the participant's medical records and the eCRF.

10.1 Definition of Adverse Events (AEs)

An AE is any untoward medical occurrence in a participant or clinical study participant, temporally associated with the use of study intervention, whether or not considered related to the study intervention.

NOTE: An AE can therefore be any unfavorable and unintended sign (including an abnormal laboratory finding), symptom, or disease (new or exacerbated) temporally associated with the use of study intervention.

Events meeting the definition of an AE include:

- Any abnormal laboratory test results (hematology, clinical chemistry, or urinalysis) or other safety assessments (e.g., ECG, radiological scans, vital signs measurements), including those that worsen from baseline, considered clinically significant in the medical and scientific judgment of the Investigator (i.e., not related to progression of underlying disease).
- Exacerbation of a chronic or intermittent pre-existing condition including either an increase in frequency and/or intensity of the condition.
- New conditions detected or diagnosed after study intervention administration even though it may have been present before the start of the study.
- Signs, symptoms, or the clinical sequelae of a suspected drug-drug interaction.
- Signs, symptoms, or the clinical sequelae of a suspected overdose of either study intervention or a concomitant medication. Overdose per se will not be reported as an AE/SAE unless it is an intentional overdose taken with possible suicidal/self-harming intent. Such overdoses should be reported regardless of sequelae.

Events that do not meet the definition of an AE include:

- The disease/disorder being studied or expected progression, signs, or symptoms of the disease/disorder being studied, unless more severe than expected for the participant's condition.
- Medical or surgical procedure (e.g., endoscopy, appendectomy): the condition that leads to the procedure is the AE.

- Situations in which an untoward medical occurrence did not occur (social and/or convenience admission to a hospital).
- Anticipated day-to-day fluctuations of pre-existing disease(s) or condition(s) present or detected at the start of the study that do not worsen.

If there is evidence of an AE through report or observation, the Investigator or designee will evaluate further and record the following information:

- Time of onset and resolution
- Severity
- Seriousness
- Causality/relation to study treatment
- Action taken regarding study drug
- Action taken regarding AE
- Outcome

10.2 Definition of Serious Adverse Event

An SAE is an AE occurring during any study phase (i.e., baseline, treatment, or follow-up), and at any dose of the study drug (active), that fulfils one or more of the following:

1. Results in death including spontaneous abortion or fetal death.
2. **Is life-threatening.** The term “life-threatening” in the definition of “serious” refers to an event in which the participant was at risk of death at the time of the event. It does not refer to an event, which hypothetically might have caused death, if it were more severe.
3. **Requires inpatient hospitalization or prolongation of existing hospitalization.** In general, hospitalization signifies that the participant has been detained (usually involving at least an overnight stay) at the hospital or emergency ward for observation and/or treatment that would not have been appropriate in the physician’s office or outpatient setting. Complications that occur during hospitalization are AEs. If a complication prolongs hospitalization or fulfills any other serious criteria, the event is serious. When in doubt as to whether “hospitalization” occurred or was necessary, the AE should be considered serious.
4. **Results in persistent or significant disability or incapacity.** The term disability means a substantial disruption of a person’s ability to conduct normal life functions.

This definition is not intended to include experiences of relatively minor medical significance such as uncomplicated headache, nausea, vomiting, diarrhea, influenza, and accidental trauma (e.g., sprained ankle) which may interfere with or prevent everyday life functions but do not constitute a substantial disruption.

5. Is a congenital abnormality or birth defect.
6. **Medically Important Event:** Medical or scientific judgment should be exercised in deciding whether SAE reporting is appropriate in other medically important events that may not be immediately life-threatening or result in death or hospitalization but may jeopardize the participant or may require medical or surgical intervention to

prevent one of the other outcomes listed in the above definition. These events should usually be considered serious.

Examples of such events include invasive or malignant cancers, intensive treatment in an emergency room or at home for allergic bronchospasm, blood dyscrasias or convulsions that do not result in hospitalization, or development of drug dependency or drug abuse.

An AE is considered “life-threatening” if, in the opinion of either the Investigator or the Sponsor, its occurrence places the participant at immediate risk of death. It does not include an AE that, had it occurred in a more severe form, might have caused death.

10.2.1 Notification of Serious Adverse Event

The primary mechanism for reporting an SAE will be the electronic data collection tool.

If the electronic system is unavailable, then the site will use the paper SAE data collection tool (see next section) in order to report the event within 24 hours.

The site will enter the SAE data into the electronic system as soon as it becomes available.

After the study is completed at a given site, the electronic data collection tool will be taken off-line to prevent the entry of new data or changes to existing data.

If a site receives updated data on a previously reported SAE after the electronic data collection tool has been taken off-line, then the site can report this information on a paper SAE form.

Contacts for SAE reporting can be found in the study materials.

10.3 Recording Adverse Events

When an AE/SAE occurs, it is the responsibility of the Investigator to review all documentation (e.g., hospital progress notes, laboratory reports, and diagnostics reports) related to the event.

The Investigator will then record all relevant AE/SAE information in the source documents.

It is not acceptable for the Investigator to send photocopies of the participant's medical records in lieu of completion of the appropriate source document.

There may be instances when copies of medical records for certain cases are requested. In this case, all participant identifiers, with the exception of the participant number, will be redacted on the copies of the medical records before submission.

The Investigator will attempt to establish a diagnosis of the event based on signs, symptoms, and/or other clinical information. Whenever possible, the diagnosis (not the individual signs/symptoms) will be documented as the AE/SAE.

10.4 Severity of an AE/SAE

The Investigator will make an assessment of intensity for each AE and SAE reported during the study and assign a Grade of 1 to 5 in accordance with CTCAEv5.0. For terms not included in CTCAE, the Investigator will assign 1 of the following categories:

- **Grade 1 Mild:** asymptomatic or mild symptoms; clinical or diagnostic observations only; intervention not indicated.
- **Grade 2 Moderate:** minimal, local, or noninvasive intervention indicated; limiting age-appropriate instrumental activities of daily living.

- **Grade 3 Severe or medically significant but not immediately life-threatening;** hospitalization or prolongation of hospitalization indicated; disabling; limiting self-care activities of daily living.
- Grade 4 Life-threatening consequences; urgent intervention indicated.
- **Grade 5 Death** related to AE.

An event is defined as "serious" when it meets at least 1 of the predefined outcomes as described in the definition of an SAE, NOT when it is rated as severe.

10.5 Causal Relationship of an AE/SAE

The Investigator is obligated to assess the relationship between study intervention and each occurrence of each AE/SAE.

- A "reasonable possibility" of a relationship conveys that there are facts, evidence, and/or arguments to suggest a causal relationship, rather than a relationship cannot be ruled out.

The Investigator will use clinical judgment to determine the relationship.

Alternative causes, such as underlying disease(s), concomitant therapy, and other risk factors, as well as the temporal relationship of the event to study intervention administration will be considered and investigated.

The Investigator will also consult the Investigator's Brochure and/or Product Information, for marketed products, in his/her assessment.

For each AE/SAE, the Investigator **must** document in the medical notes that he/she has reviewed the AE/SAE and has provided an assessment of causality.

There may be situations in which an SAE has occurred, and the Investigator has minimal information to include in the initial report. However, it is very important that the Investigator always make an assessment of causality for every event before the initial transmission of the SAE data.

The Investigator may change his/her opinion of causality in light of follow-up information and send a SAE follow-up report with the updated causality assessment.

The causality assessment is one of the criteria used when determining regulatory reporting requirements.

Relationship with the study intervention will be assessed using the criteria in Table 18.

Table 18: Causality Categories

Causality Term	Assessment Criteria
Certain	• Event or laboratory test abnormality, with plausible time relationship to drug intake.<br>• Cannot be explained by disease or other drugs.<br>• Response to withdrawal plausible (pharmacologically, pathologically).<br>• Event definitive pharmacologically or phenomenologically (i.e., an objective and specific medical disorder or a recognized pharmacological phenomenon).<br>• Rechallenge satisfactory, if necessary.
Probable/Likely	• Event or laboratory test abnormality, with reasonable time relationship to drug intake.<br>• Unlikely to be attributed to disease or other drugs.<br>• Response to withdrawal clinically reasonable.<br>• Rechallenge not required.
Possible	• Event or laboratory test abnormality, with reasonable time relationship to drug intake.<br>• Could also be explained by disease or other drugs.<br>• Information on drug withdrawal may be lacking or unclear.
Unlikely	• Event or laboratory test abnormality, with a time to drug intake that makes a relationship improbable (but not impossible).<br>• Disease or other drugs provide plausible explanations.
Unrelated	• Event or laboratory test abnormality, with no time relationship to drug intake.<br>• Disease or other drugs provide plausible explanations.
Conditional/Unclassified	• Event or laboratory test abnormality.<br>• More data for proper assessment needed, or<br>• Additional data under examination.
Unassessable/Unclassifiable	• Report suggesting an adverse reaction.<br>• Cannot be judged because information is insufficient or contradictory.<br>• Data cannot be supplemented or verified.

10.6 Action Taken with Investigational Products

Should the Investigator need to alter the administration of the study drug from the procedure described in the protocol due to the well-being and safety of the participant, then the action taken will be recorded on the AE eCRF page as one of the following options:

- Dose Reduced
- Drug Interrupted
- Drug Withdrawn
- Not Applicable
- Other

10.7 Outcome

Outcome of an AE/SAE will be recorded on the AE eCRF as follows:

- Recovered / Resolved
- Recovering / Resolving
- Recovered / Resolved with Sequelae
- Not Recovered / Not Resolving
- Fatal
- Unknown

10.8 Follow-up of Adverse Events and Serious Adverse Events

The Investigator is obligated to perform or arrange for the conduct of supplemental measurements and/or evaluations as medically indicated or as requested to elucidate the nature and/or causality of the AE or SAE as fully as possible. This may include additional laboratory tests or investigations, histopathological examinations, or consultation with other health care professionals.

If a participant dies during participation in the study or during a recognized follow-up period, the Investigator will provide a copy of any post-mortem findings including histopathology.

New or updated information will be recorded in the originally completed eCRF.

The Investigator will submit any updated SAE data to the Sponsor within 24 hours of receipt of the information.

10.9 Adverse Events that are Expected

Below are adverse events or disease manifestations that can be expected in this study. All AEs and SAEs should be recorded in the study documents and reported as required in Section 10.2.1 and Section 10.3 even if they are listed in the sections below. The information below can be used to help determine the causal relationship of an AE or SAE as described in Section 10.5.

10.9.1 Expected Adverse Events due to RLS-0071

The most common adverse events that have been reported with RLS-0071 are:

- Headache
- Catheter site swelling
- Dizziness
- Constipation
- Diarrhea
- Fatigue
- Somnolence
- Vessel puncture site bruise

All AEs reported have been mild to moderate (Grade 1-2). To date, the only AEs that have been judged by the investigator to be due to RLS-0071 was headache in 1 participant. All other AEs were considered not or unlikely related to RLS-0071. No SAEs or deaths have been reported on

RLS-0071 clinical trials. See the RLS-0071 Investigator Brochure for a complete listing of adverse events reported on each study.

10.9.2 Expected Adverse Events due to Ruxolitinib

In acute graft-versus-host disease, the most common hematologic adverse reactions with ruxolitinib (incidence > 50%) are anemia, thrombocytopenia, and neutropenia.

The most common nonhematologic adverse reactions with ruxolitinib (incidence > 50%) are infections (pathogen not specified) and edema.

For more information and a complete list of adverse events, see the Jakafi (ruxolitinib) prescribing information (Incyte Corporation, 2023).

10.9.3 Expected aGvHD Manifestations

- Skin: rash (especially an extensive rash); generalized erythroderma plus bullous formation and desquamation
- Liver: elevated bilirubin
- Upper GI: nausea, vomiting, or anorexia
- Lower GI: diarrhea (especially of high or increased volume or frequency; severe abdominal pain (without ileus); grossly bloody stool

10.10 Adverse Events of Special Interest

Due to the modulation of inflammatory responses, the Adverse Events of Special Interest for RLS-0071 is invasive infection. Although no invasive infections have been reported to date in the two clinical trials conducted with RLS-0071 thus far, these are of special interest. Definition: A diagnosis of an invasive infection (e.g., bloodstream infection, abscess, pneumonia, device related infection, etc.) due to bacteria, fungus, or virus with microbiologic or molecular detection confirmation.

10.11 Pregnancy

If a participant in the study becomes pregnant, RLS-0071 will be discontinued and then the pregnancy will be followed, and the outcome will be obtained. If the participant’s partner becomes pregnant, then the pregnancy will be followed and the outcome will be obtained, if consented. The pregnancy should be reported to the CRO/Sponsor on the pregnancy surveillance form.

11. Statistics

11.1 Sample Size Determination

There was no formal sample size determination. The sample size was selected based on clinical considerations. The data will be summarized with descriptive statistics.

11.2 Populations for Analysis

The following Populations will be analyzed.

Safety Population: All participants who receive at least 1 dose of study treatment.

Intent to Treat (ITT) Population: Any participant enrolled in one of the Cohorts regardless of treatment received.

Modified ITT (mITT) Population: All participants who received at least 1 dose of study treatment and have at least 1 post baseline efficacy measurement.

Per Protocol (PP) Population: Participants that receive all intended doses of study treatment with no major protocol deviations.

Modified Per Protocol (mPP) Population: Any participant with Day 7 ORR data.

Pharmacokinetic (PK) Population: Any participant with sufficient PK samples for valid PK analysis and without any major protocol deviations that affect the PK assessment.

11.3 Statistical Analyses

The statistical analysis plan (SAP) will be finalized prior to database lock and will include a more technical and detailed description of the statistical analyses described in this section. The SAP will supersede any information in the protocol. This is an open label study and while there is no concurrent non-RLS-0071 control group, the study efficacy data will be evaluated in the context of historical control data, including those studies evaluated as part of the ruxolitinib development program for steroid-refractory aGvHD.

This section is a summary of the planned statistical analyses including primary and key secondary endpoints.

11.3.1 Primary Endpoints

The primary objectives of this study, to demonstrate the safety and tolerability of RLS-0071 and to determine the overall response rate (ORR) of RLS-0071 at Day 28, will be assessed by the endpoints described in Section 4.2.1.

All safety data will be summarized by Cohort. AEs will be coded using the most recent version of MedDRA.

The frequency of TEAEs, TEAEs by maximum intensity, TEAEs by relationship to study intervention, TEAEs leading to withdrawal from the study, SAEs and deaths will be summarized by system organ class, preferred term, and treatment group. Listings will also be provided for deaths, SAEs, and TEAEs leading to withdrawal from the study.

An AE is defined as treatment emergent if the first onset or worsening is after the first administration of study intervention (RLS-0071) and not more than 30 days after the last administration of study intervention.

ORR will be determined according to the criteria defined in Table 12 and Table 13.

All safety analyses will be based on the Safety Population. All efficacy analyses will be done in the ITT Population, mITT Population, PP Population, and mPP Population.

11.3.2 Secondary Endpoints

The secondary endpoints described in Section 4.1.2 will be used to assess efficacy of RLS-0071.

All efficacy endpoints will be based on the ITT Population, mITT Population, PP Population, and mPP Population and will be analyzed with descriptive statistics.

11.3.3 Pharmacokinetic Endpoints

One of the secondary endpoints in Section 4.1.2 is to evaluate the PK of RLS-0071. PK parameters are defined in full in Section 9.3. No formal statistical analyses of PK parameters are planned. Plasma concentrations and single-dose and multiple-dose PK parameters for RLS-0071 will be summarized by cohort using descriptive statistics (number of participants, arithmetic mean, geometric mean, % coefficient of variation (CV), geometric %CV, standard deviation (SD), median, and minimum and maximum values).

For noncompartmental analysis, plasma concentrations will be listed and summarized at each time point using descriptive statistics; graphical representations will also be provided.

If data permit, modeling will be attempted to further explore the PK/PD (i.e., exposure-response) relationship. A statistical relationship between the PK/PD of RLS-0071 will also be characterized, as data permit.

All PK endpoints will be based on the PK Population.

11.3.4 Exploratory Endpoints

Exploratory objective to evaluate the PD of RLS-0071 following single and multiple doses will be assessed by the endpoints in Section 4.2.3.

PD parameters will be summarized by cohort using descriptive statistics (number of participants, arithmetic mean, SD, median, and minimum and maximum values).

Exploratory analyses will be performed on the ITT Population, mITT Population, PP Population, and mPP Population.

11.4 Interim Analysis

There is no formal interim analysis planned.

11.5 Data Safety Monitoring Board (DSMB)

An independent DSMB will be established to review available safety, tolerability, as well as available PK and PD data for the duration of the study and to make decisions regarding dose escalation and continuation of the study. Members will be separate and independent of study personnel participating in the study and will have appropriate expertise to contribute to the interpretation of the data from the study. The DSMB makes recommendations, based on safety, to the Sponsor to continue, interrupt, or stop the trial.

The full scope and responsibilities of the DSMB will be described in the DSMB Charter per the Guidance for Clinical Trial Sponsors: Establishment and Operation of Clinical Trial Data Monitoring Committees.

12. Direct Access to Source Data/Documents

Source documents provide evidence for the existence of the participant and substantiate the integrity of the data collected. Source documents are filed at the Investigator's site.

Data entered in the electronic data capture (EDC) that are transcribed from source documents must be consistent with the source documents or the discrepancies must be explained. The Investigator may need to request previous medical records or transfer records, depending on the study. Also, current medical records must be available.

Examples of acceptable source documentation include, but are not limited to, hospital records, clinic and office charts, laboratory notes, and recorded data from automated instruments, memoranda, and pharmacy dispensing records.

13. Quality Assurance and Quality Control

All participant data relating to the study will be recorded on applicable source documents or captured in an Electronic Medical Record unless transmitted to the Sponsor or designee electronically (e.g., laboratory data). The Investigator is responsible for verifying that data entries in the EDC system are accurate and correct by physically or electronically signing the eCRF.

The Investigator must maintain accurate documentation (source data) that supports the information entered into the EDC.

The Investigator must permit study-related monitoring, audits, IRB/IEC review, and regulatory agency inspections and provide direct access to source data documents.

The Sponsor or designee is responsible for the data management of this study including quality checking of the data.

The Sponsor assumes accountability for actions delegated to other individuals (e.g., CROs).

Study monitors will perform ongoing source data verification to confirm that data entered into the EDC by authorized site personnel are accurate, complete, and verifiable from source documents; that the safety and rights of participants are being protected; and that the study is being conducted in accordance with the currently approved protocol and any other study agreements, ICH GCP, and all applicable regulatory requirements.

Records and documents, including signed informed consent forms (ICFs) and assent forms, pertaining to the conduct of this study must be retained by the Investigator for 2 years after the last approval of a marketing application in an ICH region, and until there are no pending or contemplated marketing applications in an ICH region or at least 2 years after the formal discontinuation of clinical development of the investigational product. No records may be destroyed during the retention period without the written approval of the Sponsor. No records may be transferred to another location or party without written notification to the Sponsor.

14. Ethics Description and Ethical Considerations

This study will be conducted in accordance with the protocol and with the following:

- Consensus ethical principles derived from international guidelines including the Declaration of Helsinki and Council for International Organizations of Medical Sciences (CIOMS) International Ethical Guidelines.
- Applicable International Council for Harmonisation of Technical Requirements for Pharmaceuticals for Human Use (ICH) Good Clinical Practice (GCP) Guidelines.
- Applicable laws and regulations.

The protocol, protocol amendments, ICF, Investigator Brochure, and other relevant documents (e.g., advertisements) must be submitted to an IRB/IEC by the Investigator and reviewed and approved by the IRB/IEC before the study is initiated, or before such documents (e.g., advertisements) are used for the study.

Any amendments to the protocol will require IRB/IEC approval before implementation of changes made to the study design, except for changes necessary to eliminate an immediate hazard to study participants.

The Investigator will be responsible for the following:

- Providing written summaries of the status of the study to the IRB/IEC annually or more frequently in accordance with the requirements, policies, and procedures established by the IRB/IEC.
- Notifying the IRB/IEC of SAEs or other significant safety findings as required by IRB/IEC procedures.
- Providing oversight of the conduct of the study at the site and adherence to requirements of 21 Code of Federal Regulations (CFR), ICH guidelines, the IRB/IEC, European regulation 536/2014 for clinical studies (if applicable), and all other applicable local regulations.

14.1 Informed Consent

Participants or their legally authorized representative must provide informed consent before any study-specific procedure is conducted. Children under the age of 18 must provide assent.

The informed consent process will be as follows:

- The Investigator or his/her representative will explain the nature of the study to the participant and answer all questions regarding the study.
- Participants must be informed that their participation is voluntary. Participants will be required to sign a statement of informed consent that meets the requirements of 21 CFR 50, local regulations, ICH guidelines, Health Insurance Portability and Accountability Act (HIPAA) requirements, where applicable, and the IRB/IEC or study center. If the participant is under 18 years of age, their parent or legal guardian will sign the ICF, and the participant will sign the assent form.
- The medical record must include a statement that written informed consent was obtained before the participant was enrolled in the study and the date the written consent/assent

was obtained. The authorized person obtaining the informed consent must also sign the ICF.

- Participants must be re-consented to the most current version of the ICF(s) during their participation in the study.
- A copy of the ICF(s) and assent (if applicable) must be provided to the participant.

15. Data Handling and Recordkeeping

15.1 Dissemination of Clinical Study Data

All information concerning RLS-0071, the Sponsor's operations, patent applications, formula, manufacturing processes, basic scientific data, and formulation information supplied by ReAlta or its designee to the Investigator, and not previously published, is considered confidential and remains the sole property of . Case report forms also remain the property of ReAlta. The Investigator agrees to use this information for purposes of study execution through finalization and will not use it for other purposes without the written consent of the Sponsor.

The information developed in this study will be used by ReAlta in connection with the continued development of RLS-0071 and thus may be disclosed as required to other clinical investigators or government regulatory agencies.

The information generated by this study is the property of ReAlta. Publication or other public presentation of RLS-0071 data resulting from this study requires prior review and written approval of ReAlta. Abstracts, manuscripts, and presentation materials should be provided to ReAlta for review and approval at least 30 days prior to the relevant submission deadline.

The final clinical study report (CSR) will include a summary of the study results based on a statistical evaluation and clinical assessment of the protocol-defined endpoints. The final CSR may be submitted to the regulatory authorities.

16. Financial Statement

Investigators and sub-investigators will provide the Sponsor with sufficient, accurate financial information as requested to allow the Sponsor to submit complete and accurate financial certification or disclosure statements to the appropriate regulatory authorities. Investigators are responsible for providing information on financial interests during the course of the study and for 1 year after completion of the study.

17. Publication Policy

The results of this study may be published or presented at scientific meetings. If this is foreseen, the Investigator agrees to submit all manuscripts or abstracts to the Sponsor at least 30 days prior to the relevant submission deadline. This allows the Sponsor to protect proprietary information and to provide comments. All publications, manuscripts or abstracts, or presentations require the written approval of ReAlta.

The Sponsor will comply with the requirements for publication of study results.

Authorship will be determined by mutual agreement and in line with the International Committee of Medical Journal Editors authorship requirements.

18. References

Crooks, V., Waller, S., Smith, T*., et al.* (1991). The use of the Karnofsky Performance Scale in determining outcomes and risk in geriatric outpatients. *J Gerontol*, *46*(4), M139-144. https://doi.org/10.1093/geronj/46.4.m139

de Haan, R., Aaronson, N., Limburg, M*., et al.* (1993). Measuring quality of life in stroke. *Stroke*, *24*(2), 320-327. https://doi.org/10.1161/01.str.24.2.320

Finke, J., Bethge, W. A., Schmoor, C., Ottinger, H. D., Stelljes, M., Zander, A. R., et al. (2009). Standard graft-versus-host disease prophylaxis with or without anti-T-cell globulin in haematopoietic cell transplantation from matched unrelated donors: a randomised, open-label, multicentre phase 3 trial. *Lancet Oncol.*, *10*(9), 855-864.

Gloude, N. J., Khandelwal, P., Luebbering, N*., et al.* (2017). Circulating dsDNA, endothelial injury, and complement activation in thrombotic microangiopathy and GVHD. *Blood*, *130*(10), 1259-1266. https://doi.org/10.1182/blood-2017-05-782870

Hollen, P. J., Gralla, R. J., Kris, M. G*., et al.* (1994). Measurement of quality of life in patients with lung cancer in multicenter trials of new therapies. Psychometric assessment of the Lung Cancer Symptom Scale. *Cancer*, *73*(8), 2087-2098. https://doi.org/10.1002/1097-0142(19940415)73:8<2087::aid-cncr2820730813>3.0.co;2-x

HRSA. (2022, 14 April 2022). Transplant Activity Report. Health Resources & Services Admnistration: Blood Stem Cell. Retrieved 6 March 2023 from https://bloodstemcell.hrsa.gov/data/donation-and-transplantation-statistics/transplant-activity-report#summary

Hulsdunker, J., Ottmuller, K. J., Neeff, H. P*., et al.* (2018). Neutrophils provide cellular communication between ileum and mesenteric lymph nodes at graft-versus-host disease onset. *Blood*, *131*(16), 1858-1869. https://doi.org/10.1182/blood-2017-10-812891

Incyte Corporation. (2023, 01/2023). Highlights of Prescribing Informaition: JAKAFI (Ruxolitinib) tablets. https://www.jakafi.com/pdf/prescribing-information.pdf

Kanakry, C. G., Tsai, H.L., Bolaños-Meade, J. et al. (2014). Single-agent GVHD prophylaxis with posttransplantation cyclophosphamide after myeloablative, HLA-matched BMT for AML, ALL, and MDS. *Blood*, *124*, 3817–3827.

Kröger, N., Solano, C., Wolschke, C., Bandini, G., Patriarca, F., Pini, M., et al. (2016). Antilymphocyte Globulin for Prevention of Chronic Graft-versus-Host Disease. *N Engl J Med*, *374*, 43-53.

Naymagon, S., Naymagon, L., Wong, S. Y*., et al.* (2017). Acute graft-versus-host disease of the gut: considerations for the gastroenterologist. *Nat Rev Gastroenterol Hepatol*, *14*(12), 711-726. https://doi.org/10.1038/nrgastro.2017.126

O'Toole, D. M., and Golden, A. M. (1991). Evaluating cancer patients for rehabilitation potential. *West J Med*, *155*(4), 384-387. https://www.ncbi.nlm.nih.gov/pubmed/1771876

Oxford Textbook of Palliative Medicine. (1993). (D. Doyle, G. W. C. Hanks, and N. MacDonald, Eds.). Oxford University Press. 109.

Pasquini, M. C., and Zhu, X. (2014). *Current Uses and Outcomes of Hematopoietic Stem Cell Transplantation: 2014 CIBMTR Summary Slides.* http://www.cibmtr.org

Qiao, J., Zhu, F., Liu, Y*., et al.* (2015). Enhanced formation of neutrophil extracellular traps in patients with graft versus host disease. *Ann Hematol*, *94*(12), 2081-2083. https://doi.org/10.1007/s00277-015-2495-8

Saad, A., de Lima, M., Anand, S*., et al.* (2020). Hematopoietic Cell Transplantation, Version 2.2020, NCCN Clinical Practice Guidelines in Oncology. *J Natl Compr Canc Netw*, *18*(5), 599-634. https://doi.org/10.6004/jnccn.2020.0021

Schag, C. C., Heinrich, R. L., and Ganz, P. A. (1984). Karnofsky performance status revisited: reliability, validity, and guidelines. *J Clin Oncol*, *2*(3), 187-193. https://doi.org/10.1200/JCO.1984.2.3.187

Storb, R., Deeg, H.J., Whitehead, J., et al. (1986). Methotrexate and cyclosporine compared with cyclosporine alone for prophylaxis of acute graft versus host disease after marrow transplantation for leukemia. *N Engl J Med*, *314*, 729-735.

Yu, J., Parasuraman, S., Shah, A*., et al.* (2019). Mortality, length of stay and costs associated with acute graft-versus-host disease during hospitalization for allogeneic hematopoietic stem cell transplantation. *Curr Med Res Opin*, *35*(6), 983-988. https://doi.org/10.1080/03007995.2018.1551193

Appendix 1. Summary of Changes

Amendment 1

The following changes were made in Amendment 1 (28 August 2023) from the Original protocol (25 July 2023).

Section	Change
1.1<br>4.1.2<br>4.2.2	The secondary objective "Evaluate change in bilirubin and skin rash from baseline to Days 7, 14, 28, 56, and 180." has been removed due to redundancy.
1.1<br>0<br>5.1<br>5.5<br>6.1<br>6.4<br>7<br>7.1<br>7.4.2<br>7.6<br>9.1.3	The study design was updated to update the cohorts and include additional subgroups and the total number of participants has been updated throughout the protocol:<br>**Dose Escalation Cohorts**, Enrolled Sequentially based on protocol-specified dose escalation criteria (up to 36 participants total)<br>7 day dosing<br>• Cohort 1a: 6 participants will receive 10 mg/kg Q8H RLS-0071 for 7 days with concurrent ruxolitinib. Completion of Cohort 1a will lead to opening of Cohort 2a, if appropriate.<br>• Cohort 1b: 3 participants will receive 10 mg/kg Q8H RLS-0071 for 7 days without concurrent ruxolitinib. Opens concurrent to Cohort 1a. Completion of Cohort 1b will lead to opening of Cohort 2b, if appropriate.<br>• Cohort 2a: 6 participants will receive 40 mg/kg Q8H RLS-0071 for 7 days with concurrent ruxolitinib. Completion of Cohort 2a will lead to opening of Cohort 3, if appropriate.<br>• Cohort 2b: 3 participants will receive 40 mg/kg Q8H RLS-0071 for 7 days without concurrent ruxolitinib.<br>14 day dosing in hospitalized participants<br>• Cohort 3: 6 participants will receive 10 mg/kg Q8H RLS-0071 for 14 days with concurrent ruxolitinib. Completion of Cohort 3 will lead to opening of Cohort 4, if appropriate.<br>• Cohort 4: 6 participants will receive 40 mg/kg Q8H RLS-0071 for 14 days with concurrent ruxolitinib.<br>If a participant in cohort 3 or 4 is rolled into Cohort 5 or 6, then they will be replaced.<br>14 day dosing with 7 days inpatient and 7 day outpatient treatment<br>Participants in cohorts 3 or 4 who improve and are able to leave the hospital after 7 days of treatment with RLS-0071 likely represent a less severe population (e.g. skin disease without intestinal disease). Thus, these participants that are appropriate for hospital discharge after 7 days are a reasonable population to segregate into outpatient management as would normally be done for participants well enough to go home and are desiring to go home. These less

Section	Change
	severe participants in Cohorts 3 and 4 will be given the opportunity to roll over into the following cohorts. Determination of eligibility and appropriateness to roll over will be made by the PI in discussion with the participant as well as the Medical Monitor. These participants will be followed as a separate cohort to determine disease characteristics that are likely different from those requiring continued hospitalization.<br>• Cohort 5 up to 3 participants will receive 10 mg/kg Q8H RLS-0071 for 7 days and then 10 mg/kg QD RLS-0071 for 7 days with concurrent ruxolitinib.<br>• Cohort 6: up to 3 participants will receive 40 mg/kg Q8H RLS-0071 for 7 days and then 40 mg/kg QD RLS-0071 for 7 days with concurrent ruxolitinib.
1.2<br>9.1.2<br>9.3.1	Additional timepoints of 1 hour (± 10 min) 4 hours (± 30 min) were added on Day 1 for the intense PK sampling group in Cohorts 1a and 2a.
0<br>9.1.2<br>9.1.3	Vital signs were updated to being collected within 10 minutes before the start of the infusion and 15 minutes, 20 minutes, and 1 hour after start of the infusion.
5.3	The typo of dose escalation has been corrected to dose expansion
7.4.6.2	Dose modifications CYP3A4 Inhibitors or Fluconazole have been added based on the Jakafi package insert.
7.6.1	The guidance for the management of infusion-related reactions has been updated.
7.6.1<br>8.2<br>9.5.1	We have clarified that CTCAEv5.0 will be used throughout the protocol
8.2<br>8.2.1	The DLT definitions have been updated based on the suggestions of the FDA
8.2.2<br>8.3	The dose escalation decision rules have been updated
8.3.1	The details for the selection of the dose escalation cohorts and the safety stopping rules have been added.
10.10	The exclusion of the device related infections has been removed from the AESI.
1.1<br>3.2	Statement added that for preclinical results to refer to the IB.
Global	General copyediting and formatting was performed.

Amendment 2

The following changes were made in Amendment 2 (09 February 2024) from Amendment 1 (28 August 2023) of the protocol.

Section	Change
Title	The EU CTR No. was added.<br>The Medical Monitor was updated from Rho to CTI
1.1	The number of anticipated study sites was updated
1.1<br>1.2<br>4.1.2<br>9.1.4	ST2/REG3a sampling was removed for Days 56 and 180.
1.1<br>6.1<br>7.4.5	Contraception changed to 'highly effective contraception' to be used until the completion of participation in the study. Follow manufacture recommendations for other medications.
10.11	Added pregnancy section to include:<br>• RLS-0071 will be discontinued and the pregnancy should be reported to the CRO/Sponsor<br>• Pregnant participants to be followed until outcome<br>• Pregnant partner to be followed until outcome if she consents
1.2<br>9.1.2<br>9.1.3<br>9.5.2.1	Vital signs were updated to being collected pre dose and 10 minutes post dose for each dose.
1.1<br>6.2	Exclusion criteria updated to include:<br>• Significant liver disease that is unrelated to GvHD<br>• Active sepsis
10.2	Placebo reference was deleted.
10.5	'Not related' added to the causality categories in Table 18.
1.2	Updated Table 2 to 42 total doses for the Study drug row.
1.1<br>1.2<br>5.1<br>9.1.1	Amended optional endoscopy to Endoscopy (if performed as part of standard of care).
9.5.2.4	Removed method for eGFR from Table 17 footnote.
5.1	Added Figure 1for illustrating the treatment cohorts.

Section	Change
1.1<br>1.2<br>4.1.2<br>4.2.2<br>9.2.9.2	The following changes were made:<br>• Removed 'opioid pain medication use' language including Section 9.2.9.3.<br>• Modified 'lower GI' pain to 'abdominal' pain
1.1<br>1.2<br>4.1.2<br>4.1.3<br>4.2.2<br>4.2.3<br>5.1<br>7.5.2<br>9.1.4<br>9.2.2	Updated the language regarding corticosteroid usage and tapering methodology
1.1<br>5.1<br>7.4.3<br>7.4.4	Prohibition of changes to concomitant medications for GvHD and other conditions during RLS-0071 treatment has been updated.
1.1<br>3.2	Removed references to other studies with RLS-0071.
Global	General copyediting and formatting was performed.

Amendment 3

The following changes were made in Amendment 3 (08 April 2024) from Amendment 2 (09 February 2024) of the protocol.

Section	Change
1.1<br>6.1<br>7.4.5	Based on the Agency's comment and the guidance FDA referenced, ReAlta has updated the timing of contraception to continue for 3 months from the end of treatment for males and 6 months for females.
1.1<br>5.1<br>7.4.4<br>7.5.3	The schedule for tapering prophylactic medications has been updated throughout the protocol for consistency.
1.1<br>1.2<br>5.1	The tapering of corticosteroids was edited for clarity and to avoid confusion about the intent as well as the permitted circumstances relating to the more rapid steroid tapering options.

Section	Change
7.4.1<br>7.5.2<br>7.6	
1.1<br>4.1.3	The study objectives were updated to match Section 4.1.3 for consistency
7.4.3	Updates to the treatments for GvHD for consistency throughout the protocol.
Global	General copyediting and formatting was done.

Amendment 4

The following changes were made in Amendment 4 (16 April 2024) from Amendment 3 (08 April 2024) of the protocol.

Section	Change
1.1<br>5.1<br>7.4.3	We have provided additional clarification and made it clear that RLS-0071 will be discontinued if alternative treatment is required and that this is considered a treatment failure.
Global	General copyediting and formatting were done.

Amendment 5

The following changes were made in Amendment 5 (08 July 2024) from Amendment 4 (16 April 2024) of the protocol.

Section	Change
1.1<br>1.2<br>5.1<br>7.5.4	We have indicated that ruxolitinib can be tapered during the study and prior to 180 days if clinically indicated.
1.2<br>9.1.1<br>9.1.2<br>9.1.3<br>9.1.4<br>9.5.2.4	We have added in the collection of coagulation labs at Screening, Day 7, Day 14, and Day 28.
1.2<br>9.1.2<br>9.1.3<br>9.3.1	The collection of PK collection was corrected to Day 3 to match the timing of Doses 7/8.

Section	Change
1.2<br>9.1.2<br>9.1.3<br>9.3.1	The collection of PK blood sampling was changed for Day 3 to include Dose 9 and Day 7 to include Doses 19/20.
6.1	We have updated inclusion criteria #12 to ≥40 kg for consistency with the criteria in the synopsis in Section 1.1.
6.2	Edited exclusion criteria #12 to allow enrollment of participants with acute kidney injury resulting in an eGRF <60 mL/min/1.73m^2
7.1	The dosing in Table 4 was updated for accuracy and consistency.
7.4.5	Exclusion of women donating eggs within 6 months after the last dose of study intervention was added.
7.4.7	The exclusion of use of investigational products was updated from 3 months to 1 month for consistency with exclusion criteria #14 in Section 6.2.
8.2	Dose limiting toxicity definition was updated
8.3.1	A protocol amendment prior to starting the dose expansion cohort was added.
9.1.3	With or without ruxolitinib was removed as the 14-day dosing Cohorts (Cohort 3 and Cohort 4) are both dosing of RLS-0071 with ruxolitinib.
9.5.2.4	Footnote added to allow the use of urinalysis lab.
10.2	Spontaneous abortion or fetal death were added to the definitions of an SAE.<br>Other situations was updated to Medically important events.
Global	General copyediting and formatting were done.

Amendment 6

The following changes were made in Amendment 6 (20 March 2025) from Amendment 5 (08 July 2024) of the protocol.

Section	Change
1.1, 6.2	Edited exclusion criteria #13 to exclude participants with severe kidney disease, with an estimated glomerular filtration rate (eGFR) < 30 mL/min/1.73m^2 (chronic kidney disease [CKD] Stages 4-5)
7.4.7	Provided clarification regarding eligibility of participants taking investigational agents. Text was updated to state:<br>Participants who received any investigational agent (i.e., no FDA approved label) within 5 half-lives prior to the Screening visit are excluded. Off-label or investigational use of FDA approved medications for indications other than GVHD is permitted.

Amendment 7

The following changes were made in Amendment 7 (07 April 2025) from Amendment 6 (20 March 2025) of the protocol.

Section	Change
Title Page	Updated the study Medical Monitor per personnel change made 3 April 2025
7.4.2<br>7.6.2	Added dose modification for ruxolitinib for participants with moderate renal impairment
7.4.6.2<br>7.6.3	Moved dose modification for ruxolitinib for participants taking concomitant Strong CYP3A4 Inhibitors or Fluconazole for consistency
8.2.2	Clarified that enrollment will pause for DSMB review after the first 3 participants per cohort are enrolled.

Amendment 8

The following changes were made in Amendment 8 (29 April 2025) from Amendment 7 (07 April 2025) of the protocol.

Section	Change
1.1<br>6.1<br>9.1.1	Patients with Stage 3 or Stage 4 liver aGvHD are excluded from the study
1.1<br>6.1<br>6.4	Karnofsky Performance Status was added to the inclusion with participants with a score lower than 50 eligible after discussion with the Medical Monitor
1.1<br>6.1	Revised inclusion criteria to expand criteria:<br>At the time of study screening prior to RLS-0071 initiation:<br>a. Neutrophil count >1000/mL (not supported by growth factor supplementation, e.g., filgrastim);<br>b. Platelet count >50,000/ml (not supported by platelet transfusions);<br>c. Aspartame aminotransferase (AST) and alanine aminotransferase (ALT) < 5 times the upper limit of normal (ULN);<br>d. Serum bilirubin < 6mg/dL; and<br>e. Adequate coagulation function (e.g., prothrombin time (PT)/international normalized ratio (INR) and activated partial thromboplastin time (aPPT)/partial thromboplastin time (PTT) <2 X ULN.
1.1<br>6.2	Updated the exclusion criteria to exclude patients that oxygen supplementation instead of 50% oxygen
1.1<br>5.1<br>7	The number of participants was updated to include 9 participants in Cohort 1a.
1.1<br>5.1<br>7<br>1.1<br>1.2<br>5.1<br>7<br>7.1<br>7.6<br>9.1.2<br>9.1.3	The cohorts have been updated to indicate that Cohort 2a and 2b will be 14 day dosing and the dosing levels have been lowered. The cohorts have been updated as the dosing has changed with escalation to 20 mg/kg Q8H instead of 40 mg/kg Q8H. The new cohorts are:<br>• Cohort 2a and 2b: 10 mg/kg Q8H for 14 days,<br>• Cohort 3: 20 mg/kg Q8H for 14 days<br>• Cohort 4: 10 mg/kg Q8H for 7 to 13 days and 10 mg/kg QD to complete 14 days<br>• Cohort 5: 20 mg/kg Q8H for 7 to 13 days and 20 mg/kg QD to complete 14 days
1.1<br>5.1<br>7<br>8.3	Clarified that additional participants may be added to any cohort based on DSMB recommendation.
1.2<br>7.4.5	Clarified that FSH should be measured prior to or at screening to document post-menopausal status for women in the absence of a clear postmenopausal history.

Section	Change
1.1<br>6.1<br>9.1.1	Patients with Stage 3 or Stage 4 liver aGvHD are excluded from the study
1.1<br>6.1<br>6.4	Karnofsky Performance Status was added to the inclusion with participants with a score lower than 50 eligible after discussion with the Medical Monitor
1.1<br>6.1	Revised inclusion criteria to expand criteria:<br>At the time of study screening prior to RLS-0071 initiation:<br>a. Neutrophil count >1000/mL (not supported by growth factor supplementation, e.g., filgrastim);<br>b. Platelet count >50,000/ml (not supported by platelet transfusions);<br>c. Aspartame aminotransferase (AST) and alanine aminotransferase (ALT) < 5 times the upper limit of normal (ULN);<br>d. Serum bilirubin < 6mg/dL; and<br>e. Adequate coagulation function (e.g., prothrombin time (PT)/international normalized ratio (INR) and activated partial thromboplastin time (aPPT)/partial thromboplastin time (PTT) <2 X ULN.
1.1<br>6.2	Updated the exclusion criteria to exclude patients that oxygen supplementation instead of 50% oxygen
1.1<br>5.1<br>7	The number of participants was updated to include 9 participants in Cohort 1a.
1.1<br>5.1<br>7<br>1.1<br>1.2<br>5.1<br>7<br>7.1<br>7.6<br>9.1.2<br>9.1.3	The cohorts have been updated to indicate that Cohort 2a and 2b will be 14 day dosing and the dosing levels have been lowered. The cohorts have been updated as the dosing has changed with escalation to 20 mg/kg Q8H instead of 40 mg/kg Q8H. The new cohorts are:<br>• Cohort 2a and 2b: 10 mg/kg Q8H for 14 days,<br>• Cohort 3: 20 mg/kg Q8H for 14 days<br>• Cohort 4: 10 mg/kg Q8H for 7 to 13 days and 10 mg/kg QD to complete 14 days<br>• Cohort 5: 20 mg/kg Q8H for 7 to 13 days and 20 mg/kg QD to complete 14 days
7.5.2	Figure 2 was replaced to match the size of the corticosteroid taper options
7.6.2	Corrected table title to indicate renal impairment and removed the statement that no RLS-0071 dose modifications are allowed.
7.6.3	Removed the statement that no RLS-0071 dose modifications are allowed.
8.3.1	Clarified that the dose escalation cohort will proceed with the selected regimen(s) that is operationally implemented

Section	Change
1.1<br>6.1<br>9.1.1	Patients with Stage 3 or Stage 4 liver aGvHD are excluded from the study
1.1<br>6.1<br>6.4	Karnofsky Performance Status was added to the inclusion with participants with a score lower than 50 eligible after discussion with the Medical Monitor
1.1<br>6.1	Revised inclusion criteria to expand criteria:<br>At the time of study screening prior to RLS-0071 initiation:<br>a. Neutrophil count >1000/mL (not supported by growth factor supplementation, e.g., filgrastim);<br>b. Platelet count >50,000/ml (not supported by platelet transfusions);<br>c. Aspartame aminotransferase (AST) and alanine aminotransferase (ALT) < 5 times the upper limit of normal (ULN);<br>d. Serum bilirubin < 6mg/dL; and<br>e. Adequate coagulation function (e.g., prothrombin time (PT)/international normalized ratio (INR) and activated partial thromboplastin time (aPPT)/partial thromboplastin time (PTT) <2 X ULN.
1.1<br>6.2	Updated the exclusion criteria to exclude patients that oxygen supplementation instead of 50% oxygen
1.1<br>5.1<br>7	The number of participants was updated to include 9 participants in Cohort 1a.
1.1<br>5.1<br>7<br>1.1<br>1.2<br>5.1<br>7<br>7.1<br>7.6<br>9.1.2<br>9.1.3	The cohorts have been updated to indicate that Cohort 2a and 2b will be 14 day dosing and the dosing levels have been lowered. The cohorts have been updated as the dosing has changed with escalation to 20 mg/kg Q8H instead of 40 mg/kg Q8H. The new cohorts are:<br>• Cohort 2a and 2b: 10 mg/kg Q8H for 14 days,<br>• Cohort 3: 20 mg/kg Q8H for 14 days<br>• Cohort 4: 10 mg/kg Q8H for 7 to 13 days and 10 mg/kg QD to complete 14 days<br>• Cohort 5: 20 mg/kg Q8H for 7 to 13 days and 20 mg/kg QD to complete 14 days
Global	Changed patient to participant throughout and updated drug name to RLS-0071 throughout the protocol for consistency.
